# Supplementary material for: Quantitative analysis of medical students’ and physicians’ knowledge of degenerative cervical myelopathy
Source: BMJ Open. 2020 Jan 12;10(1):e028455. doi: 10.1136/bmjopen-2018-028455 (PMC7044983; doi:10.1136/bmjopen-2018-028455)
Supplement: Supplementary data [file bmjopen-2018-028455supp001.pdf]

Passmedicine questions

Authors
1. Mueez Waqar
2. Jane Wilcock
3. Jayne Garner
4. Benjamin M Davies
5. Mark RN Kotter

Introduction
The following is a set of questions prepared for the degenerative cervical myelopathy education initiative. We would request a link to our website - www.myelopathy.org, our website to promote this initiative, below these questions.

Methods
Questions have been prepared as follows:
- Clinical presentation - focussing on differential diagnosis: 5 MCQs, 2 EMQs
- Assessment - focussing on examination and workup: 5 MCQs, 2 EMQs
- Management - focussing on referral pathways and follow-up: 5 MCQs, 2 EMQs

These questions are targeted to the following question banks:
- MRCGP
- MSRA
- Medical finals

Output
The aim of this initiative is to provide data on clinician knowledge on degenerative cervical myelopathy. We would like to analyse the following metrics based on these questions:
- Distribution of answers on 1st attempt
- Distribution of answers on 2nd attempt

In addition, we would like to compare the correct response rate of this sample to existing questions in the question-bank along the following themes:
- Multiple sclerosis
- Cauda equina syndrome
- Diabetes mellitus

Theme 1: Clinical presentation

A 65-year-old female reports progressive unsteadiness, mild weakness, pain and paraesthesias in her legs over the last 2-3 months. She reports no symptoms in her upper limbs and there is no history of trauma. Examination reveals increased lower limb tone with brisk reflexes. Which of the following is the most likely explanation for her symptoms?

A. Motor neuron disease
B. Degenerative cervical myelopathy
C. Multiple sclerosis
D. Syringomyelia
E. Cauda equina syndrome

Answer: B

Degenerative cervical myelopathy (DCM) refers to cervical spinal cord compression due to cervical spondylosis. The term replaces CSM (Cervical Spondylotic Myelopathy). Patients with DCM can present with predominantly lower limb symptoms. Examination findings, especially early on, are often subtle. DCM is the most common cause of non-traumatic paraparesis in developed countries and becomes more common with age [2]; it is estimated to have a prevalence of 5% in over 50-year-olds [3, 4]. In a series of patients presenting to Neurology outpatients, it was twice as common as Multiple Sclerosis [2]. Early diagnosis is key to ensuring good clinical outcomes. Currently most patients wait over 2 years for a diagnosis.

Incorrect options:

- Motor neuron disease is a disease of motor neurons, with mixed upper and lower motor features. Patients are classically described to have brisk reflexes in wasted and fasciculating limbs. As a rule, motor symptoms of weakness predominate and patients do not have sensory findings.
- Multiple sclerosis can also cause paraparesis. As a disease of the central nervous system, the paraparesis is usually associated with upper motor neuron signs. A first presentation of multiple sclerosis is most common between the ages of 20 and 40, younger than this patient. Degenerative Cervical Myelopathy is also more common with age and more common than MS overall, making that diagnosis more likely.
- Syringomyelia refers to the development of a syrinx in the spinal cord. It presents with a central cord syndrome, with predominantly upper limb signs. It is a relatively uncommon condition.
- Cauda equina syndrome results from compression of the cauda equina and classically includes leg weakness, saddle anaesthesia and sphincter disturbance. It is usually an acute syndrome with progressive signs. It is also a relatively uncommon condition.

References:

1. Kjaer P, Leboeuf-Yde C, Korsholm L, Sorensen JS, Bendix T. Magnetic resonance imaging and low back pain in adults: a diagnostic imaging study of 40-year-old men and women. Spine. 2005 May 15;30(10):1173–80.
2. Moore AP, Blumhardt LD. A prospective survey of the causes of non-traumatic spastic paraparesis and tetraparesis in 585 patients. Spinal Cord. 1997 Jun;35(6):361-7.
3. Kovalova I, Kerkovsky M, Kadanka Z, Kadanka Z, Nemec M, Jurova B, et al. Prevalence and Imaging Characteristics of Non-Myelopathic and Myelopathic Spondylotic Cervical Cord Compression. Spine. 2016 Aug 9.
4. Bednarik J, Kadanka Z, Dusek L, Kerkovsky M, Vohanka S, Novotny O, et al. Presymptomatic spondylotic cervical myelopathy: an updated predictive model. Eur Spine J. 2008 Mar;17(3):421–31.

A 73-year-old male presents with progressively worsening gait and urinary urgency. He is diagnosed with degenerative cervical myelopathy. Which ONE of the following is true regarding this condition?

A. Smoking is not a risk factor in isolation
B. Asians can have a different underlying aetiology than caucasians
C. Bowel and bladders symptoms are rare and should prompt consideration of cauda equina syndrome
D. Most patients present with a classic triad of neck pain, finger paraesthesias and weak legs
E. Family history is of limited value

Answer: B

Asian populations have a higher rate of ossification of the posterior longitudinal ligament (OPLL), which can result in myelopathy.

Degenerative cervical myelopathy (DCM) has a number of risk factors, which include smoking due to its effects on the intervertebral discs (A, false), genetics (option E, false) and occupation - those exposing patients to high axial loading [1].

The presentation of DCM is very variable (option D, false). Early symptoms are often subtle and can vary in severity day to day, making the disease difficult to detect initially. However as a progressive condition, worsening, deteriorating or new symptoms should be a warning sign.

DCM symptoms can include any combination of [1]:
- Pain (affecting the neck, upper or lower limbs)
- Loss of motor function (loss of digital dexterity, preventing simple tasks such as holding a fork or doing up their shirt buttons, arm or leg weakness/stiffness leading to impaired gait and imbalance
- Loss of sensory function causing numbness
- Loss of autonomic function (urinary or faecal incontinence and/or impotence) - these can occur and do not necessarily suggest cauda equina syndrome in the absence of other hallmarks of that condition

The most common symptoms at presentation of DCM are unknown, but in one series 50% of patients were initially incorrectly diagnosed and sometimes treated for carpal tunnel syndrome [2].

References
1. Baron EM, Young WF. Cervical spondylotic myelopathy: a brief review of its pathophysiology, clinical course, and diagnosis. Neurosurgery. 2007 Jan;60(1 Supp1 1):S35-41.

2. Behrbalk E, Salame K, Regev GJ, Keynan O, Boszczyk B, Lidar Z. Delayed diagnosis of cervical spondylotic myelopathy by primary care physicians. Neurosurg Focus. 2013 Jul;35(1):E1.

Stem 1: A 60-year-old gentleman with a background of hypertension and high cholesterol presents with a long history of neck pain, lower limb stiffness and urinary hesitancy. Which of the following is most likely?

Stem 2: A 56-year-old gentleman presents with lower limb stiffness and imbalance. His only past medical history of note is carpal tunnel syndrome that was diagnosed a year ago on clinical grounds and has been refractory to treatment with splints and steroid injections. Which of the following is most likely?

A. Cauda equina syndrome
B. Subacute combined degeneration of the cord
C. Degenerative cervical myelopathy
D. Parkinson's disease
E. Multiple sclerosis

Answer: C

The presentation of degenerative cervical myelopathy [DCM] is variable. Early symptoms are often subtle and can vary in severity day to day, making the disease difficult to detect initially. However as a progressive condition, worsening, deteriorating or new symptoms should be a warning sign.

DCM symptoms can include any combination of [1]:
- Pain (affecting the neck, upper or lower limbs)
- Loss of motor function (loss of digital dexterity, preventing simple tasks such as holding a fork or doing up their shirt buttons, arm or leg weakness/stiffness leading to impaired gait, imbalance and
- Loss of sensory function causing numbness
- Loss of autonomic function (urinary or faecal incontinence and/or impotence).

The most common symptoms at presentation of DCM are unknown, but in one series 50% of patients were initially incorrectly diagnosed and sometimes treated for carpal tunnel syndrome [2].

Other answers:
- Cauda equina syndrome results from compression of the cauda equina and classically includes leg weakness, saddle anaesthesia and sphincter disturbance. It is usually an acute syndrome with progressive signs. It does not cause leg stiffness.
- Subacute combined degeneration of the cord results from long-standing vitamin B12 deficiency, classically presenting as a posterior cord syndrome – with impaired proprioception. It can feature both upper and lower motor neuron signs. B12 deficiency can be associated with several neurological features. These include a myelopathy (classically the subacute combined degeneration of the cord), neuropathy and paraesthesias without neurological signs [3]. Subacute combined degeneration is extremely rare in developed countries, though in tropical countries it is frequently the commonest cause of non-traumatic myelopathy [4].

- Idiopathic Parkinson's disease is a tetrad of Tremor, Rigidity, Akinesia and Postural Instability (this can be remembered using the TRAP mneumonic). In the early stages pain is not a typical feature and it does not cause numbness.
- Multiple Sclerosis [MS] can have a variable presentation, with both sensory and motor symptoms and signs. Inflammatory changes are often present at multiple sites, which can cause symptoms at more than one site; a 'dissociated sensory loss', that is numbness at different and unlinked sites, is a hallmark of MS. Often patients will recall previous episodes of odd neurological deficits, which resolved. MS predominantly affects woman (3-4 times common) and usually presents before the age of 45.

References:

1. Baron EM, Young WF. Cervical spondylotic myelopathy: a brief review of its pathophysiology, clinical course, and diagnosis. Neurosurgery. 2007 Jan;60(1 Supp1 1):S35-41.
2. Behrbalk E, Salame K, Regev GJ, Keynan O, Boszczyk B, Lidar Z. Delayed diagnosis of cervical spondylotic myelopathy by primary care physicians. Neurosurg Focus. 2013 Jul;35(1):E1.
3. Kumar N1. Neurologic aspects of cobalamin (B12) deficiency. Handb Clin Neurol. 2014;120:915-26.
4. Pinto WB, de Souza PV, de Albuquerque MV, Dutra LA, Pedroso JL, Barsottini OG. Clinical and epidemiological profiles of non-traumatic myelopathies. Arq Neuropsiquiatr. 2016 Feb;74(2):161-5.

Select the most likely diagnosis for the following clinical scenarios. Each option can be chosen once or not at all.

A. Multiple sclerosis
B. Aortic aneurysm
C. Fractured clavicle
D. Peripheral neuropathy
E. Degenerative cervical myelopathy
F. Median nerve entrapment
G. Ulnar nerve entrapment
H. Lumbar canal stenosis
I. Saturday night palsy
J. Adhesive Capsulitis

1. A 70 year-old man has pain and weakness in both legs on walking. It settles with rest.
2. A 54 year-old female complains of right hand pain radiating into her thumb, index and middle finger. It often wakes her up from sleep.
3. A 54 year-old female presents with a loss of dexterity in both hands. She has been struggling to type at work and use her mobile phone. Her symptoms have been deteriorating gradually over the preceding months.

Answers

- 1H - lumbar spinal canal stenosis or vascular claudication from peripheral vascular disease are likely. Peripheral vascular disease is more common, but not an option here.
- 2F - Carpal tunnel syndrome results from median nerve compression at the wrist, within the carpal tunnel, and results in lower motor neuron signs, with thenar muscle wasting and weakness of the LOAF muscles (lateral lumbricals, opponens pollicis, abductor pollicis brevis and flexor policis brevis). Patients can have paraesthesias in the median nerve distribution, classically at night. Tinel's test and Phalen's test can be positive.
- 3E - Degenerative cervical myelopathy leads to loss of fine motor function in both upper limbs. There is a delay in diagnosis of degenerative cervical myelopathy, which is estimated to be >2 years in some studies [1]. It is most commonly misdiagnosed as carpal tunnel syndrome and in one study, 43% of patients who underwent surgery for degenerative cervical myelopathy, had been initially diagnosed with carpal tunnel syndrome [1].

References:

1. Behrbalk E, Salame K, Regev GJ, Keynan O, Boszczyk B, Lidar Z. Delayed diagnosis of cervical spondylotic myelopathy by primary care physicians. Neurosurg Focus. 2013 Jul;35(1):E1.

Select the most likely diagnosis for the following clinical scenarios. Each option can be chosen once or not at all.

A. Multiple sclerosis
B. Aortic aneurysm
C. Fractured clavicle
D. Peripheral neuropathy
E. Degenerative cervical myelopathy
F. Median nerve entrapment
G. Ulnar nerve entrapment
H. Lumbar canal stenosis
I. Saturday night palsy
J. Adhesive Capsulitis

1. A 60 year-old male presents with clumsy hands. He has been dropping cups around the house. His wife complains he doesn't answer his mobile as he struggles to use it. His symptoms have been gradually deteriorating over the preceding months.
2. A 32 year-old female presents with a 3 day history of altered sensation on her left foot and right forearm. On examination she has clonus in both legs and has hyperreflexia in all limbs.
3. A 45 year-old female presents with stiffness and pain in her left shoulder, which started around a month ago. She had a similar episode that resolved by itself. Examination reveals limited external rotation.

Answers

- 1E - Degenerative cervical myelopathy leads to loss of fine motor function in both upper limbs. There is a delay in diagnosis of degenerative cervical myelopathy, which is estimated to be >2 years in some studies [1]. It is most commonly misdiagnosed as carpal tunnel syndrome and in one study, 43% of patients who underwent surgery for degenerative cervical myelopathy, had been initially diagnosed with carpal tunnel syndrome [1].
- 2A - Multiple sclerosis (MS) can have a variable presentation, affecting both the sensory and/or motor systems. Inflammatory changes are often present at multiple sites, which can cause symptoms at more than one site; a 'dissociated sensory loss', that is numbness at different and unlinked sites, is a hallmark of MS. Often patients will recall previous episodes of odd neurological deficits, which resolved. MS predominantly affects woman (3-4 times common) and usually presents before the age of 45.
- 3J - Adhesive capsulitis or 'frozen shoulder' is most common in the fifth or sixth decade of life. Women are more likely to be affected than men. It is also more common in patients with diabetes mellitus.

References:

1. Behrbalk E, Salame K, Regev GJ, Keynan O, Boszczyk B, Lidar Z. Delayed diagnosis of cervical spondylotic myelopathy by primary care physicians. Neurosurg Focus. 2013 Jul;35(1):E1.

Theme 2: Assessment

A 58 year old gentleman presents with left sided paraesthesias affecting his thumb and first finger. He complains of grip weakness and dropping objects unintentionally. On examination, there is wasting over the thenar eminence. Which of the following signs would suggest a diagnosis other than carpal tunnel syndrome?

A. Positive Hoffman's sign
B. Thenar muscle wasting
C. Unilateral weakness of pincer grip
D. Positive Phalen's test
E. Positive Tinnel's test

Answer: A

A positive Hoffman's sign is a sign of upper motor neuron dysfunction and points to a disease of the central nervous system - in this case from the history degenerative cervical myelopathy [DCM] affecting the cervical spinal cord is most likely. To elicit it, the examiner should flick the patient's distal phalanx (usually of the middle finger) to cause momentary flexion. A positive sign is exaggerated flexion of the thumb.

DCM is often missed initially and there is a delay in the diagnosis of this condition by >2 years in some studies [1]. This is a problem as delayed treatment limits recovery. It is most commonly misdiagnosed as carpal tunnel syndrome and in one study, 43% of patients who underwent surgery for degenerative cervical myelopathy, had been initially diagnosed with carpal tunnel syndrome [1]. DCM is therefore an important differential in patients suspected to have Carpal Tunnel Syndrome [CTS].

CTS is a disease of the peripheral nervous system, resulting from median nerve compression at the wrist inside the carpal tunnel. It therefore affects only the aspects of the hand innervated by the median nerve:

- Sensation; Thumb / Index / Middle Finger. This typically manifests as intermittent pain or parasthesiae.
- Motor; 'LOAF Muscles'(lateral lumbricals, opponens pollicis, abductor pollicis brevis and flexor policis brevis). Motor signs are less commonly seen with presentations of CTS, but wasting of the thenar eminence may be present.

Tinel's test and Phalen's test can be positive, but not always. Both tests aim to increase the pressure within the carpal tunnel, to try to exacerbate symptoms; Tinel's test via tapping on it and Phalen's test by sustained full flexion of the wrist.

In focal central nervous system disorders, like DCM, examination features are known to have low sensitivity but high specificity [2]. As a disease of the cervical spinal cord, DCM can affect the sensory, motor and autonomic nervous systems from the neck downwards. Motor signs will be upper motor neuron signs such as increased toned, hyper-reflexia and pyramidal weakness. Note that the neurological signs of DCM are

often subtle initially and easily missed, but as a progressive condition they are likely to get worse [3]. Therefore detecting early DCM can be challenging. A high index of suspicion, alongside a comprehensive neurological examination and monitoring for progression is required.

References:

1. Behrbalk E, Salame K, Regev GJ, Keynan O, Boszczyk B, Lidar Z. Delayed diagnosis of cervical spondylotic myelopathy by primary care physicians. Neurosurg Focus. 2013 Jul;35(1):E1.
2. Nicholl DJ, Appleton JP. Clinical neurology: why this still matters in the 21st century. Journal of Neurology, Neurosurgery & Psychiatry 2015;86:229-33.
3. Baron EM, Young WF. Cervical spondylotic myelopathy: a brief review of its pathophysiology, clinical course, and diagnosis. Neurosurgery. 2007 Jan;60(1 Supp1 1):S35-41.

Which of the following statements is most accurate regarding the usefulness of cervical spine radiographs (X-rays) in the assessment of degenerative cervical myelopathy (DCM)?

A. Cervical spine radiographs should be obtained in all patients suspected of having DCM.
B. Where DCM is suspected, AP (anteroposterior), lateral *and* oblique cervical spine radiographs should be requested
C. Cervical spine radiographs are a useful first line investigation where a diagnosis of DCM is suspected
D. Cervical spine radiographs have a low sensitivity but high specificity for DCM
E. Cervical spine radiographs cannot diagnose DCM

Answer: E

Radiographs are of limited value where a diagnosis of degenerative cervical myelopathy is suspected [1] as they cannot visualise the soft tissue, such as the spinal cord.

Spine radiographs have a high sensitivity, but limited specificity to diagnose most spinal conditions. Oblique spine radiographs are usually requested in the lumbar spine region to pick up defects in the pars interarticularis. They have no value in setting of DCM.

The finding of spondylosis is common in spinal x-rays of adults over 40 [2]. Its absence does not exclude neural compression.

Degenerative Cervical Myelopathy [DCM] is spinal cord compression due to degenerative changes of the surrounding spinal structures; e.g. from disc herniation, ligament hypertrophy or calcification, or osteophytes. Therefore in order to visualise these structures, a MRI is gold standard and first line.

Again the presence of such degenerative changes is common on MRI; in one study, 57% of patients older than 64 years of age had disc bulging, though only 26% had spinal cord compression [3]. Therefore a diagnosis of DCM requires the finding of MRI compression in concert with appropriate signs and symptoms.

References

1. Nouri A, Tetreault L, Singh A, Karadimas SK, Fehlings MG. Degenerative Cervical Myelopathy: Epidemiology, Genetics, and Pathogenesis. Spine (Phila Pa 1976). 2015 Jun 15;40(12):E675-93.
2. Baron EM, Young WF. Cervical spondylotic myelopathy: a brief review of its pathophysiology, clinical course, and diagnosis. Neurosurgery. 2007 Jan;60(1 Supp1 1):S35-41.
3. Teresi LM, Lufkin RB, Reicher MA, Moffit BJ, Vinuela FV, Wilson GM, Bentson JR, Hanafee WN: Asymptomatic degenerative disk disease and spondylosis of the cervical spine: MR imaging. Radiology 164:83–88, 1987.

A 67-year-old male undergoes investigations for bilateral paraesthesia in the radial aspects of both hands, over the thumbs and first fingers. He also has paraesthesia in the lateral aspects of both forearms and lower limb spasticity. Blood tests reveal a HBA1c of 46. He undergoes nerve conduction studies and EMG with evidence of denervation. Which ONE of the following diagnoses is most likely?

A. Bilateral carpal tunnel syndrome
B. Degenerative cervical myelopathy
C. Multiple sclerosis
D. Syringomyelia
E. Diabetic neuropathy

Answer: B

This patient's twitches are probably fibrillations, a sign of lower motor neuron dysfunction. This is confirmed on the neurophysiology report, with evidence of denervation. His symptoms are predominantly in the C6 dermatome distribution bilaterally. Although median nerve compression at the elbow bilaterally could in theory produce his symptoms, it would be less likely to explain his symptoms given his age. He is likely to have degenerative cervical myelopathy. This condition is associated with a delay in diagnosis, estimated to be >2 years in some studies [1].

Patients with degenerative cervical myelopathy can present with a number of problems [2]:

- Pain/stiffness: affecting the neck, upper and/or lower limbs. L'hermitte's sign is a sharp pain radiating down the spine on flexion of the neck, which is classically associated with multiple sclerosis, though it can occur in cervical myelopathy.
- Loss of function: Clumsiness (e.g. can't do shirt buttons, hold cup), leg weakness leading to impaired gait, imbalance and falls.
- Sphincter disturbance: this can range from frequency and urgency to incontinence.

Neurological examination can reveal lower motor neuron signs at the level of the lesion and upper motor neuron signs below. Note that neurological signs can be subtle and a high degree of suspicion is needed [2].

The other answers in this question are unlikely for the following reasons:

- A: bilateral carpal tunnel syndrome would not cause forearm symptoms. Carpal tunnel syndrome results from median nerve compression at the wrist and results in a lower motor neuron picture, with thenar muscle wasting and weakness of the LOAF muscles (lateral lumbricals, opponens pollicis, abductor pollicis brevis and flexor policis brevis). Tinel's test and Phalen's test can be positive.
- C: multiple sclerosis (MS) is rare in this age group. MS predominantly affects woman (3-4 times common) and usually presents before the age of 45. It can have a variable presentation, affecting both the sensory and/or motor systems. Inflammatory changes are often present at multiple sites, which can cause symptoms at more than one site; a 'dissociated sensory loss', that is numbness at different and unlinked

sites, is a hallmark of MS. Often patients will recall previous episodes of odd neurological deficits, which resolved.

- D: Syringomyelia refers to the development of a syrinx in the spinal cord. It presents with a central cord syndrome, with predominantly upper limb signs. It is a relatively uncommon condition.
- E: His HBA1c is not within the diagnostic range of diabetes mellitus. Diabetes mellitus can cause a peripheral neuropathy presenting in a glove and stocking distribution, as well as neuropathy of peripheral nerves - mononeuritis multiplex.

References:

1. Behrbalk E, Salame K, Regev GJ, Keynan O, Boszczyk B, Lidar Z. Delayed diagnosis of cervical spondylotic myelopathy by primary care physicians. Neurosurg Focus. 2013 Jul;35(1):E1.
2. Baron EM, Young WF. Cervical spondylotic myelopathy: a brief review of its pathophysiology, clinical course, and diagnosis. Neurosurgery. 2007 Jan;60(1 Supp1 1):S35-41.

Which of the following investigations is the most important for diagnosing degenerative cervical myelopathy ?

A. Nerve conduction studies and EMG
B. MRI Cervical spine
C. CT myelogram
D. CT C-spine
E. AP and lateral C-spine radiographs

Answer: B

An MRI of the cervical spine is the gold standard test where cervical myelopathy is suspected. It may reveal disc degeneration and ligament hypertrophy, with accompanying cord signal change.

Other answers:
- CT imaging is reserved for patients with contraindications to magnetic resonance imaging. A CT myelogram is the first line investigation in this case.
- Radiographs are not clinically useful in the workup of these patients, though osteoarthritic changes (e.g. osteophytes) can be visible if they are performed.
- Other investigatons (e.g. nerve conduction studies, EMG) may be performed when the clinical picture is unclear. These can help to exclude mononeuropathies and other lower motor neuron disorders. However, where there is strong clinical suspicion and the diagnosis is suspected, an MRI of the cervical spine should be performed.

A 60-year-old gentleman with a background of lumbar spondylosis and chronic back pain presents with gradually worsening bilateral upper limb paraesthesias and leg stiffness. Which one of the investigations below is diagnostic for his likely condition?

A. Nerve conduction studies and EMG
B. MRI Cervical spine
C. MRI Lumbar Spine
D. CT C-spine
E. AP and lateral C-spine radiographs

Answer: B

The presence of upper limb neurological symptoms indicates that there is pathology either within his cervical spinal cord or brain. Brain disease is more likely to cause unilateral problems.

A MRI lumbar spine would therefore not provide a unifying diagnosis here.

In the context of known lumbar degenerative spine, degenerative cervical myelopathy is the number one differential for this presentation. An MRI of the cervical spine is the gold standard test where cervical myelopathy is suspected. It may reveal disc degeneration and ligament hypertrophy, with accompanying cord signal change. It is not uncommon for patients to suffer from tandem (cervical and lumbar) stenosis.

Other answers:

- CT imaging is reserved for patients with contraindications to magnetic resonance imaging. A CT myelogram is the first line investigation in this case
- Radiographs are not clinically useful in the workup of these patients, though osteoarthritic changes (e.g. osteophytes) can be visible if they are performed.
- Other investigatons (e.g. nerve conduction studies, EMG) may be performed when the clinical picture is unclear. These can help to exclude mononeuropathies and other lower motor neuron disorders. However, where there is strong clinical suspicion and the diagnosis is suspected, an MRI of the cervical spine should be performed.

Select the **most likely** positive examination finding for the following clinical scenarios. Each option can be chosen once, more than once or not at all.

A. Kernig's sign
B. Ankle Brachial Pressure Index
C. Tinel's test
D. Straight leg raise
E. Tongue fasciculations
F. Hoffman's sign
G. Limited external rotation of the shoulder
I. Hypothenar wasting
J. Limited internal rotation of the shoulder

1. A 70 year-old male with a background of diabetes and hypertension presents with pain and weakness in both legs on walking. It settles with rest.
2. A 54 year-old female complains of right hand pain radiating into her thumb, index and middle finger. It often wakes her up from sleep.
3. A 65 year-old female presents with a loss of dexterity in both hands. She has been struggling to type at work and use her mobile phone. Her symptoms have been deteriorating gradually over the preceding months.3

Answers
1 - B: this patient is likely to have peripheral vascular disease [PVD] given his background risk factors for this condition. The ankle brachial pressure index [ABPI] is a simple method of assessing the peripheral circulation. It is calculated by dividing systolic blood pressure in the ankle by the the systolic blood pressure in the arm. These are equal in health (ABPI = 1). The ABPI is reduced in PVD.
2 - C: this patient is likely to have carpal tunnel syndrome. This occurs due to median nerve entrapment beneath the flexor retinaculum. Clinical tests to raise carpal tunnel pressure can exacerbate symptoms and support a diagnosis. One such example is Tinel's test, includes tapping over the volar surface of the wrist joint i.e. over the carpal tunnel, may reproduce paraesthesias. A normal Tinel's test does not exclude carpal tunnel syndrome.
3 - F: this patient is likely to have degenerative cervical myelopathy [DCM], which is associated with upper motor neuron signs. Hoffman's sign is elicited by flicking the distal phalaynx of the middle finger to cause momentary flexion. A positive result is exaggerated flexion of the terminal phalanyx of the thumb. Patients with DCM often have subtle signs that are easily missed [1], but as a progressive condition, these are likely to get worse [2]. Whilst the sensitivity of signs is low (i.e. their absence does not rule out a problem), their specificity is high (i.e. there will be a problem). Therefore, in order to diagnose early DCM and improve patient outcomes, a high index of suspicion, alongside a 4comprehensive neurological examination and monitoring for progression is required.

Other signs mentioned:

- Kernig's sign refers to painful knee extension, from a position of hip flexion and knee flexion. It suggest meningeal irritation e.g. meningitis, subarachnoid haemorrhage.
- Straight leg raise: this is positively associated with radicular pathology such as disc herniation. The patient feels pain in the back when the leg is raised between 30-60 degrees.
- Limited external rotation is classically found in adhesive capsulitis. Patients have global restriction of shoulder movements, in at least two axes, though external rotation is usually the most affected and painful.

References:

1. Behrbalk E, Salame K, Regev GJ, Keynan O, Boszczyk B, Lidar Z. Delayed diagnosis of cervical spondylotic myelopathy by primary care physicians. Neurosurg Focus. 2013 Jul;35(1):E1.
2. Baron EM, Young WF. Cervical spondylotic myelopathy: a brief review of its pathophysiology, clinical course, and diagnosis. Neurosurgery. 2007 Jan;60(1 Supp1 1):S35-41.

Select the **most likely** positive examination finding for the following clinical scenarios. Each option can be chosen once, more than once or not at all.

A. Kernig's sign
B. Ankle Brachial Pressure Index
C. Tinel's test
D. Straight leg raise
E. Tongue fasciculations
F. Hoffman's sign
G. Limited external rotation of the shoulder
I. Hypothenar wasting
J. Limited internal rotation of the shoulder

1. A 60 year-old male presents with clumsy hands. He has been dropping cups around the house. His wife complains he doesn't answer his mobile as he struggles to use it. His symptoms have been gradually deteriorating over the preceding months.
2. A 32 year-old female presents with a 3 day history of altered sensation of her left foot and right forearm. She had an episode of visual loss a few months ago and says her friends have noted her eyes be flickery and jerky.
3. A 45 year-old female presents with stiffness and pain in her left shoulder, which started around a month ago. She had a similar episode that resolved by itself.

Answers
1 - F: this patient is likely to have degenerative cervical myelopathy [DCM], which is associated with upper motor neuron signs. Hoffman's sign is elicited by flicking the distal phalaynx of the middle finger to cause momentary flexion. A positive result is exaggerated flexion of the terminal phalanyx of the thumb. Patients with DCM often have subtle signs that are easily missed [1], but as a progressive condition, these are likely to get worse [2]. Whilst the sensitivity of signs is low (i.e. their absence does not rule out a problem), their specificity is high (i.e. there will be a problem). Therefore, in order to diagnose early DCM and improve patient outcomes, a high index of suspicion, alongside a comprehensive neurological examination and monitoring for progression is required.
2 - F: this patient is likely to have Multiple Sclerosis (MS). As a disease of the central nervous system, MS is usually associated with only upper motor neuron signs such as Hoffman's sign (see above). The patient's visual loss was probably secondary to optic neuritis, a common presentation of MS. Cerebellar signs are particularly common with MS and include nystagmus, which is likely to be the jerky eye movements noted by her friends.
3 - G: this patient is likely to have adhesive capsulitis. Patients have global restriction of shoulder movements, in at least two axes, though external rotation is classically described as the most affected and painful.

Other signs mentioned:
- Kernig's sign refers to painful knee extension, from a position of hip flexion and knee flexion. It suggest meningeal irritation e.g. meningitis, subarachnoid haemorrhage.

- Straight leg raise: this is positively associated with radicular pathology such as disc herniation. The patient feels pain in the back when the leg is raised between 30-60 degrees.
- The ankle brachial pressure index [ABPI] is a simple method of assessing the peripheral circulation. It is calculated by dividing systolic blood pressure in the ankle by the the systolic blood pressure in the arm. These are equal in health (ABPI = 1). The ABPI is reduced in peripheral vascular disease.
- Tinel's test includes tapping over the volar surface of the wrist joint i.e. over the carpal tunnel. This can reproduce paraesthesias in patients with carpal tunnel syndrome.

References:

1. Behrbalk E, Salame K, Regev GJ, Keynan O, Boszczyk B, Lidar Z. Delayed diagnosis of cervical spondylotic myelopathy by primary care physicians. Neurosurg Focus. 2013 Jul;35(1):E1.
2. Baron EM, Young WF. Cervical spondylotic myelopathy: a brief review of its pathophysiology, clinical course, and diagnosis. Neurosurgery. 2007 Jan;60(1 Supp1 1):S35-41.

Theme 3: Management and follow-up

A 75-year old gentleman presents with a short history of neck pain, paraesthesia in his finger tips and progressive leg weakness. Following a MRI scan of his spine, he is diagnosed with degenerative cervical myelopathy due to a C4/5 disc prolapse. Which of the following is the most appropriate management?

A. Cervical decompressive surgery
B. Cervical nerve root injection
C. Analgesia and referral to physiotherapy
D. Analgesia and review in 4 weeks time
E. Analgesia, a hard cervical collar and review in 4 weeks

Answer: A

All patients with degenerative cervical myelopathy should be urgently referred for assessment by specialist spinal services (neurosurgery or orthopaedic spinal surgery). This is due to the importance of early treatment. The timing of surgery is important, as any existing spinal cord damage can be permanent. Early treatment (within 6 months of diagnosis) offers the best chance of a full recovery but at present, most patients are presenting too late. In one study, patients averaged over 5 appointments before diagnosis, representing >2 years [1].

Currently, decompressive surgery is the only effective treatment. It has been shown to prevent disease progression. Close observation is an option for mild stable disease, but anything progressive or more severe requires surgery to prevent further deterioration. Physiotherapy should only be initiated by specialist services, as manipulation can cause more spinal cord damage.

Prompt diagnosis and onward referral are therefore key to ensuring good outcome for your patients. There are national initiatives to raise awareness of the condition to try and improve referral times (www.myelopathy.org). All of the other listed options in this question do not control the patient's primary pathology.

References:
1. Behrbalk E, Salame K, Regev GJ, et al. Delayed diagnosis of cervical spondylotic myelopathy by primary care physicians. Neurosurg Focus 2013;35:E1. doi:10.3171/2013.3.FOCUS1374

A 65-year old gentleman with a background of osteoarthritis and previous cervical laminectomy for degenerative cervical myelopathy presents with a 2-month history of worsening gait instability and urinary urgency. Which of the following is the most likely explanation for his symptoms?

A. Transverse myelitis
B. Recurrent degenerative cervical myelopathy
C. Multiple sclerosis
D. Cauda equina syndrome
E. Spinal metastases

Answer: B

- Postoperatively, patients with cervical myelopathy require ongoing follow-up as pathology can “recur” at adjacent spinal levels, which were not treated by the initial decompressive surgery. This is called adjacent segment disease. Furthermore, surgery can change spinal dynamics increasing the likelihood of other levels being affected. Patients sometimes develop mal-alignment of the spine, including kyphosis and spondylolisthesis, and this can also affect the spinal cord. All patients with recurrent symptoms should be evaluated urgently by specialist spinal services.
- Transverse myelitis usually presents more acutely than in this case, with a sensory level and upper motor neuron signs below the level affected. It can occur in patients with multiple sclerosis or Devic’s disease (neuromyelitis optica). These patients tend to also have features such as optic neuritis.
- Cauda equina syndrome results from compression of the cauda equina and classically includes leg weakness, saddle anaesthesia and sphincter disturbance. This gentleman’s history is much more likely to be in keeping with recurrent cervical myelopathy, given his background and given the subacute presentation
- Spinal metastases are uncommon, especially in a patient without a known primary. Given previous DCM, recurrence is more likely.

A 70 year old man has decompressive surgery for degenerative cervical myelopathy. Three years later he presents with neck pain and hand paraesthesias. Which one of the following management strategies is recommended?

A. Trial of neuropathic analgesia and cervical nerve root injections
B. Investigate with nerve conduction studies and EMG in the first instance
C. Urgent AP/lateral cervical spine radiographs as an MRI scan is contraindicated
D. Urgent referral to spinal surgery or neurosurgery
E. Refer to physiotherapy services

Answer: D

Postoperatively, patients with cervical myelopathy require ongoing follow-up as pathology can “recur” at adjacent spinal levels, which were not treated by the initial decompressive surgery.

Recurrent symptoms should be treated with a high degree of suspicion. Although peripheral neuropathy can occur in any patient, this should not be the diagnosis that is the most strongly suspected as delays in diagnosis and treatment of DCM affect outcomes. Therefore, B is false.

All patients with recurrent symptoms should be evaluated urgently by specialist spinal services (A and E, false). Axial spine imaging is necessary and a MRI scan is first line. In patients unable to to have a MRI, CT or CT myelogram may be considered. AP and lateral radiographs are of limited use when myelopathy is suspected (C, false).

References
1. Kong L, Cao J, Wang L, Shen Y. Prevalence of adjacent segment disease following cervical spine surgery: A PRISMA-compliant systematic review and meta-analysis. Medicine (Baltimore). 2016 Jul;95(27):e4171.

A 65-year-old gentleman is referred to neurology outpatients with arm pain, stiffness and imbalance. Following investigations he is diagnosed with degenerative cervical myelopathy. Unfortunately, he misses his next outpatient clinic due to admission with acute coronary syndrome. He attends his GP 2 months later and mentions his ongoing neurological symptoms. Which of the following is the most important next step in his care?

A. Refer to spinal surgery or neurosurgery
B. Refer for cervical nerve root injections
C. Commence neuropathic analgesia
D. Reassure the patient of his diagnosis
E. Refer for physiotherapy

Answer: A

Management of patients with cervical myelopathy should be by specialist spinal services (neurosurgery or orthopaedic spinal surgery). Decompressive surgery is the mainstay of treatment and has been shown to stop disease progression (B, false). Close observation is an option for mild stable disease, but anything progressive or more severe requires surgery to prevent further deterioration. Pre-operative physiotherapy should only be initiated by specialist services, as manipulation can cause more spinal cord damage.

The timing of surgery is important, as any existing spinal cord damage can be permanent. Treatment within 6 months offers the best chance of making a full recovery. At present most patients wait more than 2 years for a diagnosis [1].

Other incorrect options:

- Neuropathic analgesia is important for symptomatic relief but will not prevent further cord damage.
- Physiotherapy does not replace surgical opinion, it can in fact cause more spinal cord damage in patients yet to receive surgical treatment. It should therefore only be initiated by specialist services.

1. Behrbalk E, Salame K, Regev GJ, Keynan O, Boszczyk B, Lidar Z. Delayed diagnosis of cervical spondylotic myelopathy by primary care physicians. Neurosurg Focus. 2013 Jul;35(1):E1.

A 67-year old male recently attended A&E, with a 3 month history of bilateral paraesthesias and twitching affecting the thumb, first finger and lateral forearm. He denied any trauma. A MRI scan of his spine was performed and revealed cervical canal stenosis with mild cord compression. He was discharged and advised to see his GP for follow-up. Which of the following is the most appropriate initial step in management?

A. Refer to spinal surgery services
B. Refer for locally commissioned cervical root injections and review after 6 weeks
C. Enlist on the weekly minor ops clinic for carpal tunnel decompression
D. Commence neuropathic analgesia in the first instance and consider surgical evaluation if this does not work
E. Refer to physiology services and review in 6 weeks

Answer: A

Bilateral median nerve dysfunction is very suggestive of a diagnosis of degenerative cervical myelopathy (DCM) rather than bilateral carpal tunnel syndrome (option C). DCM should be suspected in elderly patients presenting with limb neurology. His twitches are probably fibrillations, a sign of lower motor neuron dysfunction.

Degenerative cervical myelopathy is associated with a delay in diagnosis, estimated to be >2 years in some studies [1]. It is most commonly misdiagnosed as carpal tunnel syndrome and in one study, 43% of patients who underwent surgery for degenerative cervical myelopathy, had been initially diagnosed with carpal tunnel syndrome [1]. Management of these patients should be by specialist spinal services (neurosurgery or orthopaedic spinal surgery). Decompressive surgery is the mainstay of treatment and has been shown to stop disease progression. Physiotherapy and analgesia does not replace surgical opinion, though they may be used alongside (options D and E). Nerve root injections do not have a role in management (option B).

1. Behrbalk E, Salame K, Regev GJ, Keynan O, Boszczyk B, Lidar Z. Delayed diagnosis of cervical spondylotic myelopathy by primary care physicians. Neurosurg Focus. 2013 Jul;35(1):E1.

5

Select the best management option for the following clinical scenarios. Each option can be chosen once, more than once or not at all.

A) Arrange X-rays of the cervical spine
B) Refer to pain clinic
C) Trial of analgesia and re-review
D) Refer to spinal surgery
E) Refer for physiotherapy
F) Lifestyle modification and co-morbidity optimisation
G) Trial of a cervical collar
H) Refer for a minor ops procedure, division of flexor retinaculum
I) Refer to neurology
J) Refer to elderly care medicine
K) Refer to vascular surgery

1. A 70 year-old male with a background of diabetes and hypertension presents with pain and weakness in both legs on walking. It settles with rest. His most recent HBA1c is 56 and an ankle-brachial pressure index is calculated as 0.7.
2. A 35 year-old female who is 30 weeks pregnant complains of right hand pain radiating into her thumb, index and middle finger. It often wakes her up from sleep.
3. A 65 year-old female presents with neck pain and loss of dexterity in both hands. She has been struggling to type at work and use her mobile phone. Her symptoms have been deteriorating gradually over the preceding months.

Answers

1 - F: this patient has peripheral vascular disease, as evident by his ankle-brachial pressure index (ABPI). NICE guidance suggests that first line management should include lifestyle modification such as smoking cessation, weight loss, lipid modification, optimisation of diabetes mellitus/hypertension and antiplatelet therapy. A supervised exercise programme can also be arranged. Local guidelines vary on when referral to specialist care is needed, but typically this would be where conservative treatment fails after 3 months or the ABPI is below a defined threshold (e.g. <0.6).

2 - E: this patient is likely to have carpal tunnel syndrome. This occurs due to median nerve entrapment beneath the flexor retinaculum. It is more common in pregnant women due to the increase in oedema. NICE clinical knowledge summaries (CKS) recommend lifestyle measures (e.g. wrist ergonomic devices at work), as well as wrist splints (usually prescribed by physiotherapists), corticosteroid injections or referral for surgical management. Wrist splints can be helpful for nighttime symptoms, as in her case. Corticosteroid injections require local expertise that may or may not be present.

3 - D: this patient is likely to have degenerative cervical myelopathy. DCM is often missed initially and there is a delay in the diagnosis of this condition by >2 years in some studies [1]. Patients have predominantly upper motor neuron signs such as increased toned, hyper-reflexia and pyramidal weakness. Neurological signs are often subtle initially and easily missed, but as a progressive condition they are likely to get worse [2]. Management of these patients should be by specialist spinal services

(neurosurgery or orthopaedic spinal surgery). An MRI scan is required for diagnosis. All patients should be assessed by a spinal surgeon.

References:

1. Behrbalk E, Salame K, Regev GJ, Keynan O, Boszczyk B, Lidar Z. Delayed diagnosis of cervical spondylotic myelopathy by primary care physicians. Neurosurg Focus. 2013 Jul;35(1):E1.
2. Baron EM, Young WF. Cervical spondylotic myelopathy: a brief review of its pathophysiology, clinical course, and diagnosis. Neurosurgery. 2007 Jan;60(1 Supp1 1):S35-41.

Select the best management option for the following clinical scenarios. Each option can be chosen once, more than once or not at all.

A) Arrange X-rays of the cervical spine
B) Refer to pain clinic
C) Trial of analgesia and re-review
D) Refer to spinal surgery
E) Refer for physiotherapy
F) Lifestyle modification and co-morbidity optimisation
G) Trial of a cervical collar
H) Refer for a minor ops procedure, division of flexor retinaculum
I) Refer to neurology
J) Refer to elderly care medicine
K) Refer to vascular surgery

1. A 60 year-old male presents with clumsy hands. He has been dropping cups around the house. His wife complains he doesn't answer his mobile as he struggles to use it. His symptoms have been gradually deteriorating over the preceding months.
2. A 32 year-old female presents with a 3 day history of altered sensation on her left foot and right forearm. She had an episode of visual blurring in her right eye a few months ago which resolved after a few days. Examination reveals brisk reflexes.
3. A 45 year-old female presents with stiffness and pain in her left shoulder, which started around a month ago. She had a similar episode that resolved by itself. Examination reveals global restriction of shoulder movement, particularly external rotation.

Answers

1 - D: this patient is likely to have degenerative cervical myelopathy. DCM is often missed initially and there is a delay in the diagnosis of this condition by >2 years in some studies [1]. Patients have predominantly upper motor neuron signs such as increased toned, hyper-reflexia and pyramidal weakness. Neurological signs are often subtle initially and easily missed, but as a progressive condition they are likely to get worse [2]. Management of these patients should be by specialist spinal services (neurosurgery or orthopaedic spinal surgery). An MRI scan is required for diagnosis. All patients should be assessed by a spinal surgeon.

2 - I: this patient is likely to have Multiple Sclerosis. Her visual loss was probably secondary to optic neuritis, a common ophthalmological association with multiple sclerosis. She should be referred to neurology.

3 - C: this patient is likely to have adhesive capsulitis (frozen shoulder). Patients have global restriction of shoulder movements, in at least two axes, though external rotation is classically described as the most affected and painful. Management of frozen shoulder is controversial and there is not much evidence to inform practice. In general, alternative diagnoses should be excluded and pain relief optimised. Gentle shoulder movement is encouraged and there is limited evidence for physiotherapy.

References:
1. Behrbalk E, Salame K, Regev GJ, Keynan O, Boszczyk B, Lidar Z. Delayed diagnosis of cervical spondylotic myelopathy by primary care physicians. Neurosurg Focus. 2013 Jul;35(1):E1.
2. Baron EM, Young WF. Cervical spondylotic myelopathy: a brief review of its pathophysiology, clinical course, and diagnosis. Neurosurgery. 2007 Jan;60(1 Supp1 1):S35-41.
