# Supplementary material for: Quantitative analysis of medical students’ and physicians’ knowledge of degenerative cervical myelopathy
Source: BMJ Open. 2020 Jan 12;10(1):e028455. doi: 10.1136/bmjopen-2018-028455 (PMC7044983; doi:10.1136/bmjopen-2018-028455)
Supplement: Supplementary data [file bmjopen-2018-028455supp002.pdf]

Supplementary Table 1. Trainee knowledge analysis. A = number of questions used in question-bank; B = number of correct responses / number of attempts; C = correct response rate (%). Note that each individual EMQ had 3 distinct clinical scenarios.

Theme	Type	Question-bank									Cumulative results by theme		
		Finals/PLAB (beginner)			SRA (intermediate)			MRCGP (advanced)					
		A	B	C	A	B	C	A	B	C	A	B	C
Presentation	MCQ	1	658/1718	38.0%	3	407/1280	31.8%	3	1303/4667	27.9%	7	2367/7665	30.9%
	EMQ	2	5665/7914	73.2%	2	12589/17208	73.2%	2	6747/9246	73.0%	6	29286/40056	73.1%
	Both	3	6323/9632	65.6%	5	12996/18488	70.2%	5	8050/13913	57.9%	13	31653/47721	66.3%
Assessment	MCQ	4	3801/5378	70.7%	5	630/1198	52.6%	5	3960/5837	67.8%	14	8391/12413	67.6%
	EMQ	1	1618/2952	54.8%	2	8360/14019	59.6%	2	4784/8988	53.2%	5	14762/25959	56.9%
	Both	5	5419/8330	65.1%	7	8990/15217	59.1%	7	8744/14825	59.0%	19	23153/38372	60.3%
Management	MCQ	5	4653/6750	68.9%	5	792/1324	59.8%	5	3850/5612	68.6%	15	9295/13686	67.9%
	EMQ	1	3224/6306	51.1%	2	6939/12501	55.6%	2	4806/8871	54.2%	5	14969/27678	54.1%
	Both	6	7877/13056	60.3%	7	7731/13825	55.9%	7	8656/14483	59.8%	20	24264/41364	58.7%
Cumulative results by question-bank	MCQ	10	9112/13846	65.8%	13	1828/3802	48.1%	13	9113/16116	56.5%	36	20053/33764	59.4%
	EMQ	4	14792/22860	64.7%	6	27888/43728	63.8%	6	16337/27105	60.3%	16	59017/93693	63.0%
	Both	14	23904/36706	65.1%	19	29716/47530	62.5%	19	25450/43221	58.9%	52	79070/127457	62.0%
