# Supplementary material for: Quantitative analysis of medical students’ and physicians’ knowledge of degenerative cervical myelopathy
Source: BMJ Open. 2020 Jan 12;10(1):e028455. doi: 10.1136/bmjopen-2018-028455 (PMC7044983; doi:10.1136/bmjopen-2018-028455)
Supplement: Author's manuscript [file bmjopen-2018-028455.draft_revisions.pdf]

BMJ Open is committed to open peer review. As part of this commitment we make the peer review history of every article we publish publicly available.

When an article is published we post the peer reviewers' comments and the authors' responses online. We also post the versions of the paper that were used during peer review. These are the versions that the peer review comments apply to.

The versions of the paper that follow are the versions that were submitted during the peer review process. They are not the versions of record or the final published versions. They should not be cited or distributed as the published version of this manuscript.

BMJ Open is an open access journal and the full, final, typeset and author-corrected version of record of the manuscript is available on our site with no access controls, subscription charges or pay-per-view fees (http://bmjopen.bmj.com).

If you have any questions on BMJ Open's open peer review process please email info.bmjopen@bmj.com

BMJ Open

A quantitative analysis of medical students' and physicians' knowledge of degenerative cervical myelopathy

Journal:	*BMJ Open*
Manuscript ID	bmjopen-2018-028455
Article Type:	Research
Date Submitted by the Author:	08-Dec-2018
Complete List of Authors:	Waqar, Mueez; University of Manchester, Academic Neurosurgery<br>Wilcock, Jane; University of Liverpool, Department of Undergraduate Medical Education<br>Garner, Jayne; University of Liverpool, Department of Undergraduate Medical Education<br>Davies, Benjamin; University of Cambridge, Department of Clinical Neurosurgery<br>Kotter, Mark; University of Cambridge, Department of Clinical Neurosurgery; Ann McLaren Laboratory of Regenerative Medicine, Department of Clinical Neurosciences
Keywords:	Cervical myelopathy, spondylosis, degenerative spine, physician knowledge, gap analysis

SCHOLARONE™
Manuscripts

A quantitative analysis of medical students' and physicians' knowledge of degenerative cervical myelopathy

Authors and affiliations

1. Mueez Waqar[1], mwaqar@doctors.org.uk
2. Jane Wilcock[2], Jane.Wilcock@liverpool.ac.uk
3. Jayne Garner[2], jayneg@liverpool.ac.uk
4. Benjamin M Davies[3,4], bd375@cam.ac.uk
5. Mark RN Kotter[3,4], mrk25@cam.ac.uk

[1]Department of Academic Neurosurgery, Manchester Academic Health Sciences Centre, The University of Manchester, Manchester, UK

[2]Department of Undergraduate Medical Education, The University of Liverpool, Liverpool, UK

[3]Department of Academic Neurosurgery, Cambridge University Hospital, University of Cambridge, Cambridge, UK

[4]WT MRC Cambridge Stem Cell Institute, Anne McLaren Laboratory, University of Cambridge, UK

Corresponding author

Dr. Mark R.N. Kotter

WT MRC Cambridge Stem Cell Institute and Department of Clinical Neurosciences

For peer review only - http://bmjopen.bmj.com/site/about/guidelines.xhtml

University of Cambridge

Acknowledgements

The authors would like to thank Passmedicine for their assistance with this work.

Declarations and conflicts of interest

Research in the senior author's laboratory is supported by a core support grant from the Wellcome Trust and MRC to the Wellcome Trust-Medical Research Council Cambridge Stem Cell Institute. MRNK is supported by a NIHR Clinician Scientist Award.

Disclaimer: This report is independent research arising from a Clinician Scientist Award, CS-2015-15-023, supported by the National Institute for Health Research. The views expressed in this publication are those of the authors and not necessarily those of the NHS, the National Institute for Health Research or the Department of Health.

Word count

2425

Data

Additional data is provided in supplementary table 1.

Author statement

Each author met ICMJE criteria for authorship:

- MW: coordinated creation of questions, performed statistical analysis, wrote up all manuscript drafts.
- JW: created first draft of questions for question-bank, aided in manuscript analysis and write-up, helped to check of final draft.

For peer review only - http://bmjopen.bmj.com/site/about/guidelines.xhtml

- JG: aided in creation of first draft of questions for question-bank, aided in manuscript analysis and checking of final draft.
- BMD: checked questions, oversaw data analysis, aided in authoring introduction and collecting key references, helped to devise project idea, checked final manuscript draft.
- MRNK: devised study idea, provided draft protocol, checked questions, checked final manuscript draft.

Funding

This research received no specific grant from any funding agency in the public, commercial or not-for-profit sectors

For peer review only - http://bmjopen.bmj.com/site/about/guidelines.xhtml

Abstract

Objectives. We have previously identified a delay in general practitioner (GP) referrals for patients with degenerative cervical myelopathy (DCM). The aim of this study was to evaluate whether an education gap existed for DCM along the GP training pathway by quantitatively assessing training in, and knowledge of, this condition.

Design. Gap analysis: comparison of DCM to other conditions. Comparators selected on the basis of similar presentation/epidemiology (multiple sclerosis), an important spinal emergency (cauda equina syndrome) and a common disease (diabetes mellitus).

Subjects. Medical students, foundation doctors and GP trainees.

Primary and secondary outcome measures. (1) Assessment of training: quantitative comparison of references to DCM in curricula (undergraduate/postgraduate) and commonly used textbooks (Oxford Handbook Series), to other conditions. (2) Assessment of knowledge: using standardised questions placed in an online question-bank (PassMedicine).

Results. DCM had the joint lowest modal rank of references to the condition in curricula analysis and 2nd lowest modal rank in textbook analysis. In knowledge analysis questions were attempted 127,457 times. The mean user performance decreased sequentially for DCM and other neuroscience subjects with advancing question-bank. However, performance for DCM questions in themes of presentation, workup and management were all greater than the question-bank mean and within one standard deviation. For students and junior trainees, there was a serial decrease in performance from presentation to workup (-0.7 to +10.4 relative to question-bank mean) and management (-0.6% to -3.9% relative to question-bank mean).

For peer review only - http://bmjopen.bmj.com/site/about/guidelines.xhtml

Conclusions. Although infrequently cited in curricula and learning resources, knowledge relating to DCM was above average. However, knowledge relating to its management was relatively poor.

Keywords

Cervical myelopathy; spondylosis; degenerative spine; physician knowledge; gap analysis

Strengths and limitations of this study

- Search terms relating to DCM were queried from three UK specific medical school curricula and relevant postgraduate curricula
- Only a handful of learning resources were searched to assess references to DCM
- A large number of responses were obtained by placing questions in an online question-bank, relating to DCM

For peer review only - http://bmjopen.bmj.com/site/about/guidelines.xhtml

Introduction

Degenerative cervical myelopathy (DCM) is a common and insidious condition that can lead to severe disability.[1] It arises when degenerative changes of the spine compress the spinal cord, causing a progressive spinal cord injury. Currently, treatment is limited to surgical decompression, which is able to stop further injury but due to the limited capacity of the spinal cord to repair, recovery is limited. The timing of surgery is therefore crucial to recovery ('time is spine'), and a recent meta-analysis has demonstrated treatment within 6 months offers a greater chance of making a full recovery.[2] Unfortunately, few patients are diagnosed promptly, with the majority waiting more than 2 years for a diagnosis.[3] Consequently, most patients retain life-long disabilities, contributing to quality of life scores lower than cancer, heart and lung diseases.[4, 5]

The diagnostic pathway for DCM almost exclusively starts with assessment and triage by a community physician, termed in the UK a General Practitioner (GP).[3] If appropriate, the patient is then referred on for further investigation and management. Our analysis of this pathway has identified time to initial referral by GP (6.4±7.7) as representing 51% of diagnostic delay.[6]

This period of the diagnostic pathway is difficult to examine in detail, and whilst delayed patient presentation is likely to contribute, delayed detection measured by multiple consultations and patient perspective, is certainly a significant component.[3,

For peer review only - http://bmjopen.bmj.com/site/about/guidelines.xhtml

7] The cause for delayed detection is also likely to be multi-faceted, including subtle, non-specific symptoms and incomplete clinical examination.[1] However, it is conceivable that a lack of understanding of the workup and management of DCM may also contribute. Indeed, DCM would be included within the spectrum of "neurophobia" – an aversion to the neurosciences due to perceived difficulty, which has been demonstrated in GPs and GP trainees.[8, 9]

Our objective therefore was to evaluate whether an education gap exists for DCM amongst GPs, by quantitatively assessing their training in, and knowledge of, DCM.

Methods

Ethics

The present study design did not require ethical approval.

Education Gap Analysis

A gap analysis is a process to identify gaps in existing systems such as education curricula. It involves an assessment of existing knowledge or standards against pre-determined standards that define core competency requirements.[10, 11] This differs, for example, from simple cross sectional knowledge assessments whose primary outcome is usually assessment of knowledge beyond core requirements.

For peer review only - http://bmjopen.bmj.com/site/about/guidelines.xhtml

The study objective was therefore approached in two separate gap analyses (Table 1):

1. **Assessment of Training**: quantification of DCM in curricula and commonly used learning resources, including assessment of relative importance through comparison to other conditions.
2. **Assessment of Knowledge**: formal assessment of trainees' knowledge using questions placed in an online question-bank.

The GP Training Pathway

UK medical graduates complete the UK Foundation Programme to enter higher specialty training as formal GP trainees. There are several assessments along this route including: medical school final examinations to gain registration to practice in the UK, or the equivalent Professional and Linguistic Assessments Board test (PLAB) for international medical graduates; the Specialty Recruitment Assessment (SRA) – a written assessment taken towards the start of the second foundation year prior to higher specialty applications; and the Membership of The Royal College of General Practitioner's (MRCGP), an exit exam for GP trainees. Whilst alternative entry routes to General Practice exist, this pathway represents the most common training route for GPs today.

Definition of DCM and comparators

For peer review only - http://bmjopen.bmj.com/site/about/guidelines.xhtml

DCM was chosen as an inclusive term for a variety of diseases resulting in compressive myelopathy.[1] Comparator diseases were also selected to compare findings within and outside the field of DCM:

1. **Within Myelopathy:** a differential for DCM with equivalent or greater incidence.
2. **Within Degenerative Spine**: an alternative degenerative spine disease that is widely taught.
3. **Outside Neurology**: a common disease that all clinicians would have some knowledge about and interaction with.

The *a priori* hypothesis was, if an education gap existed within DCM, we would expect metrics of training and knowledge of DCM to rank inferior to controls. Controls were identified through a consensus author meeting and finalised after confirmation of epidemiological profiles through literature review.

Assessment of Training: Learning Resource Analysis

We selected learning resources that were in common usage by trainees, available in electronic format (to be amenable for searching) and able to be stratified by training stage. Specifically, training curricula, the Oxford Handbook series [12] and online question-banks were selected. Alongside the United Kingdom Foundation Programme and MRCGP, an example from each of the three main, UK medical school teaching models [13] were included: problem-based learning (The University of Manchester,

For peer review only - http://bmjopen.bmj.com/site/about/guidelines.xhtml

UK); traditional lecture based (The University of Cambridge, UK); and an integrated system including aspects of both (Imperial College London, UK). The choice of medical school curricula was pragmatically selected, based on access. The handbooks most pertinent to the GP training pathway were included in our analysis: The Oxford Handbook of Clinical Medicine (OHCM), The Oxford Handbook for the Foundation Programme (OHFP) and The Oxford Handbook of General Practice (OHGP).

Search terms for analysing learning resources were created for DCM and each comparator using relevant terms from the search syntax of Cochrane Reviews, or if absent, recent systematic reviews. Curricula were searched for the number of references per disease, text books for the number of words per disease section and question banks for the number of questions.

Assessment of Knowledge

The author panel includes GPs with an interest in education (JW), university appointed educationalists (JG) and neurosurgeons (MW, BMD, MRK). The authors devised a set of questions to be included in an online question bank, composed of both multiple choice questions [MCQs] and extended matching questions [EMQs], designed to cover the different components of medical assessment; presentation, investigation and management. The final set consisted of 19 questions (13 multiple choice

For peer review only - http://bmjopen.bmj.com/site/about/guidelines.xhtml

questions [MCQs] and 6 extended matching questions [EMQs]). The questions together with answers are included in Supplement 1.

Online question-banks including subsections devoted to each stage of the GP training pathway were contacted with details of the study. Only one question-bank - Passmedicine [14], responded and was therefore selected for this arm of the study.

Analysis

Statistical analysis was performed using SPSS version 22 (SPSS Inc., Chicago, Illinois, USA). For assessment of training, frequencies of search terms were described and the mode and modal ranks determined. For assessment of knowledge, histograms were constructed with either a condition or question-bank along the X-axis and user performance relative to question-bank mean along the Y-axis. The user performance relative to question-bank mean corresponded to the raw difference between the mean value for each theme and the mean value for a particular question-bank.

For peer review only - http://bmjopen.bmj.com/site/about/guidelines.xhtml

Results

Selection of Comparators and Resources

Multiple sclerosis (MS), cauda equina syndrome (CES) and diabetes mellitus were selected as the three disease comparators and list of search terms compiled accordingly (Table 2).[15-18] MS is a common differential for DCM,[19] with overlapping signs and symptoms. Whilst DCM is likely to be more prevalent in reality,[1] currently their characterised epidemiology is comparable.[20, 21] CES is uncommon, but its missed or delayed diagnosis carries significant consequences. Diabetes is relevant to all medical fields.

Assessment of training

Curricula analysis

DCM and cauda equina syndrome had the lowest modal rank in curricula search analysis (Table 3). This was true for early stage undergraduate curricula and late stage curricula for the foundation programme and MRCGP. Multiple sclerosis was mentioned less frequently in early versus late stage curricula. The opposite trend was observed for diabetes mellitus.

Textbook analysis

For peer review only - http://bmjopen.bmj.com/site/about/guidelines.xhtml

Overall, DCM had the second lowest modal rank but above that of cauda equina syndrome (Table 4). The relative word count attributed to neurological conditions such as DCM, multiple sclerosis and cauda equina syndrome decreased with advancing stage of textbook. The opposite trend was observed for diabetes mellitus, for whom the total word count seemed to increase.

For peer review only - http://bmjopen.bmj.com/site/about/guidelines.xhtml

Question Bank Analysis

Assessment of Knowledge

Passmedicine was approached, on the basis it did not have any DCM questions previously which could influence analysis. Questions were introduced between the period June 2017 – October 2017. Section editors for each question-bank ultimately selected which of the 19 questions were relevant, such that finals/PLAB contained 14, SRA – 19 and MRCGP – 19.

Overall, questions were attempted 127,457 times; finals/PLAB - 36706; SRA - 47530; MRCGP – 43221 (Supplementary Table 1). Data on the number of unique attempts or first-time viewers was not extractable.

There were differences in user performance in the three DCM question themes – presentation, workup and management, though average scores were all within 1 standard deviation of the mean (Fig. 1). Performance sequentially decreased across these themes for the finals/PLAB group, SRA group and when all groups were considered together. In the MRCGP group however, performance clustered around the mean for the question bank with little variability.

User performance by disease showed large variation between question-banks (Fig. 2). The mean user performance decreased sequentially for DCM and other

For peer review only - http://bmjopen.bmj.com/site/about/guidelines.xhtml

neuroscience subjects with advancing question-bank. However, the mean user performance for DCM was always greater than the question-bank mean.

Discussion

In this study, we found that DCM is relatively infrequently cited in curricula and commonly used textbooks, even compared to diseases such as multiple sclerosis, with similar epidemiology. Despite this however, user performance in DCM questions remained consistently above question-bank averages, unlike other neuroscience themes such as multiple sclerosis and cauda equina syndrome. There was a sequential decrease in user performance across the themes of DCM presentation, workup and management for early years' trainees, whereas for senior trainees, performance did not vary by theme of question. The user performance was either consistently below average or decreased sequentially for DCM and other neuroscience themes such as multiple sclerosis and cauda equina syndrome. This was not observed for diabetes mellitus.

Although the present study design is novel, many of our findings correlate to previous reports. Firstly, there is the concept of "neurophobia", a term given to the relatively poor performance of neurosciences subjects relative to others. The present study adds to this literature surrounding neurophobia, which is well described for GPs and GP trainees and students.[8, 9, 22] In one questionnaire study for example, GPs rated neurology as the most difficulty medical specialty and the one for which they had the

For peer review only - http://bmjopen.bmj.com/site/about/guidelines.xhtml

least confidence compared to cardiology, endocrinology, gastroenterology, geriatrics, respiratory medicine and rheumatology.[9] DCM was unusual in that performance in this particular theme remained consistently above average, with a sequential decrease with increasing training stage. This correlated with decreasing emphasis in more advanced curricula.

Secondly, our study supports the observation of poorer performance in management themes by clinicians at an earlier training stage. Prior studies on medical students that have evaluated knowledge formally have shown similar results in specialties such as ophthalmology but not DCM.[23] This also appears to translate into patient encounters during clinical assessments in neurology.[24, 25] In one study evaluating student performance in neurology outpatients, students were more likely to make errors regarding diagnostic tests or planning treatment than lesion localisation.[24] Another study evaluating student performance in neurology themed objective structured clinical examinations (OSCEs) also reported similar results, finding poorer student performance in supportive management of neurological conditions versus diagnosis.[25]

Our original hypothesis attempting to correlate delays in diagnosis for DCM with a deficiency in training and knowledge, was not conclusively demonstrated in the present study. Earlier stage trainee doctors demonstrated comparably higher levels of knowledge with regards to the presentation and work up of DCM and a potential gap

For peer review only - http://bmjopen.bmj.com/site/about/guidelines.xhtml

with regards to management-related questions. These differences disappeared in individuals studying for the GP exit exams. Nevertheless, the clinical argument for an education gap is strong - our previous analysis of the referral pathway for DCM identified a time to initial referral by the GP as representing 51% of the overall diagnostic delay.

This study demonstrated DCM received relatively less training than other conditions. In the West, the prevalence of DCM is underestimated but at least 60 per 100,000, compared to around 100 per 100,000 for multiple sclerosis.[20, 21] Despite this however, DCM was under-represented by eight-fold compared to multiple sclerosis in curricula analysis. In late stage curricula (foundation programme, MRCGP), DCM was not referred to at all. A similar trend was observed in textbook analysis, though neither of these analyses were exhaustive. These results were in contrast to our knowledge analysis, which showed consistently above average performance for DCM unlike multiple sclerosis.

One potential way of reconciling the absence of a knowledge gap with the deficiencies in the diagnosis and management of DCM, is that factual knowledge may not translate into recognition of the disease in the clinical context. Causal association between training and clinical practice have not been clearly demonstrated, albeit prior studies have found good correlation between exam performance and clinical performance in a variety of medical and surgical specialties.[26-28] Most of this data is based on the

For peer review only - http://bmjopen.bmj.com/site/about/guidelines.xhtml

United States Medical Licensing Examination (USMLE), which appears to correlate well with residency performance.[26, 27] Beyond medical school exams, in another study, internal medicine clinicians failing their maintenance of certification exams had a more than double chance of disciplinary action versus those who passed.[28]

There are several limitations to be considered in the context of these findings. Firstly, only select learning resources were used in this study and questions were inserted into a single online question bank. Secondly, question bank data was not extractable per user, nor per first time answer. Whilst this was not a comprehensive strategy, this is unlikely to have limited our results for at least two reasons: questions were attempted more than 100,000 times and analysis was relative within learning resources, and therefore limitations were applicable to each comparator.

The methodology employed in the present study may not have been sufficiently sensitive to detect a knowledge gap for DCM, for several reasons. Firstly, our questions may not have been challenging enough given that they tested core principles all clinicians would be expected to achieve. Secondly, there was no first-time answer data available for analysis. Thirdly, our controls may not have been appropriate and certainly, educational comparisons between specialties is not always the best method of assessing knowledge adequacy.[29] Finally, our methodology assumed a direct correlation between “on paper” knowledge and clinical care provided to patients – though this was not proven in the present study and could still differ vastly.

For peer review only - http://bmjopen.bmj.com/site/about/guidelines.xhtml

Conclusion

In this study, we set out to evaluate whether there is a deficiency in training and knowledge regarding DCM in the GP training pathway. Although DCM was infrequently referenced in learning resources, trainees performed above average for DCM questions in an assessment of knowledge using an online question-bank. The clinical suggestion of an education gap is strong, and these contrasting findings do not conclusively allay this concern. Future studies are required to better understand these observations.

References

1. Davies BM, Mowforth OD, Smith EK, et al. Degenerative cervical myelopathy. *BMJ* 2018;360:k186.

2. Fehlings MG, Tetreault LA, Riew KD, et al. A Clinical Practice Guideline for the Management of Patients With Degenerative Cervical Myelopathy: Recommendations for Patients With Mild, Moderate, and Severe Disease and Nonmyelopathic Patients With Evidence of Cord Compression. *Global spine journal* 2017;7(3 Suppl):70S-83S.

3. Behrbalk E, Salame K, Regev GJ, et al. Delayed diagnosis of cervical spondylotic myelopathy by primary care physicians. *Neurosurgical focus* 2013;35(1):E1.

4. Fehlings MG, Ibrahim A, Tetreault L, et al. A global perspective on the outcomes of surgical decompression in patients with cervical spondylotic myelopathy: results from

For peer review only - http://bmjopen.bmj.com/site/about/guidelines.xhtml

the prospective multicenter AOSpine international study on 479 patients. *Spine* 2015;40(17):1322-8.

5. Oh T, Lafage R, Lafage V, et al. Comparing Quality of Life in Cervical Spondylotic Myelopathy with Other Chronic Debilitating Diseases Using the Short Form Survey 36-Health Survey. *World Neurosurg* 2017;106:699-706.

6. Hilton B T-MJ, Davies BM, Kotter MRN. Route to diagnosis of degenerative cervical myelopathy in a UK healthcare system: a retrospective cohort study. *BMJ Open (in-press)* 2018

7. Elemental Ideas – Cervical Spondylotic Myelopathy Part One [cited 2018 October 12th]. Available from: http://archive.cambridge-tv.co.uk/elemental-ideas-cervical-spondylotic-myelopathy-part-one/ accessed October 12th 2018.

8. McCarron MO, Stevenson M, Loftus AM, et al. Neurophobia among general practice trainees: the evidence, perceived causes and solutions. *Clinical neurology and neurosurgery* 2014;122:124-8.

9. Loftus AM, Wade C, McCarron MO. Primary care perceptions of neurology and neurology services. *Postgraduate medical journal* 2016;92(1088):318-21.

10. Fater KH. Gap analysis: a method to assess core competency development in the curriculum. *Nurs Educ Perspect* 2013;34(2):101-5.

11. O'Brien CE, Stafford R, Franks AM. Establishment of a patient-centered communication course to address curricular gaps. *Curr Pharm Teach Learn* 2018;10(7):933-39.

For peer review only - http://bmjopen.bmj.com/site/about/guidelines.xhtml

12. Warriner D. The Oxford Handbook of Clinical Medicine. *BMJ* 2008;336(7640):393-93.

13. NHS careers. Teaching styles at medical school [27/05/2018]. Available from: https://www.healthcareers.nhs.uk/sites/default/files/documents/Teaching%20styles%20at%20medical%20school.pdf accessed 27/05/2018.

14. Passmedicine 2018 [cited 2018 12th October]. Available from: https://www.passmedicine.com accessed 12 October 2018.

15. Kiely PD, Quinn JC, Du JY, et al. Posterior surgical treatment of cervical spondylotic myelopathy: review article. *HSS J* 2015;11(1):36-42.

16. Khan F, Amatya B. Rehabilitation in Multiple Sclerosis: A Systematic Review of Systematic Reviews. *Arch Phys Med Rehabil* 2017;98(2):353-67.

17. Chau AM, Xu LL, Pelzer NR, et al. Timing of surgical intervention in cauda equina syndrome: a systematic critical review. *World Neurosurg* 2014;81(3-4):640-50.

18. Richter B, Bandeira-Echtler E, Bergerhoff K, et al. Pioglitazone for type 2 diabetes mellitus. *Cochrane Database Syst Rev* 2006(4):CD006060.

19. Davies BM, Munro CF, Kotter M. A Novel Insight into the Challenges of Diagnosing Degenerative Cervical Myelopathy using Online Symptom Checkers. *J Med Internet Res* 2018

20. Nouri A, Tetreault L, Singh A, et al. Degenerative Cervical Myelopathy: Epidemiology, Genetics, and Pathogenesis. *Spine* 2015;40(12):E675-93.

21. Dilokthornsakul P, Valuck RJ, Nair KV, et al. Multiple sclerosis prevalence in the United States commercially insured population. *Neurology* 2016;86(11):1014-21.

For peer review only - http://bmjopen.bmj.com/site/about/guidelines.xhtml

22. Flanagan E, Walsh C, Tubridy N. 'Neurophobia'--attitudes of medical students and doctors in Ireland to neurological teaching. *Eur J Neurol* 2007;14(10):1109-12.

23. Esparaz ES, Binder SB, Borges NJ. How prepared are medical students to diagnose and manage common ocular conditions. *J Educ Eval Health Prof* 2014;11:29.

24. Davis LE, King MK. Assessment of medical student clinical competencies in the neurology clinic. *Neurology* 2007;68(8):597-9.

25. Lukas RV, Blood A, Park YS, et al. Assessment of neurological clinical management reasoning in medical students. *Journal of clinical neuroscience : official journal of the Neurosurgical Society of Australasia* 2014;21(6):919-22.

26. Thundiyil JG, Modica RF, Silvestri S, et al. Do United States Medical Licensing Examination (USMLE) scores predict in-training test performance for emergency medicine residents? *J Emerg Med* 2010;38(1):65-9.

27. Sutton E, Richardson JD, Ziegler C, et al. Is USMLE Step 1 score a valid predictor of success in surgical residency? *Am J Surg* 2014;208(6):1029-34; discussion 34.

28. McDonald FS, Duhigg LM, Arnold GK, et al. The American Board of Internal Medicine Maintenance of Certification Examination and State Medical Board Disciplinary Actions: a Population Cohort Study. *J Gen Intern Med* 2018

29. Di Paola M, Bennet GC, Shearer JR. Orthopaedic teaching in United Kingdom medical schools. *Med Teach* 1986;8(2):155-8.

For peer review only - http://bmjopen.bmj.com/site/about/guidelines.xhtml

Figure captions

Figure 1. Trainee knowledge analysis: user performance by DCM question theme. DCM questions assessed three themes - presentation, workup and management. Early stage trainees, those sitting finals/PLAB and the SRA, performed worse in questions relating to management versus more senior trainees taking the MRCGP. Performance for MRCGP trainees was more consistent. The average performance for each question theme was within 1 standard deviation (SD) of the mean.

Figure 2. Trainee knowledge analysis: user performance by disease. The mean user performance for DCM, neurology and cauda equina syndrome decreased with advancing question-bank. This trend was observed in most neurological pathologies. User performance of diabetes mellitus was more variable. * = there were no questions on cauda equina syndrome present in the SRA question-bank.

For peer review only - http://bmjopen.bmj.com/site/about/guidelines.xhtml

Table 1. Summary of gap analysis methods.

	Medical School	Foundation	GP Training	Metric
Curricula	UoM<br>UoC<br>Imperial	Foundation programme curriculum	MRCGP curriculum	References to search terms
Text Book	OHCM	OHFP	OHGP	References to search terms
Online Question Bank	PLAB<br>Medical finals	SRA	AKT	Performance in questions

For peer review only - http://bmjopen.bmj.com/site/about/guidelines.xhtml

Table 2. Selected diseases for textbook and curricula searches. The reasoning for each of the comparators is explained in this table. The search terms employed for the curricula searches are shown below this.

	Degenerative cervical myelopathy	Multiple sclerosis	Cauda equina syndrome	Diabetes mellitus
Reasoning	Disease of interest	A disease with a similar incidence and morbidity to degenerative cervical myelopathy. Selected as a clinico-epidemiological control [19]	Widely taught spinal emergency, selected as a spinal control.	Non-spinal control
Search terms for curricula searches	• Cervical myelopathy<br>• Cervical myelopathy<br>• Cervical myeloradiculopathy<br>• Cervical stenosis<br>• Cervical compression<br>• Cervical herniation<br>• Cervical degeneration<br>• Ossification of posterior longitudinal ligament<br>• Spinal osteophytosis<br>• Spinal cord compression<br>• Spondylosis	• Multiple sclerosis<br>• Demyelinating disease<br>• Demyelination	• Cauda equina<br>• Saddle anaesthesia	• Diabetes mellitus<br>• Insulin dependent diabetes mellitus<br>• Type 1 diabetes mellitus<br>• Non insulin dependent diabetes mellitus<br>• Type 2 diabetes mellitus<br>• Maturity onset diabetes of young<br>• Gestational diabetes mellitus<br>• Late onset diabetes<br>• Maturity onset diabetes<br>• Insulin resistance<br>• Diabetic ketoacidosis<br>• Hyperosmolar hyperglycaemic state<br>• Hyperosmolar non ketotic coma

For peer review only - http://bmjopen.bmj.com/site/about/guidelines.xhtml

Table 3. Curricula analysis. Electronic copies of curricula were queried with relevant search terms.

Curriculum	Number of references to term			
	Degenerative cervical myelopathy	Multiple sclerosis	Cauda equina syndrome	Diabetes mellitus
Undergraduate - PBL	0	3	1	43
Undergraduate – traditional	1	13	1	66
Undergraduate – integrated	3	15	0	30
Foundation Programme	0	0	0	0
MRCGP	0	4	2	113
**Total**	**4**	**32**	**4**	**252**
**Rank**	**3**	**2**	**3**	**1**

For peer review only - http://bmjopen.bmj.com/site/about/guidelines.xhtml

Table 4. Learning resource analysis. The number of words devoted to DCM and other diseases were determined. Importantly, only the words contained within the main section for the particular disease were considered.

Resource	Word count devoted to			
	Degenerative cervical myelopathy	Multiple sclerosis	Cauda equina syndrome	Diabetes mellitus
OHCM	870	1104	567	5165
OHFP	0	112	58	3599
OHGP	252	679	120	5736
Cumulative	1122	1895	745	14500
Modal rank	3	2	4	1

For peer review only - http://bmjopen.bmj.com/site/about/guidelines.xhtml

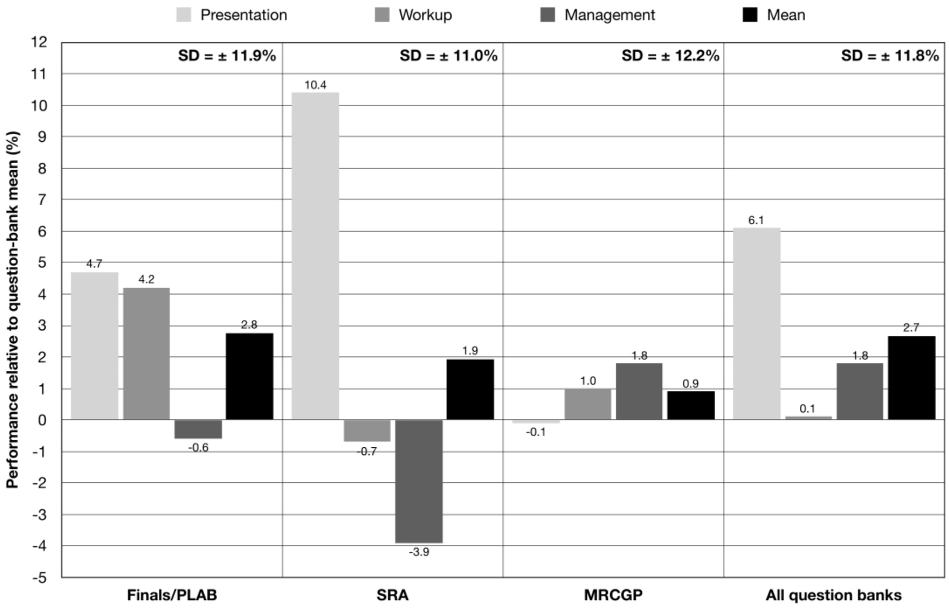

Figure 1

364x239mm (300 x 300 DPI)

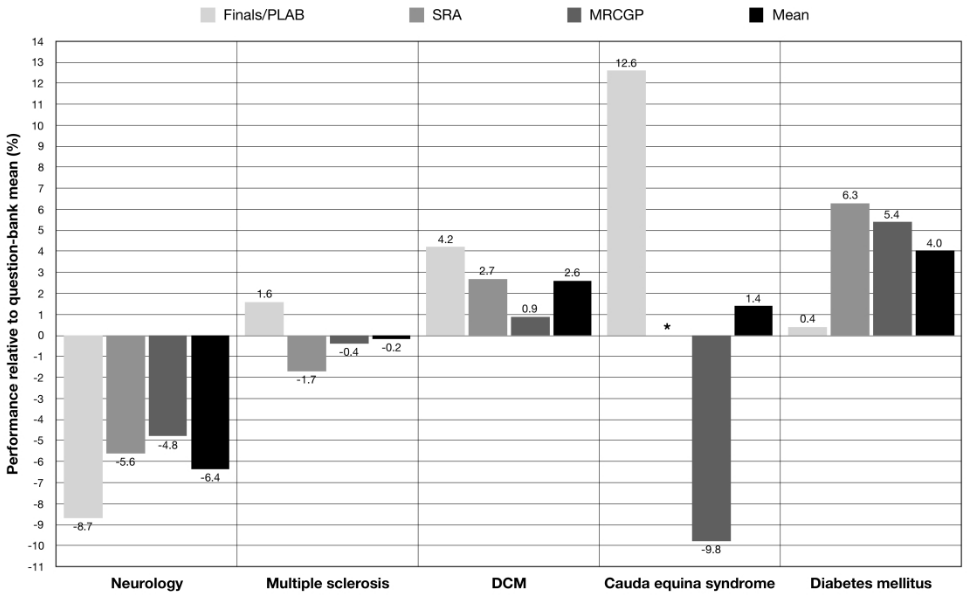

Figure 2

381x236mm (300 x 300 DPI)

Supplementary Table 1. Trainee knowledge analysis. A = number of questions used in question-bank; B = number of correct responses / number of attempts; C = correct response rate (%). Note that each individual EMQ had 3 distinct clinical scenarios.

Theme	Type	Question-bank									Cumulative results by theme		
		Finals/PLAB (beginner)			SRA (intermediate)			MRCGP (advanced)					
		A	B	C	A	B	C	A	B	C	A	B	C
Presentation	MCQ	1	658/1718	38.0%	3	407/1280	31.8%	3	1303/4667	27.9%	7	2367/7665	30.9%
	EMQ	2	5665/7914	73.2%	2	12589/17208	73.2%	2	6747/9246	73.0%	6	29286/40056	73.1%
	Both	3	6323/9632	65.6%	5	12996/18488	70.2%	5	8050/13913	57.9%	13	31653/47721	66.3%
Assessment	MCQ	4	3801/5378	70.7%	5	630/1198	52.6%	5	3960/5837	67.8%	14	8391/12413	67.6%
	EMQ	1	1618/2952	54.8%	2	8360/14019	59.6%	2	4784/8988	53.2%	5	14762/25959	56.9%
	Both	5	5419/8330	65.1%	7	8990/15217	59.1%	7	8744/14825	59.0%	19	23153/38372	60.3%
Management	MCQ	5	4653/6750	68.9%	5	792/1324	59.8%	5	3850/5612	68.6%	15	9295/13686	67.9%
	EMQ	1	3224/6306	51.1%	2	6939/12501	55.6%	2	4806/8871	54.2%	5	14969/27678	54.1%
	Both	6	7877/13056	60.3%	7	7731/13825	55.9%	7	8656/14483	59.8%	20	24264/41364	58.7%
Cumulative results by question-bank	MCQ	10	9112/13846	65.8%	13	1828/3802	48.1%	13	9113/16116	56.5%	36	20053/33764	59.4%
	EMQ	4	14792/22860	64.7%	6	27888/43728	63.8%	6	16337/27105	60.3%	16	59017/93693	63.0%
	Both	14	23904/36706	65.1%	19	29716/47530	62.5%	19	25450/43221	58.9%	52	79070/127457	62.0%

For peer review only

Passmedicine questions

Authors

1. Mueez Waqar
2. Jane Wilcock
3. Jayne Garner
4. Benjamin M Davies
5. Mark RN Kotter

Introduction

The following is a set of questions prepared for the degenerative cervical myelopathy education initiative. We would request a link to our website - www.myelopathy.org, our website to promote this initiative, below these questions.

Methods

Questions have been prepared as follows:

- Clinical presentation - focussing on differential diagnosis: 5 MCQs, 2 EMQs
- Assessment - focussing on examination and workup: 5 MCQs, 2 EMQs
- Management - focussing on referral pathways and follow-up: 5 MCQs, 2 EMQs

These questions are targeted to the following question banks:

- MRCGP
- MSRA
- Medical finals

Output

The aim of this initiative is to provide data on clinician knowledge on degenerative cervical myelopathy. We would like to analyse the following metrics based on these questions:

- Distribution of answers on 1st attempt
- Distribution of answers on 2nd attempt

In addition, we would like to compare the correct response rate of this sample to existing questions in the question-bank along the following themes:

- Multiple sclerosis
- Cauda equina syndrome
- Diabetes mellitus

For peer review only

Theme 1: Clinical presentation

A 65-year-old female reports progressive unsteadiness, mild weakness, pain and paraesthesias in her legs over the last 2-3 months. She reports no symptoms in her upper limbs and there is no history of trauma. Examination reveals increased lower limb tone with brisk reflexes. Which of the following is the most likely explanation for her symptoms?

A. Motor neuron disease
B. Degenerative cervical myelopathy
C. Multiple sclerosis
D. Syringomyelia
E. Cauda equina syndrome

Answer: B

Degenerative cervical myelopathy (DCM) refers to cervical spinal cord compression due to cervical spondylosis. The term replaces CSM (Cervical Spondylotic Myelopathy). Patients with DCM can present with predominantly lower limb symptoms. Examination findings, especially early on, are often subtle. DCM is the most common cause of non-traumatic paraparesis in developed countries and becomes more common with age [2]; it is estimated to have a prevalence of 5% in over 50-year-olds [3, 4]. In a series of patients presenting to Neurology outpatients, it was twice as common as Multiple Sclerosis [2]. Early diagnosis is key to ensuring good clinical outcomes. Currently most patients wait over 2 years for a diagnosis.

Incorrect options:

- Motor neuron disease is a disease of motor neurons, with mixed upper and lower motor features. Patients are classically described to have brisk reflexes in wasted and fasciculating limbs. As a rule, motor symptoms of weakness predominate and patients do not have sensory findings.
- Multiple sclerosis can also cause paraparesis. As a disease of the central nervous system, the paraparesis is usually associated with upper motor neuron signs. A first presentation of multiple sclerosis is most common between the ages of 20 and 40, younger than this patient. Degenerative Cervical Myelopathy is also more common with age and more common than MS overall, making that diagnosis more likely.

For peer review only

- Syringomyelia refers to the development of a syrinx in the spinal cord. It presents with a central cord syndrome, with predominantly upper limb signs. It is a relatively uncommon condition.
- Cauda equina syndrome results from compression of the cauda equina and classically includes leg weakness, saddle anaesthesia and sphincter disturbance. It is usually an acute syndrome with progressive signs. It is also a relatively uncommon condition.

References:

1. Kjaer P, Leboeuf-Yde C, Korsholm L, Sorensen JS, Bendix T. Magnetic resonance imaging and low back pain in adults: a diagnostic imaging study of 40-year-old men and women. Spine. 2005 May 15;30(10):1173–80.
2. Moore AP, Blumhardt LD. A prospective survey of the causes of non-traumatic spastic paraparesis and tetraparesis in 585 patients. Spinal Cord. 1997 Jun;35(6):361-7.
3. Kovalova I, Kerkovsky M, Kadanka Z, Kadanka Z, Nemec M, Jurova B, et al. Prevalence and Imaging Characteristics of Non-Myelopathic and Myelopathic Spondylotic Cervical Cord Compression. Spine. 2016 Aug 9.
4. Bednarik J, Kadanka Z, Dusek L, Kerkovsky M, Vohanka S, Novotny O, et al. Presymptomatic spondylotic cervical myelopathy: an updated predictive model. Eur Spine J. 2008 Mar;17(3):421–31.

For peer review only

A 73-year-old male presents with progressively worsening gait and urinary urgency. He is diagnosed with degenerative cervical myelopathy. Which ONE of the following is true regarding this condition?

A. Smoking is not a risk factor in isolation
B. Asians can have a different underlying aetiology than caucasians
C. Bowel and bladders symptoms are rare and should prompt consideration of cauda equina syndrome
D. Most patients present with a classic triad of neck pain, finger paraesthesias and weak legs
E. Family history is of limited value

Answer: B

Asian populations have a higher rate of ossification of the posterior longitudinal ligament (OPLL), which can result in myelopathy.

Degenerative cervical myelopathy (DCM) has a number of risk factors, which include smoking due to its effects on the intervertebral discs (A, false), genetics (option E, false) and occupation - those exposing patients to high axial loading [1].

The presentation of DCM is very variable (option D, false). Early symptoms are often subtle and can vary in severity day to day, making the disease difficult to detect initially. However as a progressive condition, worsening, deteriorating or new symptoms should be a warning sign.

DCM symptoms can include any combination of [1]:

- Pain (affecting the neck, upper or lower limbs)
- Loss of motor function (loss of digital dexterity, preventing simple tasks such as holding a fork or doing up their shirt buttons, arm or leg weakness/stiffness leading to impaired gait and imbalance
- Loss of sensory function causing numbness
- Loss of autonomic function (urinary or faecal incontinence and/or impotence) - these can occur and do not necessarily suggest cauda equina syndrome in the absence of other hallmarks of that condition

For peer review only

The most common symptoms at presentation of DCM are unknown, but in one series 50% of patients were initially incorrectly diagnosed and sometimes treated for carpal tunnel syndrome [2].

References

1. Baron EM, Young WF. Cervical spondylotic myelopathy: a brief review of its pathophysiology, clinical course, and diagnosis. Neurosurgery. 2007 Jan;60(1 Supp1 1):S35-41.

2. Behrbalk E, Salame K, Regev GJ, Keynan O, Boszczyk B, Lidar Z. Delayed diagnosis of cervical spondylotic myelopathy by primary care physicians. Neurosurg Focus. 2013 Jul;35(1):E1.

For peer review only

Stem 1: A 60-year-old gentleman with a background of hypertension and high cholesterol presents with a long history of neck pain, lower limb stiffness and urinary hesitancy. Which of the following is most likely?

Stem 2: A 56-year-old gentleman presents with lower limb stiffness and imbalance. His only past medical history of note is carpal tunnel syndrome that was diagnosed a year ago on clinical grounds and has been refractory to treatment with splints and steroid injections. Which of the following is most likely?

A. Cauda equina syndrome
B. Subacute combined degeneration of the cord
C. Degenerative cervical myelopathy
D. Parkinson's disease
E. Multiple sclerosis

Answer: C

The presentation of degenerative cervical myelopathy [DCM] is variable. Early symptoms are often subtle and can vary in severity day to day, making the disease difficult to detect initially. However as a progressive condition, worsening, deteriorating or new symptoms should be a warning sign.

DCM symptoms can include any combination of [1]:

- Pain (affecting the neck, upper or lower limbs)
- Loss of motor function (loss of digital dexterity, preventing simple tasks such as holding a fork or doing up their shirt buttons, arm or leg weakness/stiffness leading to impaired gait, imbalance and
- Loss of sensory function causing numbness
- Loss of autonomic function (urinary or faecal incontinence and/or impotence).

The most common symptoms at presentation of DCM are unknown, but in one series 50% of patients were initially incorrectly diagnosed and sometimes treated for carpal tunnel syndrome [2].

Other answers:

- Cauda equina syndrome results from compression of the cauda equina and classically includes leg weakness, saddle anaesthesia and sphincter disturbance.

For peer review only

It is usually an acute syndrome with progressive signs. It does not cause leg stiffness.

- Subacute combined degeneration of the cord results from long-standing vitamin B12 deficiency, classically presenting as a posterior cord syndrome – with impaired proprioception. It can feature both upper and lower motor neuron signs. B12 deficiency can be associated with several neurological features. These include a myelopathy (classically the subacute combined degeneration of the cord), neuropathy and paraesthesias without neurological signs [3]. Subacute combined degeneration is extremely rare in developed countries, though in tropical countries it is frequently the commonest cause of non-traumatic myelopathy [4].
- Idiopathic Parkinson's disease is a tetrad of Tremor, Rigidity, Akinesia and Postural Instability (this can be remembered using the TRAP mneumonic). In the early stages pain is not a typical feature and it does not cause numbness.
- Multiple Sclerosis [MS] can have a variable presentation, with both sensory and motor symptoms and signs. Inflammatory changes are often present at multiple sites, which can cause symptoms at more than one site; a 'dissociated sensory loss', that is numbness at different and unlinked sites, is a hallmark of MS. Often patients will recall previous episodes of odd neurological deficits, which resolved. MS predominantly affects woman (3-4 times common) and usually presents before the age of 45.

References:

1. Baron EM, Young WF. Cervical spondylotic myelopathy: a brief review of its pathophysiology, clinical course, and diagnosis. Neurosurgery. 2007 Jan;60(1 Supp1 1):S35-41.
2. Behrbalk E, Salame K, Regev GJ, Keynan O, Boszczyk B, Lidar Z. Delayed diagnosis of cervical spondylotic myelopathy by primary care physicians. Neurosurg Focus. 2013 Jul;35(1):E1.
3. Kumar N1. Neurologic aspects of cobalamin (B12) deficiency. Handb Clin Neurol. 2014;120:915-26.

4. Pinto WB, de Souza PV, de Albuquerque MV, Dutra LA, Pedroso JL, Barsottini OG. Clinical and epidemiological profiles of non-traumatic myelopathies. Arq Neuropsiquiatr. 2016 Feb;74(2):161-5.

For peer review only

Select the most likely diagnosis for the following clinical scenarios. Each option can be chosen once or not at all.

A. Multiple sclerosis
B. Aortic aneurysm
C. Fractured clavicle
D. Peripheral neuropathy
E. Degenerative cervical myelopathy
F. Median nerve entrapment
G. Ulnar nerve entrapment
H. Lumbar canal stenosis
I. Saturday night palsy
J. Adhesive Capsulitis

1. A 70 year-old man has pain and weakness in both legs on walking. It settles with rest.
2. A 54 year-old female complains of right hand pain radiating into her thumb, index and middle finger. It often wakes her up from sleep.
3. A 54 year-old female presents with a loss of dexterity in both hands. She has been struggling to type at work and use her mobile phone. Her symptoms have been deteriorating gradually over the preceding months.

Answers

- 1H - lumbar spinal canal stenosis or vascular claudication from peripheral vascular disease are likely. Peripheral vascular disease is more common, but not an option here.
- 2F - Carpal tunnel syndrome results from median nerve compression at the wrist, within the carpal tunnel, and results in lower motor neuron signs, with thenar muscle wasting and weakness of the LOAF muscles (lateral lumbricals, opponens pollicis, abductor pollicis brevis and flexor policis brevis). Patients can have paraesthesias in the median nerve distribution, classically at night. Tinel's test and Phalen's test can be positive.
- 3E - Degenerative cervical myelopathy leads to loss of fine motor function in both upper limbs. There is a delay in diagnosis of degenerative cervical myelopathy, which is estimated to be >2 years in some studies [1]. It is most commonly misdiagnosed as carpal tunnel syndrome and in one study, 43% of patients who underwent surgery for degenerative cervical myelopathy, had been initially diagnosed with carpal tunnel syndrome [1].

References:

For peer review only

1. Behrbalk E, Salame K, Regev GJ, Keynan O, Boszczyk B, Lidar Z. Delayed diagnosis of cervical spondylotic myelopathy by primary care physicians. Neurosurg Focus. 2013 Jul;35(1):E1.

For peer review only

Select the most likely diagnosis for the following clinical scenarios. Each option can be chosen once or not at all.

A. Multiple sclerosis
B. Aortic aneurysm
C. Fractured clavicle
D. Peripheral neuropathy
E. Degenerative cervical myelopathy
F. Median nerve entrapment
G. Ulnar nerve entrapment
H. Lumbar canal stenosis
I. Saturday night palsy
J. Adhesive Capsulitis

1. A 60 year-old male presents with clumsy hands. He has been dropping cups around the house. His wife complains he doesn't answer his mobile as he struggles to use it. His symptoms have been gradually deteriorating over the preceding months.
2. A 32 year-old female presents with a 3 day history of altered sensation on her left foot and right forearm. On examination she has clonus in both legs and has hyperreflexia in all limbs.
3. A 45 year-old female presents with stiffness and pain in her left shoulder, which started around a month ago. She had a similar episode that resolved by itself. Examination reveals limited external rotation.

Answers

- 1E - Degenerative cervical myelopathy leads to loss of fine motor function in both upper limbs. There is a delay in diagnosis of degenerative cervical myelopathy, which is estimated to be >2 years in some studies [1]. It is most commonly misdiagnosed as carpal tunnel syndrome and in one study, 43% of patients who underwent surgery for degenerative cervical myelopathy, had been initially diagnosed with carpal tunnel syndrome [1].
- 2A - Multiple sclerosis (MS) can have a variable presentation, affecting both the sensory and/or motor systems. Inflammatory changes are often present at multiple sites, which can cause symptoms at more than one site; a 'dissociated sensory loss', that is numbness at different and unlinked sites, is a hallmark of MS. Often patients will recall previous episodes of odd neurological deficits, which resolved. MS predominantly affects woman (3-4 times common) and usually presents before the age of 45.

For peer review only

- 3J - Adhesive capsulitis or ‘frozen shoulder’ is most common in the fifth or sixth decade of life. Women are more likely to be affected than men. It is also more common in patients with diabetes mellitus.

References:

1. Behrbalk E, Salame K, Regev GJ, Keynan O, Boszczyk B, Lidar Z. Delayed diagnosis of cervical spondylotic myelopathy by primary care physicians. Neurosurg Focus. 2013 Jul;35(1):E1.

For peer review only

Theme 2: Assessment

A 58 year old gentleman presents with left sided paraesthesias affecting his thumb and first finger. He complains of grip weakness and dropping objects unintentionally. On examination, there is wasting over the thenar eminence. Which of the following signs would suggest a diagnosis other than carpal tunnel syndrome?

A. Positive Hoffman's sign
B. Thenar muscle wasting
C. Unilateral weakness of pincer grip
D. Positive Phalen's test
E. Positive Tinnel's test

Answer: A

A positive Hoffman's sign is a sign of upper motor neuron dysfunction and points to a disease of the central nervous system - in this case from the history degenerative cervical myelopathy [DCM] affecting the cervical spinal cord is most likely. To elicit it, the examiner should flick the patient's distal phalanx (usually of the middle finger) to cause momentary flexion. A positive sign is exaggerated flexion of the thumb.

DCM is often missed initially and there is a delay in the diagnosis of this condition by >2 years in some studies [1]. This is a problem as delayed treatment limits recovery. It is most commonly misdiagnosed as carpal tunnel syndrome and in one study, 43% of patients who underwent surgery for degenerative cervical myelopathy, had been initially diagnosed with carpal tunnel syndrome [1]. DCM is therefore an important differential in patients suspected to have Carpal Tunnel Syndrome [CTS].

CTS is a disease of the peripheral nervous system, resulting from median nerve compression at the wrist inside the carpal tunnel. It therefore affects only the aspects of the hand innervated by the median nerve:

- Sensation; Thumb / Index / Middle Finger. This typically manifests as intermittent pain or parasthesiae.

For peer review only

- Motor; ‘LOAF Muscles’(lateral lumbricals, opponens pollicis, abductor pollicis brevis and flexor policis brevis). Motor signs are less commonly seen with presentations of CTS, but wasting of the thenar eminence may be present.

Tinel’s test and Phalen’s test can be positive, but not always. Both tests aim to increase the pressure within the carpal tunnel, to try to exacerbate symptoms; Tinel’s test via tapping on it and Phalen’s test by sustained full flexion of the wrist.

In focal central nervous system disorders, like DCM, examination features are known to have low sensitivity but high specificity [2]. As a disease of the cervical spinal cord, DCM can affect the sensory, motor and autonomic nervous systems from the neck downwards. Motor signs will be upper motor neuron signs such as increased toned, hyper-reflexia and pyramidal weakness. Note that the neurological signs of DCM are often subtle initially and easily missed, but as a progressive condition they are likely to get worse [3]. Therefore detecting early DCM can be challenging. A high index of suspicion, alongside a comprehensive neurological examination and monitoring for progression is required.

References:

1. Behrbalk E, Salame K, Regev GJ, Keynan O, Boszczyk B, Lidar Z. Delayed diagnosis of cervical spondylotic myelopathy by primary care physicians. Neurosurg Focus. 2013 Jul;35(1):E1.

2. Nicholl DJ, Appleton JP. Clinical neurology: why this still matters in the 21st century. Journal of Neurology, Neurosurgery & Psychiatry 2015;86:229-33.

3. Baron EM, Young WF. Cervical spondylotic myelopathy: a brief review of its pathophysiology, clinical course, and diagnosis. Neurosurgery. 2007 Jan;60(1 Supp1 1):S35-41.

For peer review only

Which of the following statements is most accurate regarding the usefulness of cervical spine radiographs (X-rays) in the assessment of degenerative cervical myelopathy (DCM)?

A. Cervical spine radiographs should be obtained in all patients suspected of having DCM.
B. Where DCM is suspected, AP (anteroposterior), lateral *and* oblique cervical spine radiographs should be requested
C. Cervical spine radiographs are a useful first line investigation where a diagnosis of DCM is suspected
D. Cervical spine radiographs have a low sensitivity but high specificity for DCM
E. Cervical spine radiographs cannot diagnose DCM

Answer: E

Radiographs are of limited value where a diagnosis of degenerative cervical myelopathy is suspected [1] as they cannot visualise the soft tissue, such as the spinal cord.

Spine radiographs have a high sensitivity, but limited specificity to diagnose most spinal conditions. Oblique spine radiographs are usually requested in the lumbar spine region to pick up defects in the pars interarticularis. They have no value in setting of DCM.

The finding of spondylosis is common in spinal x-rays of adults over 40 [2]. Its absence does not exclude neural compression.

Degenerative Cervical Myelopathy [DCM] is spinal cord compression due to degenerative changes of the surrounding spinal structures; e.g. from disc herniation, ligament hypertrophy or calcification, or osteophytes. Therefore in order to visualise these structures, a MRI is gold standard and first line.

Again the presence of such degenerative changes is common on MRI; in one study, 57% of patients older than 64 years of age had disc bulging, though only 26% had spinal cord compression [3]. Therefore a diagnosis of DCM requires the finding of MRI compression in concert with appropriate signs and symptoms.

References

For peer review only

1. Nouri A, Tetreault L, Singh A, Karadimas SK, Fehlings MG. Degenerative Cervical Myelopathy: Epidemiology, Genetics, and Pathogenesis. Spine (Phila Pa 1976). 2015 Jun 15;40(12):E675-93.
2. Baron EM, Young WF. Cervical spondylotic myelopathy: a brief review of its pathophysiology, clinical course, and diagnosis. Neurosurgery. 2007 Jan;60(1 Supp1 1):S35-41.
3. Teresi LM, Lufkin RB, Reicher MA, Moffit BJ, Vinuela FV, Wilson GM, Bentson JR, Hanafee WN: Asymptomatic degenerative disk disease and spondylosis of the cervical spine: MR imaging. Radiology 164:83–88, 1987.

For peer review only

A 67-year-old male undergoes investigations for bilateral paraesthesia in the radial aspects of both hands, over the thumbs and first fingers. He also has paraesthesia in the lateral aspects of both forearms and lower limb spasticity. Blood tests reveal a HBA1c of 46. He undergoes nerve conduction studies and EMG with evidence of denervation. Which ONE of the following diagnoses is most likely?

A. Bilateral carpal tunnel syndrome
B. Degenerative cervical myelopathy
C. Multiple sclerosis
D. Syringomyelia
E. Diabetic neuropathy

Answer: B

This patient's twitches are probably fibrillations, a sign of lower motor neuron dysfunction. This is confirmed on the neurophysiology report, with evidence of denervation. His symptoms are predominantly in the C6 dermatome distribution bilaterally. Although median nerve compression at the elbow bilaterally could in theory produce his symptoms, it would be less likely to explain his symptoms given his age. He is likely to have degenerative cervical myelopathy. This condition is associated with a delay in diagnosis, estimated to be >2 years in some studies [1].

Patients with degenerative cervical myelopathy can present with a number of problems [2]:

- Pain/stiffness: affecting the neck, upper and/or lower limbs. L'hermitte's sign is a sharp pain radiating down the spine on flexion of the neck, which is classically associated with multiple sclerosis, though it can occur in cervical myelopathy.
- Loss of function: Clumsiness (e.g. can't do shirt buttons, hold cup), leg weakness leading to impaired gait, imbalance and falls.
- Sphincter disturbance: this can range from frequency and urgency to incontinence.

Neurological examination can reveal lower motor neuron signs at the level of the lesion and upper motor neuron signs below. Note that neurological signs can be subtle and a high degree of suspicion is needed [2].

For peer review only

The other answers in this question are unlikely for the following reasons:

- A: bilateral carpal tunnel syndrome would not cause forearm symptoms. Carpal tunnel syndrome results from median nerve compression at the wrist and results in a lower motor neuron picture, with thenar muscle wasting and weakness of the LOAF muscles (lateral lumbricals, opponens pollicis, abductor pollicis brevis and flexor policis brevis). Tinel's test and Phalen's test can be positive.
- C: multiple sclerosis (MS) is rare in this age group. MS predominantly affects woman (3-4 times common) and usually presents before the age of 45. It can have a variable presentation, affecting both the sensory and/or motor systems. Inflammatory changes are often present at multiple sites, which can cause symptoms at more than one site; a 'dissociated sensory loss', that is numbness at different and unlinked sites, is a hallmark of MS. Often patients will recall previous episodes of odd neurological deficits, which resolved.
- D: Syringomyelia refers to the development of a syrinx in the spinal cord. It presents with a central cord syndrome, with predominantly upper limb signs. It is a relatively uncommon condition.
- E: His HBA1c is not within the diagnostic range of diabetes mellitus. Diabetes mellitus can cause a peripheral neuropathy presenting in a glove and stocking distribution, as well as neuropathy of peripheral nerves - mononeuritis multiplex.

References:

1. Behrbalk E, Salame K, Regev GJ, Keynan O, Boszczyk B, Lidar Z. Delayed diagnosis of cervical spondylotic myelopathy by primary care physicians. Neurosurg Focus. 2013 Jul;35(1):E1.

2. Baron EM, Young WF. Cervical spondylotic myelopathy: a brief review of its pathophysiology, clinical course, and diagnosis. Neurosurgery. 2007 Jan;60(1 Supp1 1):S35-41.

For peer review only

For peer review only

Which of the following investigations is the most important for diagnosing degenerative cervical myelopathy ?

A. Nerve conduction studies and EMG
B. MRI Cervical spine
C. CT myelogram
D. CT C-spine
E. AP and lateral C-spine radiographs

Answer: B

An MRI of the cervical spine is the gold standard test where cervical myelopathy is suspected. It may reveal disc degeneration and ligament hypertrophy, with accompanying cord signal change.

Other answers:

- CT imaging is reserved for patients with contraindications to magnetic resonance imaging. A CT myelogram is the first line investigation in this case.
- Radiographs are not clinically useful in the workup of these patients, though osteoarthritic changes (e.g. osteophytes) can be visible if they are performed.
- Other investigatons (e.g. nerve conduction studies, EMG) may be performed when the clinical picture is unclear. These can help to exclude mononeuropathies and other lower motor neuron disorders. However, where there is strong clinical suspicion and the diagnosis is suspected, an MRI of the cervical spine should be performed.

For peer review only

A 60-year-old gentleman with a background of lumbar spondylosis and chronic back pain presents with gradually worsening bilateral upper limb paraesthesias and leg stiffness. Which one of the investigations below is diagnostic for his likely condition?

A. Nerve conduction studies and EMG
B. MRI Cervical spine
C. MRI Lumbar Spine
D. CT C-spine
E. AP and lateral C-spine radiographs

Answer: B

The presence of upper limb neurological symptoms indicates that there is pathology either within his cervical spinal cord or brain. Brain disease is more likely to cause unilateral problems.

A MRI lumbar spine would therefore not provide a unifying diagnosis here.

In the context of known lumbar degenerative spine, degenerative cervical myelopathy is the number one differential for this presentation. An MRI of the cervical spine is the gold standard test where cervical myelopathy is suspected. It may reveal disc degeneration and ligament hypertrophy, with accompanying cord signal change. It is not uncommon for patients to suffer from tandem (cervical and lumbar) stenosis.

Other answers:

- CT imaging is reserved for patients with contraindications to magnetic resonance imaging. A CT myelogram is the first line investigation in this case
- Radiographs are not clinically useful in the workup of these patients, though osteoarthritic changes (e.g. osteophytes) can be visible if they are performed.
- Other investigatons (e.g. nerve conduction studies, EMG) may be performed when the clinical picture is unclear. These can help to exclude mononeuropathies and other lower motor neuron disorders. However, where there is strong clinical suspicion and the diagnosis is suspected, an MRI of the cervical spine should be performed.

For peer review only

For peer review only

Select the **most likely** positive examination finding for the following clinical scenarios. Each option can be chosen once, more than once or not at all.

A. Kernig's sign
B. Ankle Brachial Pressure Index
C. Tinel's test
D. Straight leg raise
E. Tongue fasciculations
F. Hoffman's sign
G. Limited external rotation of the shoulder
I. Hypothenar wasting
J. Limited internal rotation of the shoulder

1. A 70 year-old male with a background of diabetes and hypertension presents with pain and weakness in both legs on walking. It settles with rest.
2. A 54 year-old female complains of right hand pain radiating into her thumb, index and middle finger. It often wakes her up from sleep.
3. A 65 year-old female presents with a loss of dexterity in both hands. She has been struggling to type at work and use her mobile phone. Her symptoms have been deteriorating gradually over the preceding months.3

Answers

1 - B: this patient is likely to have peripheral vascular disease [PVD] given his background risk factors for this condition. The ankle brachial pressure index [ABPI] is a simple method of assessing the peripheral circulation. It is calculated by dividing systolic blood pressure in the ankle by the the systolic blood pressure in the arm. These are equal in health (ABPI = 1). The ABPI is reduced in PVD.

2 - C: this patient is likely to have carpal tunnel syndrome. This occurs due to median nerve entrapment beneath the flexor retinaculum. Clinical tests to raise carpal tunnel pressure can exacerbate symptoms and support a diagnosis. One such example is Tinel's test, includes tapping over the volar surface of the wrist joint i.e. over the carpal tunnel, may reproduce paraesthesias. A normal Tinel's test does not exclude carpal tunnel syndrome.

3 - F: this patient is likely to have degenerative cervical myelopathy [DCM], which is associated with upper motor neuron signs. Hoffman's sign is elicited by flicking the distal phalaynx of the middle finger to cause momentary flexion. A positive result is exaggerated flexion of the terminal phalanyx of the thumb. Patients with DCM often have subtle signs that are easily missed [1], but as a progressive condition, these are likely to get worse [2]. Whilst the sensitivity of signs is low (i.e. their absence

For peer review only

does not rule out a problem), their specificity is high (i.e. there will be a problem). Therefore, in order to diagnose early DCM and improve patient outcomes, a high index of suspicion, alongside a 4comprehensive neurological examination and monitoring for progression is required.

Other signs mentioned:

- Kernig's sign refers to painful knee extension, from a position of hip flexion and knee flexion. It suggest meningeal irritation e.g. meningitis, subarachnoid haemorrhage.
- Straight leg raise: this is positively associated with radicular pathology such as disc herniation. The patient feels pain in the back when the leg is raised between 30-60 degrees.
- Limited external rotation is classically found in adhesive capsulitis. Patients have global restriction of shoulder movements, in at least two axes, though external rotation is usually the most affected and painful.

References:

1. Behrbalk E, Salame K, Regev GJ, Keynan O, Boszczyk B, Lidar Z. Delayed diagnosis of cervical spondylotic myelopathy by primary care physicians. Neurosurg Focus. 2013 Jul;35(1):E1.

2. Baron EM, Young WF. Cervical spondylotic myelopathy: a brief review of its pathophysiology, clinical course, and diagnosis. Neurosurgery. 2007 Jan;60(1 Supp1 1):S35-41.

For peer review only

Select the **most likely** positive examination finding for the following clinical scenarios. Each option can be chosen once, more than once or not at all.

A. Kernig's sign
B. Ankle Brachial Pressure Index
C. Tinel's test
D. Straight leg raise
E. Tongue fasciculations
F. Hoffman's sign
G. Limited external rotation of the shoulder
I. Hypothenar wasting
J. Limited internal rotation of the shoulder

1. A 60 year-old male presents with clumsy hands. He has been dropping cups around the house. His wife complains he doesn't answer his mobile as he struggles to use it. His symptoms have been gradually deteriorating over the preceding months.
2. A 32 year-old female presents with a 3 day history of altered sensation of her left foot and right forearm. She had an episode of visual loss a few months ago and says her friends have noted her eyes be flickery and jerky.
3. A 45 year-old female presents with stiffness and pain in her left shoulder, which started around a month ago. She had a similar episode that resolved by itself.

Answers

1 - F: this patient is likely to have degenerative cervical myelopathy [DCM], which is associated with upper motor neuron signs. Hoffman's sign is elicited by flicking the distal phalaynx of the middle finger to cause momentary flexion. A positive result is exaggerated flexion of the terminal phalanyx of the thumb. Patients with DCM often have subtle signs that are easily missed [1], but as a progressive condition, these are likely to get worse [2]. Whilst the sensitivity of signs is low (i.e. their absence does not rule out a problem), their specificity is high (i.e. there will be a problem). Therefore, in order to diagnose early DCM and improve patient outcomes, a high index of suspicion, alongside a comprehensive neurological examination and monitoring for progression is required.

2 - F: this patient is likely to have Multiple Sclerosis (MS). As a disease of the central nervous system, MS is usually associated with only upper motor neuron signs such as Hoffman's sign (see above). The patient's visual loss was probably secondary to optic neuritis, a common presentation of MS. Cerebellar signs are particularly common with MS and include nystagmus, which is likely to be the jerky eye movements noted by her friends.

For peer review only

3 - G: this patient is likely to have adhesive capsulitis. Patients have global restriction of shoulder movements, in at least two axes, though external rotation is classically described as the most affected and painful.

Other signs mentioned:

- Kernig's sign refers to painful knee extension, from a position of hip flexion and knee flexion. It suggest meningeal irritation e.g. meningitis, subarachnoid haemorrhage.
- Straight leg raise: this is positively associated with radicular pathology such as disc herniation. The patient feels pain in the back when the leg is raised between 30-60 degrees.
- The ankle brachial pressure index [ABPI] is a simple method of assessing the peripheral circulation. It is calculated by dividing systolic blood pressure in the ankle by the the systolic blood pressure in the arm. These are equal in health (ABPI = 1). The ABPI is reduced in peripheral vascular disease.
- Tinel's test includes tapping over the volar surface of the wrist joint i.e. over the carpal tunnel. This can reproduce paraesthesias in patients with carpal tunnel syndrome.

References:

1. Behrbalk E, Salame K, Regev GJ, Keynan O, Boszczyk B, Lidar Z. Delayed diagnosis of cervical spondylotic myelopathy by primary care physicians. Neurosurg Focus. 2013 Jul;35(1):E1.

2. Baron EM, Young WF. Cervical spondylotic myelopathy: a brief review of its pathophysiology, clinical course, and diagnosis. Neurosurgery. 2007 Jan;60(1 Supp1 1):S35-41.

For peer review only

Theme 3: Management and follow-up

A 75-year old gentleman presents with a short history of neck pain, paraesthesia in his finger tips and progressive leg weakness. Following a MRI scan of his spine, he is diagnosed with degenerative cervical myelopathy due to a C4/5 disc prolapse. Which of the following is the most appropriate management?

A. Cervical decompressive surgery
B. Cervical nerve root injection
C. Analgesia and referral to physiotherapy
D. Analgesia and review in 4 weeks time
E. Analgesia, a hard cervical collar and review in 4 weeks

Answer: A

All patients with degenerative cervical myelopathy should be urgently referred for assessment by specialist spinal services (neurosurgery or orthopaedic spinal surgery). This is due to the importance of early treatment. The timing of surgery is important, as any existing spinal cord damage can be permanent. Early treatment (within 6 months of diagnosis) offers the best chance of a full recovery but at present, most patients are presenting too late. In one study, patients averaged over 5 appointments before diagnosis, representing >2 years [1].

Currently, decompressive surgery is the only effective treatment. It has been shown to prevent disease progression. Close observation is an option for mild stable disease, but anything progressive or more severe requires surgery to prevent further deterioration. Physiotherapy should only be initiated by specialist services, as manipulation can cause more spinal cord damage.

Prompt diagnosis and onward referral are therefore key to ensuring good outcome for your patients. There are national initiatives to raise awareness of the condition to try and improve referral times (www.myelopathy.org). All of the other listed options in this question do not control the patient's primary pathology.

References:

For peer review only

1. Behrbalk E, Salame K, Regev GJ, et al. Delayed diagnosis of cervical spondylotic myelopathy by primary care physicians. Neurosurg Focus 2013;35:E1. doi:10.3171/2013.3.FOCUS1374

For peer review only

A 65-year old gentleman with a background of osteoarthritis and previous cervical laminectomy for degenerative cervical myelopathy presents with a 2-month history of worsening gait instability and urinary urgency. Which of the following is the most likely explanation for his symptoms?

A. Transverse myelitis
B. Recurrent degenerative cervical myelopathy
C. Multiple sclerosis
D. Cauda equina syndrome
E. Spinal metastases

Answer: B

- Postoperatively, patients with cervical myelopathy require ongoing follow-up as pathology can "recur" at adjacent spinal levels, which were not treated by the initial decompressive surgery. This is called adjacent segment disease. Furthermore, surgery can change spinal dynamics increasing the likelihood of other levels being affected. Patients sometimes develop mal-alignment of the spine, including kyphosis and spondylolisthesis, and this can also affect the spinal cord. All patients with recurrent symptoms should be evaluated urgently by specialist spinal services.
- Transverse myelitis usually presents more acutely than in this case, with a sensory level and upper motor neuron signs below the level affected. It can occur in patients with multiple sclerosis or Devic's disease (neuromyelitis optica). These patients tend to also have features such as optic neuritis.
- Cauda equina syndrome results from compression of the cauda equina and classically includes leg weakness, saddle anaesthesia and sphincter disturbance. This gentleman's history is much more likely to be in keeping with recurrent cervical myelopathy, given his background and given the subacute presentation
- Spinal metastases are uncommon, especially in a patient without a known primary. Given previous DCM, recurrence is more likely.

For peer review only

A 70 year old man has decompressive surgery for degenerative cervical myelopathy. Three years later he presents with neck pain and hand paraesthesias. Which one of the following management strategies is recommended?

A. Trial of neuropathic analgesia and cervical nerve root injections
B. Investigate with nerve conduction studies and EMG in the first instance
C. Urgent AP/lateral cervical spine radiographs as an MRI scan is contraindicated
D. Urgent referral to spinal surgery or neurosurgery
E. Refer to physiotherapy services

Answer: D

Postoperatively, patients with cervical myelopathy require ongoing follow-up as pathology can "recur" at adjacent spinal levels, which were not treated by the initial decompressive surgery.

Recurrent symptoms should be treated with a high degree of suspicion. Although peripheral neuropathy can occur in any patient, this should not be the diagnosis that is the most strongly suspected as delays in diagnosis and treatment of DCM affect outcomes. Therefore, B is false.

All patients with recurrent symptoms should be evaluated urgently by specialist spinal services (A and E, false). Axial spine imaging is necessary and a MRI scan is first line. In patients unable to to have a MRI, CT or CT myelogram may be considered. AP and lateral radiographs are of limited use when myelopathy is suspected (C, false).

References

1. Kong L, Cao J, Wang L, Shen Y. Prevalence of adjacent segment disease following cervical spine surgery: A PRISMA-compliant systematic review and meta-analysis. Medicine (Baltimore). 2016 Jul;95(27):e4171.

For peer review only

A 65-year-old gentleman is referred to neurology outpatients with arm pain, stiffness and imbalance. Following investigations he is diagnosed with degenerative cervical myelopathy. Unfortunately, he misses his next outpatient clinic due to admission with acute coronary syndrome. He attends his GP 2 months later and mentions his ongoing neurological symptoms. Which of the following is the most important next step in his care?

A. Refer to spinal surgery or neurosurgery
B. Refer for cervical nerve root injections
C. Commence neuropathic analgesia
D. Reassure the patient of his diagnosis
E. Refer for physiotherapy

Answer: A

Management of patients with cervical myelopathy should be by specialist spinal services (neurosurgery or orthopaedic spinal surgery). Decompressive surgery is the mainstay of treatment and has been shown to stop disease progression (B, false). Close observation is an option for mild stable disease, but anything progressive or more severe requires surgery to prevent further deterioration. Pre-operative physiotherapy should only be initiated by specialist services, as manipulation can cause more spinal cord damage.

The timing of surgery is important, as any existing spinal cord damage can be permanent. Treatment within 6 months offers the best chance of making a full recovery. At present most patients wait more than 2 years for a diagnosis [1].

Other incorrect options:

- Neuropathic analgesia is important for symptomatic relief but will not prevent further cord damage.
- Physiotherapy does not replace surgical opinion, it can in fact cause more spinal cord damage in patients yet to receive surgical treatment. It should therefore only be initiated by specialist services.

1. Behrbalk E, Salame K, Regev GJ, Keynan O, Boszczyk B, Lidar Z. Delayed diagnosis of cervical spondylotic myelopathy by primary care physicians. Neurosurg Focus. 2013 Jul;35(1):E1.

For peer review only

For peer review only

A 67-year old male recently attended A&E, with a 3 month history of bilateral paraesthesias and twitching affecting the thumb, first finger and lateral forearm. He denied any trauma. A MRI scan of his spine was performed and revealed cervical canal stenosis with mild cord compression. He was discharged and advised to see his GP for follow-up. Which of the following is the most appropriate initial step in management?

A. Refer to spinal surgery services
B. Refer for locally commissioned cervical root injections and review after 6 weeks
C. Enlist on the weekly minor ops clinic for carpal tunnel decompression
D. Commence neuropathic analgesia in the first instance and consider surgical evaluation if this does not work
E. Refer to physiology services and review in 6 weeks

Answer: A

Bilateral median nerve dysfunction is very suggestive of a diagnosis of degenerative cervical myelopathy (DCM) rather than bilateral carpal tunnel syndrome (option C). DCM should be suspected in elderly patients presenting with limb neurology. His twitches are probably fibrillations, a sign of lower motor neuron dysfunction.

Degenerative cervical myelopathy is associated with a delay in diagnosis, estimated to be >2 years in some studies [1]. It is most commonly misdiagnosed as carpal tunnel syndrome and in one study, 43% of patients who underwent surgery for degenerative cervical myelopathy, had been initially diagnosed with carpal tunnel syndrome [1]. Management of these patients should be by specialist spinal services (neurosurgery or orthopaedic spinal surgery). Decompressive surgery is the mainstay of treatment and has been shown to stop disease progression. Physiotherapy and analgesia does not replace surgical opinion, though they may be used alongside (options D and E). Nerve root injections do not have a role in management (option B).

1. Behrbalk E, Salame K, Regev GJ, Keynan O, Boszczyk B, Lidar Z. Delayed diagnosis of cervical spondylotic myelopathy by primary care physicians. Neurosurg Focus. 2013 Jul;35(1):E1.

For peer review only

For peer review only

Select the best management option for the following clinical scenarios. Each option can be chosen once, more than once or not at all.

A) Arrange X-rays of the cervical spine
B) Refer to pain clinic
C) Trial of analgesia and re-review
D) Refer to spinal surgery
E) Refer for physiotherapy
F) Lifestyle modification and co-morbidity optimisation
G) Trial of a cervical collar
H) Refer for a minor ops procedure, division of flexor retinaculum
I) Refer to neurology
J) Refer to elderly care medicine
K) Refer to vascular surgery

1. A 70 year-old male with a background of diabetes and hypertension presents with pain and weakness in both legs on walking. It settles with rest. His most recent HBA1c is 56 and an ankle-brachial pressure index is calculated as 0.7.
2. A 35 year-old female who is 30 weeks pregnant complains of right hand pain radiating into her thumb, index and middle finger. It often wakes her up from sleep.
3. A 65 year-old female presents with neck pain and loss of dexterity in both hands. She has been struggling to type at work and use her mobile phone. Her symptoms have been deteriorating gradually over the preceding months.

Answers

1 - F: this patient has peripheral vascular disease, as evident by his ankle-brachial pressure index (ABPI). NICE guidance suggests that first line management should include lifestyle modification such as smoking cessation, weight loss, lipid modification, optimisation of diabetes mellitus/hypertension and antiplatelet therapy. A supervised exercise programme can also be arranged. Local guidelines vary on when referral to specialist care is needed, but typically this would be where conservative treatment fails after 3 months or the ABPI is below a defined threshold (e.g. <0.6).

2 - E: this patient is likely to have carpal tunnel syndrome. This occurs due to median nerve entrapment beneath the flexor retinaculum. It is more common in pregnant women due to the increase in oedema. NICE clinical knowledge summaries (CKS) recommend lifestyle measures (e.g. wrist ergonomic devices at work), as well as wrist splints (usually prescribed by physiotherapists), corticosteroid injections or referral for surgical management. Wrist splints can be

For peer review only

helpful for nighttime symptoms, as in her case. Corticosteroid injections require local expertise that may or may not be present.

3 - D: this patient is likely to have degenerative cervical myelopathy. DCM is often missed initially and there is a delay in the diagnosis of this condition by >2 years in some studies [1]. Patients have predominantly upper motor neuron signs such as increased toned, hyper-reflexia and pyramidal weakness. Neurological signs are often subtle initially and easily missed, but as a progressive condition they are likely to get worse [2]. Management of these patients should be by specialist spinal services (neurosurgery or orthopaedic spinal surgery). An MRI scan is required for diagnosis. All patients should be assessed by a spinal surgeon.

References:

1. Behrbalk E, Salame K, Regev GJ, Keynan O, Boszczyk B, Lidar Z. Delayed diagnosis of cervical spondylotic myelopathy by primary care physicians. Neurosurg Focus. 2013 Jul;35(1):E1.

2. Baron EM, Young WF. Cervical spondylotic myelopathy: a brief review of its pathophysiology, clinical course, and diagnosis. Neurosurgery. 2007 Jan;60(1 Supp1 1):S35-41.

For peer review only

Select the best management option for the following clinical scenarios. Each option can be chosen once, more than once or not at all.

A) Arrange X-rays of the cervical spine
B) Refer to pain clinic
C) Trial of analgesia and re-review
D) Refer to spinal surgery
E) Refer for physiotherapy
F) Lifestyle modification and co-morbidity optimisation
G) Trial of a cervical collar
H) Refer for a minor ops procedure, division of flexor retinaculum
I) Refer to neurology
J) Refer to elderly care medicine
K) Refer to vascular surgery

1. A 60 year-old male presents with clumsy hands. He has been dropping cups around the house. His wife complains he doesn't answer his mobile as he struggles to use it. His symptoms have been gradually deteriorating over the preceding months.
2. A 32 year-old female presents with a 3 day history of altered sensation on her left foot and right forearm. She had an episode of visual blurring in her right eye a few months ago which resolved after a few days. Examination reveals brisk reflexes.
3. A 45 year-old female presents with stiffness and pain in her left shoulder, which started around a month ago. She had a similar episode that resolved by itself. Examination reveals global restriction of shoulder movement, particularly external rotation.

Answers

1 - D: this patient is likely to have degenerative cervical myelopathy. DCM is often missed initially and there is a delay in the diagnosis of this condition by >2 years in some studies [1]. Patients have predominantly upper motor neuron signs such as increased toned, hyper-reflexia and pyramidal weakness. Neurological signs are often subtle initially and easily missed, but as a progressive condition they are likely to get worse [2]. Management of these patients should be by specialist spinal services (neurosurgery or orthopaedic spinal surgery). An MRI scan is required for diagnosis. All patients should be assessed by a spinal surgeon.

2 - I: this patient is likely to have Multiple Sclerosis. Her visual loss was probably secondary to optic neuritis, a common ophthalmological association with multiple sclerosis. She should be referred to neurology.

3 - C: this patient is likely to have adhesive capsulitis (frozen shoulder). Patients have global restriction of shoulder movements, in at least two axes, though external rotation is classically described as the most affected and painful. Management of frozen shoulder is controversial and there is not much evidence to inform practice.

For peer review only

In general, alternative diagnoses should be excluded and pain relief optimised. Gentle shoulder movement is encouraged and there is limited evidence for physiotherapy.

References:

1. Behrbalk E, Salame K, Regev GJ, Keynan O, Boszczyk B, Lidar Z. Delayed diagnosis of cervical spondylotic myelopathy by primary care physicians. Neurosurg Focus. 2013 Jul;35(1):E1.

2. Baron EM, Young WF. Cervical spondylotic myelopathy: a brief review of its pathophysiology, clinical course, and diagnosis. Neurosurgery. 2007 Jan;60(1 Supp1 1):S35-41.

For peer review only

BMJ Open

A quantitative analysis of medical students' and physicians' knowledge of degenerative cervical myelopathy

Journal:	*BMJ Open*
Manuscript ID	bmjopen-2018-028455.R1
Article Type:	Research
Date Submitted by the Author:	26-Apr-2019
Complete List of Authors:	Waqar, Mueez; University of Manchester, Academic Neurosurgery<br>Wilcock, Jane; University of Liverpool, Department of Undergraduate Medical Education<br>Garner, Jayne; University of Liverpool, Department of Undergraduate Medical Education<br>Davies, Benjamin; University of Cambridge, Department of Clinical Neurosurgery<br>Kotter, Mark; University of Cambridge, Department of Clinical Neurosurgery; Ann McLaren Laboratory of Regenerative Medicine, Department of Clinical Neurosciences
**Primary Subject Heading**:	Neurology
Secondary Subject Heading:	Neurology, Medical education and training, General practice / Family practice
Keywords:	Cervical myelopathy, spondylosis, degenerative spine, physician knowledge, gap analysis

SCHOLARONE™
Manuscripts

A quantitative analysis of medical students' and physicians' knowledge of degenerative cervical myelopathy

Authors and affiliations

1. Mueez Waqar[1], mueez.waqar@manchester.ac.uk
2. Jane Wilcock[2], Jane.Wilcock@liverpool.ac.uk
3. Jayne Garner[2], jayneg@liverpool.ac.uk
4. Benjamin M Davies[3,4], bd375@cam.ac.uk
5. Mark RN Kotter[3,4], mrk25@cam.ac.uk

[1]Department of Academic Neurosurgery, Manchester Academic Health Sciences Centre, The University of Manchester, Manchester, UK

[2]Department of Undergraduate Medical Education, The University of Liverpool, Liverpool, UK

[3]Department of Academic Neurosurgery, Cambridge University Hospital, University of Cambridge, Cambridge, UK

[4]WT MRC Cambridge Stem Cell Institute, Anne McLaren Laboratory, University of Cambridge, UK

Corresponding author

Dr. Mark R.N. Kotter

WT MRC Cambridge Stem Cell Institute and Department of Clinical Neurosciences

University of Cambridge

For peer review only - http://bmjopen.bmj.com/site/about/guidelines.xhtml

Acknowledgements

The authors would like to thank Passmedicine for their assistance with this work.

Declarations and conflicts of interest

Research in the senior author's laboratory is supported by a core support grant from the Wellcome Trust and MRC to the Wellcome Trust-Medical Research Council Cambridge Stem Cell Institute. MRNK is supported by a NIHR Clinician Scientist Award.

Disclaimer: This report is independent research arising from a Clinician Scientist Award, CS-2015-15-023, supported by the National Institute for Health Research. The views expressed in this publication are those of the authors and not necessarily those of the NHS, the National Institute for Health Research or the Department of Health and Social Care.

Word count

2425

Data

Additional data is provided in supplementary table 1.

Author statement

Each author met ICMJE criteria for authorship:

- MW: coordinated creation of questions, performed statistical analysis, wrote up all manuscript drafts.
- JW: created first draft of questions for question-bank, aided in manuscript analysis and write-up, helped to check of final draft.

For peer review only - http://bmjopen.bmj.com/site/about/guidelines.xhtml

- JG: aided in creation of first draft of questions for question-bank, aided in manuscript analysis and checking of final draft.
- BMD: checked questions, oversaw data analysis, aided in authoring introduction and collecting key references, helped to devise project idea, checked final manuscript draft.
- MRNK: devised study idea, provided draft protocol, checked questions, edited final manuscript draft.

Funding

Research in the senior author’s laboratory is supported by a core support grant from the Wellcome Trust and MRC to the Wellcome Trust-Medical Research Council Cambridge Stem Cell Institute. MRNK is supported by a NIHR Clinician Scientist Award.

Disclaimer: This report is independent research arising from a Clinician Scientist Award, CS-2015-15-023, supported by the National Institute for Health Research. The views expressed in this publication are those of the authors and not necessarily those of the NHS, the National Institute for Health Research or the Department of Health and Social Care.

Data availability statement

No additional data available in addition to that presented in the supplement.

For peer review only - http://bmjopen.bmj.com/site/about/guidelines.xhtml

Abstract

Objectives. We have previously identified a delay in general practitioner (GP) referrals for patients with degenerative cervical myelopathy (DCM). The aim of this study was to evaluate whether an education gap existed for DCM along the GP training pathway by quantitatively assessing training in, and knowledge of, this condition.

Design. Gap analysis: comparison of DCM to other conditions. Comparators selected on the basis of similar presentation/epidemiology (multiple sclerosis), an important spinal emergency (cauda equina syndrome) and a common disease (diabetes mellitus).

Subjects. Medical students, foundation doctors and GP trainees.

Primary and secondary outcome measures. (1) Assessment of training: quantitative comparison of references to DCM in curricula (undergraduate/postgraduate) and commonly used textbooks (Oxford Handbook Series), to other conditions using modal ranks. (2) Assessment of knowledge: using standardised questions placed in an online question-bank (PassMedicine). Results were presented relative to the question-bank mean (+/-).

Results. DCM had the lowest modal rank of references to the condition in curricula analysis and 2nd lowest modal rank in textbook analysis. In knowledge analysis questions were attempted 127,457 times. Performance for DCM questions in themes of presentation (+6.1%) , workup (+0.1%) and management (+1.8%) were all greater than the question-bank mean and within one standard deviation. For students and junior trainees, there was a serial decrease in performance from

For peer review only - http://bmjopen.bmj.com/site/about/guidelines.xhtml

presentation and workup (-0.7% to +10.4% relative to question-bank mean) and management (-0.6% to -3.9% relative to question-bank mean).

Conclusions. Although infrequently cited in curricula and learning resources, knowledge relating to DCM was above average. However, knowledge relating to its management was relatively poor.

Keywords

Cervical myelopathy; spondylosis; degenerative spine; physician knowledge; gap analysis

Strengths and limitations of this study

- Strength: Search terms relating to DCM were queried from three UK specific medical school curricula and relevant postgraduate curricula
- Strength: a large number of responses were obtained by placing questions in an online question-bank, relating to DCM
- Limitation: A limited number of learning resources were searched to assess references to DCM

For peer review only - http://bmjopen.bmj.com/site/about/guidelines.xhtml

Introduction

Degenerative cervical myelopathy (DCM) is a common and insidious condition that can lead to severe disability.[1] It arises when degenerative changes of the spine compress the spinal cord, causing a progressive spinal cord injury. Currently, treatment is limited to surgical decompression, which is able to stop further injury but due to the limited capacity of the spinal cord to repair, recovery is limited. The timing of surgery is therefore crucial to recovery ('time is spine'), and a recent meta-analysis has demonstrated treatment within 6 months offers a greater chance of making a full recovery.[2] Unfortunately, few patients are diagnosed promptly, with the majority waiting more than 2 years for a diagnosis.[3] Consequently, most patients retain life-long disabilities, contributing to quality of life scores lower than cancer, heart and lung diseases.[4, 5]

The diagnostic pathway for DCM almost exclusively starts with assessment and triage by a community physician, termed in the UK a General Practitioner (GP).[3] If appropriate, the patient is then referred on for further investigation and management. Our analysis of this pathway has identified time to initial referral by GP (6.4±7.7 months) as representing 51% of diagnostic delay.[6]

This period of the diagnostic pathway is difficult to examine in detail, and whilst delayed patient presentation is likely to contribute, delayed detection measured by multiple consultations and patient perspective, is certainly a relevant component.[3,]

For peer review only - http://bmjopen.bmj.com/site/about/guidelines.xhtml

[7] The cause for delayed detection is also likely to be multi-faceted, including subtle, non-specific symptoms and incomplete clinical examination.[1] However, it is conceivable that a lack of understanding of the workup and management of DCM may also contribute. Indeed, DCM would be included within the spectrum of "neurophobia" – an aversion to the neurosciences due to perceived difficulty, which has been demonstrated in GPs and GP trainees.[8, 9]

Our objective therefore was to evaluate whether an education gap exists for DCM amongst GPs, by quantitatively assessing their training in, and knowledge of, DCM.

Methods

Ethics

The present study design did not require ethical approval.

Patient and Public Involvement

DCM patients were surveyed online and this confirmed that the diagnosis of DCM is frequently delayed.[10] The question of whether this is due to lack of knowledge amongst health professionals and whether this was the consequence of a gap in medical education was formulated with the input of DCM sufferers at the First Cambridge Myelopathy day.

For peer review only - http://bmjopen.bmj.com/site/about/guidelines.xhtml

Education Gap Analysis

A gap analysis is a process to identify gaps in existing systems such as education curricula. It involves an assessment of existing knowledge or standards against pre-determined standards that define core competency requirements.[11, 12] This differs, for example, from simple cross sectional knowledge assessments whose primary outcome is usually assessment of knowledge beyond core requirements.

The study objective was therefore approached in two separate gap analyses (Table 1):

1. **Assessment of Training**: quantification of DCM in curricula and commonly used learning resources, including assessment of relative importance through comparison to other conditions.
2. **Assessment of Knowledge**: formal assessment of trainees' knowledge using questions placed in an online question-bank.

The GP Training Pathway

UK medical graduates complete the UK Foundation Programme to enter higher specialty training as formal GP trainees. There are several assessments along this route including: medical school final examinations to gain registration to practice in the UK, or the equivalent Professional and Linguistic Assessments Board test (PLAB) for international medical graduates; the Specialty Recruitment Assessment

For peer review only - http://bmjopen.bmj.com/site/about/guidelines.xhtml

(SRA) – a written assessment taken towards the start of the second foundation year prior to higher specialty applications; and the Membership of The Royal College of General Practitioner's (MRCGP), an exit exam for GP trainees. Whilst alternative entry routes to General Practice exist, this pathway represents the most common training route for GPs today.

Definition of DCM and comparators

DCM was chosen as an inclusive term for a variety of diseases resulting in compressive myelopathy.[1] Comparator diseases were also selected to compare findings to:

1. **Direct comparator to myelopathy:** a disease that is a differential diagnosis for DCM with equivalent or greater incidence.
2. **Degenerative spine comparator**: an alternative degenerative spine disease that is widely taught.
3. **Generic non-neuroscience comparator**: a common disease that all clinicians would have some knowledge about and interaction with.

The *a priori* hypothesis was, if an education gap existed within DCM, we would expect metrics of training and knowledge of DCM to rank inferior to controls. Controls were identified through a consensus author meeting and finalised after confirmation of epidemiological profiles through literature review.

For peer review only - http://bmjopen.bmj.com/site/about/guidelines.xhtml

Assessment of Training: Learning Resource Analysis

We selected learning resources that were in common usage by trainees, available in electronic format (to be amenable for searching) and stratifiable by training stage. Specifically, training curricula, the Oxford Handbook series [13] and online question-banks were selected. Alongside the United Kingdom Foundation Programme and MRCGP, an example from each of the three main, UK medical school teaching models [14] were included: problem-based learning (The University of Manchester, UK); traditional lecture based (The University of Cambridge, UK); and an integrated system including aspects of both (Imperial College London, UK). The choice of medical school curricula was pragmatically selected, based on access. The handbooks most pertinent to the GP training pathway were included in our analysis: The Oxford Handbook of Clinical Medicine (OHCM), The Oxford Handbook for the Foundation Programme (OHFP) and The Oxford Handbook of General Practice (OHGP).

Search terms for analysing learning resources were created for DCM and each comparator using relevant terms from the search syntax of Cochrane Reviews, or if absent, recent systematic reviews. Curricula were searched for the number of references per disease, text books for the number of words per disease section and question banks for the number of questions.

For peer review only - http://bmjopen.bmj.com/site/about/guidelines.xhtml

Assessment of Knowledge

The author panel includes GPs with an interest in education (JW), university appointed educationalists (JG) and neurosurgeons (MW, BMD, MRK). The authors devised a set of questions to be included in an online question bank, composed of both multiple choice questions [MCQs] and extended matching questions [EMQs], designed to cover the different components of medical assessment; presentation, investigation and management. The final set consisted of 19 questions (13 multiple choice questions [MCQs] and 6 extended matching questions [EMQs]). The questions together with answers are included in Supplement 1.

Online question-banks including subsections devoted to each stage of the GP training pathway were contacted with details of the study. Only one question-bank - Passmedicine [15], responded and was therefore selected for this arm of the study.

Analysis

Statistical analysis was performed using SPSS version 22 (SPSS Inc., Chicago, Illinois, USA). For assessment of training, frequencies of search terms were described and the mode and modal ranks determined. For assessment of knowledge, histograms were constructed with either a condition or question-bank along the X-axis and user performance relative to question-bank mean along the Y-axis. The user performance relative to question-bank mean corresponded to the raw difference

For peer review only - http://bmjopen.bmj.com/site/about/guidelines.xhtml

between the mean value for each theme and the mean value for a particular question-bank.

Results

Selection of Comparators and Resources

Multiple sclerosis (MS), cauda equina syndrome (CES) and diabetes mellitus were selected as the three disease comparators and list of search terms compiled accordingly (Table 2).[16-19] MS is a common differential for DCM,[20] with overlapping signs and symptoms. Whilst DCM is likely to be more prevalent in reality,[1] currently their characterised epidemiology is comparable.[21, 22] CES is uncommon, but its missed or delayed diagnosis carries significant consequences. Diabetes is relevant to all medical fields. The question-bank also provided data on neurology as a theme, encompassing all questions relating to the central and peripheral nervous system.

Assessment of training

Curricula analysis

DCM and cauda equina syndrome had the lowest modal rank in curricula search analysis (Table 3). This was true for early stage undergraduate curricula and late

For peer review only - http://bmjopen.bmj.com/site/about/guidelines.xhtml

stage curricula for the foundation programme and MRCGP. Multiple sclerosis was mentioned less frequently in early versus late stage curricula. The opposite trend was observed for diabetes mellitus.

Textbook analysis

Overall, DCM had the second lowest modal rank but above that of cauda equina syndrome (Table 4). The relative word count attributed to neurological conditions such as DCM, multiple sclerosis and cauda equina syndrome decreased with advancing stage of textbook. The opposite trend was observed for diabetes mellitus, for whom the total word count increased.

Question Bank Analysis

Assessment of Knowledge

Passmedicine was approached, on the basis it did not have any DCM questions previously which could influence analysis. Questions were introduced between the period June 2017 – October 2017. Section editors for each question-bank ultimately selected which of the 19 questions were relevant, such that finals/PLAB contained 14, SRA – 19 and MRCGP – 19.

Overall, questions were attempted 127,457 times; finals/PLAB - 36706; SRA - 47530; MRCGP – 43221 (Supplementary Table 1). Data on the number of unique attempts or first-time viewers was not extractable.

For peer review only - http://bmjopen.bmj.com/site/about/guidelines.xhtml

There were differences in user performance in the three DCM question themes – presentation, workup and management, though average scores were all within 1 standard deviation of the mean (Fig. 1). Performance sequentially decreased across these themes for the finals/PLAB group (presentation +4.7%, workup +4.2%, management -0.6%; relative to question-bank mean), SRA group (presentation +10.4%, workup -0.7%, management -3.9%; relative to question-bank mean) and when all groups were considered together (presentation +6.1%, workup +0.1%, management +1.8%; relative to question-bank mean). In the MRCGP group however, performance clustered around the mean for the question bank with little variability (presentation -0.1%, workup +1.0%, management +1.8%; relative to question-bank mean).

User performance by disease showed large variation between question-banks (Fig. 2). The mean user performance decreased sequentially for DCM with advancing question-bank (finals/PLAB +4.2%, SRA +2.7%, MRCGP +0.9%; relative to question-bank mean). However, the mean user performance for DCM was always greater than the question-bank mean (+2.6% relative to question-bank mean).

Discussion

In this study, we found that DCM is relatively infrequently cited in curricula and commonly used textbooks, even compared to diseases such as multiple sclerosis, with similar epidemiology. Despite this however, user performance in some of the

For peer review only - http://bmjopen.bmj.com/site/about/guidelines.xhtml

DCM questions remained consistently above question-bank averages. There was a sequential decrease in user performance across the themes of DCM presentation, workup and management for early years' trainees, whereas for senior trainees, performance did not vary by theme of question. For other neuroscience themes such as multiple sclerosis and cauda equina syndrome, the user performance was below average. This was in contrast to user performance in questions relating to diabetes mellitus, which was consistently above question-bank mean.

We observed a below average performance in questions grouped under the neurology theme, which included all questions relating to the central and peripheral nervous system. Reduced knowledge pertaining to neurosciences has previously been linked to a term called neurophobia, though this is by no means a universally accepted concept.[8, 9, 23] In one questionnaire study for example, GPs rated neurology as the most difficulty medical specialty and the one for which they had the least confidence compared to cardiology, endocrinology, gastroenterology, geriatrics, respiratory medicine and rheumatology.[9] However, the question-bank data did not allow distinction between basic science and clinical questions, for whom the performance may be different, as evidenced by the above average performance in our clinically orientated DCM questions.

Secondly, our study supports the observation of poorer performance in management themes by clinicians at an earlier training stage. Prior studies on

For peer review only - http://bmjopen.bmj.com/site/about/guidelines.xhtml

medical students that have evaluated knowledge formally have shown similar results in specialties such as ophthalmology.[24] This also appears to translate into patient encounters during clinical assessments in neurology.[25, 26] In one study evaluating student performance in neurology outpatients, students were more likely to make errors regarding diagnostic tests or planning treatment than lesion localisation.[25] Another study evaluating student performance in neurology themed objective structured clinical examinations (OSCEs) also reported similar results, finding poorer student performance in supportive management of neurological conditions versus diagnosis.[26]

Our original hypothesis attempting to correlate delays in diagnosis for DCM with a deficiency in training and knowledge, was not conclusively demonstrated in the present study. Earlier stage trainee doctors demonstrated comparably higher levels of knowledge with regards to the presentation and work up of DCM and a potential gap with regards to management-related questions. These differences disappeared in individuals studying for the GP exit exams. Nevertheless, the clinical argument for an education gap is strong - our previous analysis of the referral pathway for DCM identified a time to initial referral by the GP as representing 51% of the overall diagnostic delay.

This study demonstrated DCM received relatively less training than other conditions. In the West, the prevalence of DCM is underestimated but at least 60

For peer review only - http://bmjopen.bmj.com/site/about/guidelines.xhtml

per 100,000, compared to around 100 per 100,000 for multiple sclerosis.[21, 22] Despite this however, DCM was under-represented by eight-fold compared to multiple sclerosis in curricula analysis. In late stage curricula (foundation programme, MRCGP), DCM was not referred to at all. A similar trend was observed in textbook analysis, though neither of these analyses were exhaustive. These results were in contrast to our knowledge analysis, which showed consistently above average performance for DCM unlike multiple sclerosis.

One potential way of reconciling the absence of a gap in formal knowledge with the deficiencies in the diagnosis and management of DCM, is that factual knowledge may not translate into recognition of the disease in the clinical context. Causal association between training and clinical practice have not been clearly demonstrated, albeit prior studies have found good correlation between exam performance and clinical performance in a variety of medical and surgical specialties.[27-29] Most of this data is based on the United States Medical Licensing Examination (USMLE), which appears to correlate well with residency performance.[27, 28] Beyond medical school exams, in another study, internal medicine clinicians failing their maintenance of certification exams had a more than double chance of disciplinary action versus those who passed.[29] An alternative explanation is that questionnaire based methods may not be sufficiently sensitive to detect poor clinical decision making in the context of DCM. Future studies should consider

For peer review only - http://bmjopen.bmj.com/site/about/guidelines.xhtml

employing mock patients for the condition in undergraduate and postgraduate OSCEs, to compare performance to other conditions.

There are several limitations to be considered in the context of these findings. Firstly, only select learning resources were used in this study and questions were inserted into a single online question bank. Secondly, question bank data was not extractable per user, nor per first time answer. Whilst this was not a comprehensive strategy, this is unlikely to have limited our results for at least two reasons: questions were attempted more than 100,000 times and analysis was relative within learning resources, and therefore limitations were applicable to each comparator. The native question bank data extraction technique also did not provide data on the number of attempts on question themes other than DCM.

The methodology employed in the present study may not have been sufficiently sensitive to detect a knowledge gap for DCM, for several reasons. Firstly, our questions may not have been challenging enough given that they tested core principles all clinicians would be expected to achieve. Although we did not employ pilot testing of the questions in our target population, questions were designed by an experienced author panel including educationalists. Furthermore, our questions were subject to additional scrutiny by the question-bank editors, such that only those questions deemed appropriate were included in a particular question-bank. Secondly, there was no first-time answer data available for analysis. This means

For peer review only - http://bmjopen.bmj.com/site/about/guidelines.xhtml

that 'random effect answering', users selecting an answer to read the explanation, was not accounted for, though this was the case for both DCM and controls. Thirdly, our controls may not have been appropriate and certainly, educational comparisons between specialties is not always the best method of assessing knowledge adequacy.[30] Finally, our methodology assumed a direct correlation between "on paper" knowledge and clinical care provided to patients – though this was not proven in the present study and could still differ vastly.

Conclusion

In this study, we set out to evaluate whether there is a deficiency in training and knowledge regarding DCM in the GP training pathway. Although DCM was infrequently referenced in learning resources, trainees performed above average for DCM questions in an assessment of knowledge using an online question-bank. The clinical suggestion of an education gap is strong, and these contrasting findings do not conclusively allay this concern. Future studies are required to better understand these observations.

References

1. Davies BM, Mowforth OD, Smith EK, et al. Degenerative cervical myelopathy. *BMJ* 2018;360:k186. doi: 10.1136/bmj.k186 [published Online First: 2018/02/24]
2. Fehlings MG, Tetreault LA, Riew KD, et al. A Clinical Practice Guideline for the Management of Patients With Degenerative Cervical Myelopathy: Recommendations for Patients With Mild, Moderate, and Severe Disease and

For peer review only - http://bmjopen.bmj.com/site/about/guidelines.xhtml

Nonmyelopathic Patients With Evidence of Cord Compression. *Global spine journal* 2017;7(3 Suppl):70S-83S. doi: 10.1177/2192568217701914 [published Online First: 2017/11/23]

3. Behrbalk E, Salame K, Regev GJ, et al. Delayed diagnosis of cervical spondylotic myelopathy by primary care physicians. *Neurosurgical focus* 2013;35(1):E1. doi: 10.3171/2013.3.FOCUS1374 [published Online First: 2013/07/03]
4. Fehlings MG, Ibrahim A, Tetreault L, et al. A global perspective on the outcomes of surgical decompression in patients with cervical spondylotic myelopathy: results from the prospective multicenter AOSpine international study on 479 patients. *Spine* 2015;40(17):1322-8. doi: 10.1097/BRS.0000000000000988 [published Online First: 2015/05/29]
5. Oh T, Lafage R, Lafage V, et al. Comparing Quality of Life in Cervical Spondylotic Myelopathy with Other Chronic Debilitating Diseases Using the Short Form Survey 36-Health Survey. *World Neurosurg* 2017;106:699-706. doi: 10.1016/j.wneu.2016.12.124 [published Online First: 2017/01/10]
6. Hilton B T-MJ, Davies BM, Kotter MRN. Route to diagnosis of degenerative cervical myelopathy in a UK healthcare system: a retrospective cohort study. *BMJ Open (in-press)* 2018
7. Elemental Ideas – Cervical Spondylotic Myelopathy Part One [cited 2018 October 12th]. Available from: http://archive.cambridge-tv.co.uk/elemental-ideas-cervical-spondylotic-myelopathy-part-one/ accessed October 12th 2018.
8. McCarron MO, Stevenson M, Loftus AM, et al. Neurophobia among general practice trainees: the evidence, perceived causes and solutions. *Clinical neurology and neurosurgery* 2014;122:124-8. doi: 10.1016/j.clineuro.2014.03.021 [published Online First: 2014/05/06]
9. Loftus AM, Wade C, McCarron MO. Primary care perceptions of neurology and neurology services. *Postgraduate medical journal* 2016;92(1088):318-21. doi: 10.1136/postgradmedj-2015-133683 [published Online First: 2016/01/23]
10. Davies BM, Munro CF, Kotter MR. A Novel Insight Into the Challenges of Diagnosing Degenerative Cervical Myelopathy Using Web-Based Symptom Checkers. *J Med Internet Res* 2019;21(1):e10868. doi: 10.2196/10868 [published Online First: 2018/10/10]

For peer review only - http://bmjopen.bmj.com/site/about/guidelines.xhtml

11. Fater KH. Gap analysis: a method to assess core competency development in the curriculum. *Nurs Educ Perspect* 2013;34(2):101-5. [published Online First: 2013/06/15]

12. O'Brien CE, Stafford R, Franks AM. Establishment of a patient-centered communication course to address curricular gaps. *Curr Pharm Teach Learn* 2018;10(7):933-39. doi: 10.1016/j.cptl.2018.04.008 [published Online First: 2018/09/22]

13. Warriner D. The Oxford Handbook of Clinical Medicine. *BMJ* 2008;336(7640):393-93. doi: 10.1136/bmj.39451.675451.59

14. NHS careers. Teaching styles at medical school [27/05/2018]. Available from: https://www.healthcareers.nhs.uk/sites/default/files/documents/Teaching styles at medical school.pdf accessed 27/05/2018.

15. Passmedicine 2018 [cited 2018 12th October]. Available from: https://www.passmedicine.com/ accessed 12 October 2018.

16. Kiely PD, Quinn JC, Du JY, et al. Posterior surgical treatment of cervical spondylotic myelopathy: review article. *HSS J* 2015;11(1):36-42. doi: 10.1007/s11420-014-9425-5 [published Online First: 2015/03/05]

17. Khan F, Amatya B. Rehabilitation in Multiple Sclerosis: A Systematic Review of Systematic Reviews. *Arch Phys Med Rehabil* 2017;98(2):353-67. doi: 10.1016/j.apmr.2016.04.016 [published Online First: 2016/05/25]

18. Chau AM, Xu LL, Pelzer NR, et al. Timing of surgical intervention in cauda equina syndrome: a systematic critical review. *World Neurosurg* 2014;81(3-4):640-50. doi: 10.1016/j.wneu.2013.11.007 [published Online First: 2013/11/19]

19. Richter B, Bandeira-Echtler E, Bergerhoff K, et al. Pioglitazone for type 2 diabetes mellitus. *Cochrane Database Syst Rev* 2006(4):CD006060. doi: 10.1002/14651858.CD006060.pub2 [published Online First: 2006/10/21]

20. Davies BM, Munro CF, Kotter M. A Novel Insight into the Challenges of Diagnosing Degenerative Cervical Myelopathy using Online Symptom Checkers. *J Med Internet Res* 2018 doi: 10.2196/10868 [published Online First: 2018/10/10]

21. Nouri A, Tetreault L, Singh A, et al. Degenerative Cervical Myelopathy: Epidemiology, Genetics, and Pathogenesis. *Spine* 2015;40(12):E675-93. doi: 10.1097/BRS.0000000000000913 [published Online First: 2015/04/04]

For peer review only - http://bmjopen.bmj.com/site/about/guidelines.xhtml

22. Dilokthornsakul P, Valuck RJ, Nair KV, et al. Multiple sclerosis prevalence in the United States commercially insured population. *Neurology* 2016;86(11):1014-21. doi: 10.1212/WNL.0000000000002469 [published Online First: 2016/02/19]

23. Flanagan E, Walsh C, Tubridy N. 'Neurophobia'--attitudes of medical students and doctors in Ireland to neurological teaching. *Eur J Neurol* 2007;14(10):1109-12. doi: 10.1111/j.1468-1331.2007.01911.x [published Online First: 2007/09/21]

24. Esparaz ES, Binder SB, Borges NJ. How prepared are medical students to diagnose and manage common ocular conditions. *J Educ Eval Health Prof* 2014;11:29. doi: 10.3352/jeehp.2014.11.29 [published Online First: 2014/11/25]

25. Davis LE, King MK. Assessment of medical student clinical competencies in the neurology clinic. *Neurology* 2007;68(8):597-9. doi: 10.1212/01.wnl.0000254490.24555.f7 [published Online First: 2007/02/21]

26. Lukas RV, Blood A, Park YS, et al. Assessment of neurological clinical management reasoning in medical students. *Journal of clinical neuroscience : official journal of the Neurosurgical Society of Australasia* 2014;21(6):919-22. doi: 10.1016/j.jocn.2013.09.006 [published Online First: 2013/12/07]

27. Thundiyil JG, Modica RF, Silvestri S, et al. Do United States Medical Licensing Examination (USMLE) scores predict in-training test performance for emergency medicine residents? *J Emerg Med* 2010;38(1):65-9. doi: 10.1016/j.jemermed.2008.04.010 [published Online First: 2008/10/28]

28. Sutton E, Richardson JD, Ziegler C, et al. Is USMLE Step 1 score a valid predictor of success in surgical residency? *Am J Surg* 2014;208(6):1029-34; discussion 34. doi: 10.1016/j.amjsurg.2014.06.032 [published Online First: 2014/12/03]

29. McDonald FS, Duhigg LM, Arnold GK, et al. The American Board of Internal Medicine Maintenance of Certification Examination and State Medical Board Disciplinary Actions: a Population Cohort Study. *J Gen Intern Med* 2018 doi: 10.1007/s11606-018-4376-z [published Online First: 2018/03/09]

30. Di Paola M, Bennet GC, Shearer JR. Orthopaedic teaching in United Kingdom medical schools. *Med Teach* 1986;8(2):155-8. [published Online First: 1986/01/01]

For peer review only - http://bmjopen.bmj.com/site/about/guidelines.xhtml

For peer review only - http://bmjopen.bmj.com/site/about/guidelines.xhtml

Figure captions

Figure 1. Trainee knowledge analysis: user performance by DCM question theme. DCM questions assessed three themes - presentation, workup and management. Early stage trainees, those sitting finals/PLAB and the SRA, performed worse in questions relating to management versus more senior trainees taking the MRCGP. Performance for MRCGP trainees was more consistent. The average performance for each question theme was within 1 standard deviation (SD) of the mean.

Figure 2. Trainee knowledge analysis: user performance by disease. The mean user performance for DCM, neurology and cauda equina syndrome decreased with advancing question-bank. This trend was observed in most neurological pathologies. User performance of diabetes mellitus was more variable. * = there were no questions on cauda equina syndrome present in the SRA question-bank.

For peer review only - http://bmjopen.bmj.com/site/about/guidelines.xhtml

Table 1. Summary of gap analysis methods. This table shows the methods used in this study. Curricula and textbooks were screened by training stage to assess references to key search terms. An online question bank was used in knowledge assessment.

	Medical School	Foundation	GP Training	Metric
Curricula	UoM<br>UoC<br>Imperial	Foundation programme curriculum	MRCGP curriculum	References to search terms
Text Book	OHCM	OHFP	OHGP	References to search terms
Online Question Bank	PLAB<br>Medical finals	SRA	AKT	Performance in questions

For peer review only - http://bmjopen.bmj.com/site/about/guidelines.xhtml

Table 2. Selected diseases for textbook and curricula searches. The reasoning for each of the comparators is explained in this table. The search terms employed for the curricula searches are shown below this.

	Degenerative cervical myelopathy	Multiple sclerosis	Cauda equina syndrome	Diabetes mellitus
**Reasoning**	Disease of interest	A disease with a similar incidence and morbidity to degenerative cervical myelopathy. Selected as a clinico-epidemiological control [22]	Widely taught spinal emergency, selected as a spinal control.	Non-spinal control
**Search terms for curricula searches**	• Cervical myelopathy<br>• Cervical myeloradiculopathy<br>• Cervical stenosis<br>• Cervical compression<br>• Cervical herniation<br>• Cervical degeneration<br>• Ossification of posterior longitudinal ligament<br>• Spinal osteophytosis<br>• Spinal cord compression<br>• Spondylosis	• Multiple sclerosis<br>• Demyelinating disease<br>• Demyelination	• Cauda equina<br>• Saddle anaesthesia	• Diabetes mellitus<br>• Insulin dependent diabetes mellitus<br>• Type 1 diabetes mellitus<br>• Non insulin dependent diabetes mellitus<br>• Type 2 diabetes mellitus<br>• Maturity onset diabetes of young<br>• Gestational diabetes mellitus<br>• Late onset diabetes<br>• Maturity onset diabetes<br>• Insulin resistance<br>• Diabetic ketoacidosis<br>• Hyperosmolar hyperglycaemic state<br>• Hyperosmolar non ketotic coma

For peer review only - http://bmjopen.bmj.com/site/about/guidelines.xhtml

Table 3. Curricula analysis. Electronic copies of curricula were queried with relevant search terms (as shown in Table 2).

Curriculum	Number of references to term			
	**Degenerative cervical myelopathy**	**Multiple sclerosis**	**Cauda equina syndrome**	**Diabetes mellitus**
Undergraduate - PBL	0	3	1	43
Undergraduate – traditional	1	13	1	66
Undergraduate – integrated	3	15	0	30
Foundation Programme	0	0	0	0
MRCGP	0	4	2	113
**Total**	**4**	**32**	**4**	**252**
**Rank**	**3**	**2**	**3**	**1**

For peer review only - http://bmjopen.bmj.com/site/about/guidelines.xhtml

Table 4. Learning resource analysis. The number of words devoted to DCM and other diseases were determined. Importantly, only the words contained within the main section for the particular disease were considered.

**Resource**	**Word count devoted to**			
	**Degenerative cervical myelopathy**	**Multiple sclerosis**	**Cauda equina syndrome**	**Diabetes mellitus**
OHCM	870	1104	567	5165
OHFP	0	112	58	3599
OHGP	252	679	120	5736
Cumulative	1122	1895	745	14500
Modal rank	3	2	4	1

For peer review only - http://bmjopen.bmj.com/site/about/guidelines.xhtml

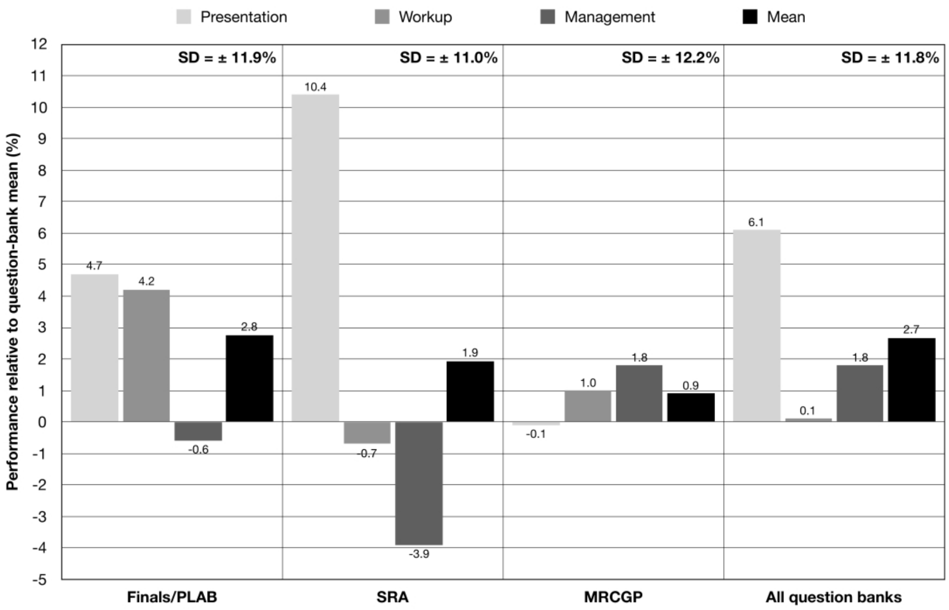

Figure 1

364x239mm (300 x 300 DPI)

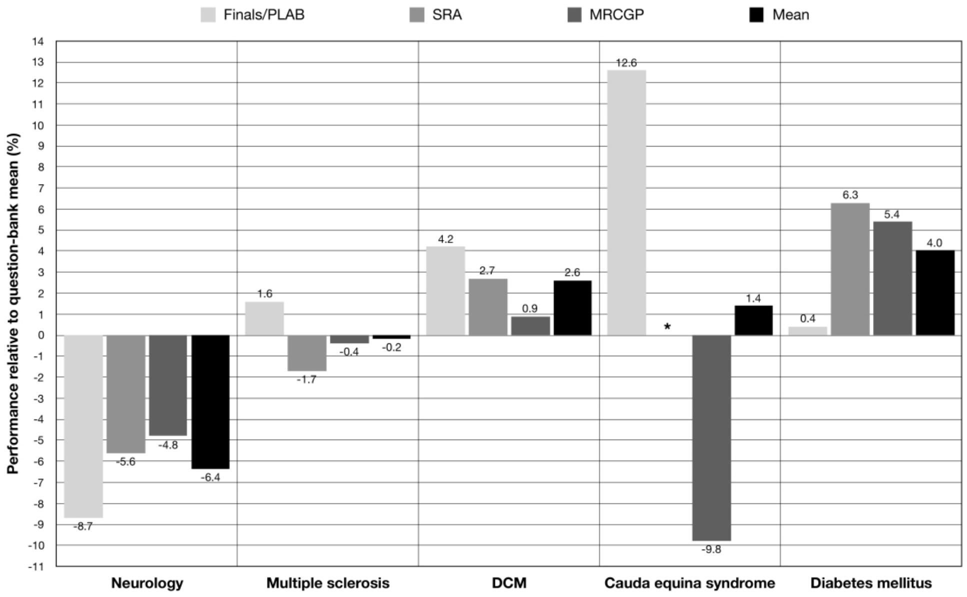

Figure 2

381x236mm (300 x 300 DPI)

Supplementary Table 1. Trainee knowledge analysis. A = number of questions used in question-bank; B = number of correct responses / number of attempts; C = correct response rate (%). Note that each individual EMQ had 3 distinct clinical scenarios.

Theme	Type	Question-bank									Cumulative results by theme		
		Finals/PLAB (beginner)			SRA (intermediate)			MRCGP (advanced)					
		A	B	C	A	B	C	A	B	C	A	B	C
Presentation	MCQ	1	658/1718	38.0%	3	407/1280	31.8%	3	1303/4667	27.9%	7	2367/7665	30.9%
	EMQ	2	5665/7914	73.2%	2	12589/17208	73.2%	2	6747/9246	73.0%	6	29286/40056	73.1%
	Both	3	6323/9632	65.6%	5	12996/18488	70.2%	5	8050/13913	57.9%	13	31653/47721	66.3%
Assessment	MCQ	4	3801/5378	70.7%	5	630/1198	52.6%	5	3960/5837	67.8%	14	8391/12413	67.6%
	EMQ	1	1618/2952	54.8%	2	8360/14019	59.6%	2	4784/8988	53.2%	5	14762/25959	56.9%
	Both	5	5419/8330	65.1%	7	8990/15217	59.1%	7	8744/14825	59.0%	19	23153/38372	60.3%
Management	MCQ	5	4653/6750	68.9%	5	792/1324	59.8%	5	3850/5612	68.6%	15	9295/13686	67.9%
	EMQ	1	3224/6306	51.1%	2	6939/12501	55.6%	2	4806/8871	54.2%	5	14969/27678	54.1%
	Both	6	7877/13056	60.3%	7	7731/13825	55.9%	7	8656/14483	59.8%	20	24264/41364	58.7%
Cumulative results by question-bank	MCQ	10	9112/13846	65.8%	13	1828/3802	48.1%	13	9113/16116	56.5%	36	20053/33764	59.4%
	EMQ	4	14792/22860	64.7%	6	27888/43728	63.8%	6	16337/27105	60.3%	16	59017/93693	63.0%
	Both	14	23904/36706	65.1%	19	29716/47530	62.5%	19	25450/43221	58.9%	52	79070/127457	62.0%

For peer review only

Passmedicine questions

Authors

1. Mueez Waqar
2. Jane Wilcock
3. Jayne Garner
4. Benjamin M Davies
5. Mark RN Kotter

Introduction

The following is a set of questions prepared for the degenerative cervical myelopathy education initiative. We would request a link to our website - www.myelopathy.org, our website to promote this initiative, below these questions.

Methods

Questions have been prepared as follows:

- Clinical presentation - focussing on differential diagnosis: 5 MCQs, 2 EMQs
- Assessment - focussing on examination and workup: 5 MCQs, 2 EMQs
- Management - focussing on referral pathways and follow-up: 5 MCQs, 2 EMQs

These questions are targeted to the following question banks:

- MRCGP
- MSRA
- Medical finals

Output

The aim of this initiative is to provide data on clinician knowledge on degenerative cervical myelopathy. We would like to analyse the following metrics based on these questions:

- Distribution of answers on 1st attempt
- Distribution of answers on 2nd attempt

In addition, we would like to compare the correct response rate of this sample to existing questions in the question-bank along the following themes:

- Multiple sclerosis
- Cauda equina syndrome
- Diabetes mellitus

For peer review only

Theme 1: Clinical presentation

A 65-year-old female reports progressive unsteadiness, mild weakness, pain and paraesthesias in her legs over the last 2-3 months. She reports no symptoms in her upper limbs and there is no history of trauma. Examination reveals increased lower limb tone with brisk reflexes. Which of the following is the most likely explanation for her symptoms?

A. Motor neuron disease
B. Degenerative cervical myelopathy
C. Multiple sclerosis
D. Syringomyelia
E. Cauda equina syndrome

Answer: B

Degenerative cervical myelopathy (DCM) refers to cervical spinal cord compression due to cervical spondylosis. The term replaces CSM (Cervical Spondylotic Myelopathy). Patients with DCM can present with predominantly lower limb symptoms. Examination findings, especially early on, are often subtle. DCM is the most common cause of non-traumatic paraparesis in developed countries and becomes more common with age [2]; it is estimated to have a prevalence of 5% in over 50-year-olds [3, 4]. In a series of patients presenting to Neurology outpatients, it was twice as common as Multiple Sclerosis [2]. Early diagnosis is key to ensuring good clinical outcomes. Currently most patients wait over 2 years for a diagnosis.

Incorrect options:

- Motor neuron disease is a disease of motor neurons, with mixed upper and lower motor features. Patients are classically described to have brisk reflexes in wasted and fasciculating limbs. As a rule, motor symptoms of weakness predominate and patients do not have sensory findings.
- Multiple sclerosis can also cause paraparesis. As a disease of the central nervous system, the paraparesis is usually associated with upper motor neuron signs. A first presentation of multiple sclerosis is most common between the ages of 20 and 40, younger than this patient. Degenerative Cervical Myelopathy is also more common with age and more common than MS overall, making that diagnosis more likely.
- Syringomyelia refers to the development of a syrinx in the spinal cord. It presents with a central cord syndrome, with predominantly upper limb signs. It is a relatively uncommon condition.
- Cauda equina syndrome results from compression of the cauda equina and classically includes leg weakness, saddle anaesthesia and sphincter disturbance. It is usually an acute syndrome with progressive signs. It is also a relatively uncommon condition.

References:

For peer review only

1. Kjaer P, Leboeuf-Yde C, Korsholm L, Sorensen JS, Bendix T. Magnetic resonance imaging and low back pain in adults: a diagnostic imaging study of 40-year-old men and women. Spine. 2005 May 15;30(10):1173–80.
2. Moore AP, Blumhardt LD. A prospective survey of the causes of non-traumatic spastic paraparesis and tetraparesis in 585 patients. Spinal Cord. 1997 Jun;35(6):361-7.
3. Kovalova I, Kerkovsky M, Kadanka Z, Kadanka Z, Nemec M, Jurova B, et al. Prevalence and Imaging Characteristics of Non-Myelopathic and Myelopathic Spondylotic Cervical Cord Compression. Spine. 2016 Aug 9.
4. Bednarik J, Kadanka Z, Dusek L, Kerkovsky M, Vohanka S, Novotny O, et al. Presymptomatic spondylotic cervical myelopathy: an updated predictive model. Eur Spine J. 2008 Mar;17(3):421–31.

For peer review only

A 73-year-old male presents with progressively worsening gait and urinary urgency. He is diagnosed with degenerative cervical myelopathy. Which ONE of the following is true regarding this condition?

A. Smoking is not a risk factor in isolation
B. Asians can have a different underlying aetiology than caucasians
C. Bowel and bladders symptoms are rare and should prompt consideration of cauda equina syndrome
D. Most patients present with a classic triad of neck pain, finger paraesthesias and weak legs
E. Family history is of limited value

Answer: B

Asian populations have a higher rate of ossification of the posterior longitudinal ligament (OPLL), which can result in myelopathy.

Degenerative cervical myelopathy (DCM) has a number of risk factors, which include smoking due to its effects on the intervertebral discs (A, false), genetics (option E, false) and occupation - those exposing patients to high axial loading [1].

The presentation of DCM is very variable (option D, false). Early symptoms are often subtle and can vary in severity day to day, making the disease difficult to detect initially. However as a progressive condition, worsening, deteriorating or new symptoms should be a warning sign.

DCM symptoms can include any combination of [1]:
- Pain (affecting the neck, upper or lower limbs)
- Loss of motor function (loss of digital dexterity, preventing simple tasks such as holding a fork or doing up their shirt buttons, arm or leg weakness/stiffness leading to impaired gait and imbalance
- Loss of sensory function causing numbness
- Loss of autonomic function (urinary or faecal incontinence and/or impotence) - these can occur and do not necessarily suggest cauda equina syndrome in the absence of other hallmarks of that condition

The most common symptoms at presentation of DCM are unknown, but in one series 50% of patients were initially incorrectly diagnosed and sometimes treated for carpal tunnel syndrome [2].

References
1. Baron EM, Young WF. Cervical spondylotic myelopathy: a brief review of its pathophysiology, clinical course, and diagnosis. Neurosurgery. 2007 Jan;60(1 Supp1 1):S35-41.

For peer review only

2. Behrbalk E, Salame K, Regev GJ, Keynan O, Boszczyk B, Lidar Z. Delayed diagnosis of cervical spondylotic myelopathy by primary care physicians. Neurosurg Focus. 2013 Jul;35(1):E1.

For peer review only

Stem 1: A 60-year-old gentleman with a background of hypertension and high cholesterol presents with a long history of neck pain, lower limb stiffness and urinary hesitancy. Which of the following is most likely?

Stem 2: A 56-year-old gentleman presents with lower limb stiffness and imbalance. His only past medical history of note is carpal tunnel syndrome that was diagnosed a year ago on clinical grounds and has been refractory to treatment with splints and steroid injections. Which of the following is most likely?

A. Cauda equina syndrome
B. Subacute combined degeneration of the cord
C. Degenerative cervical myelopathy
D. Parkinson's disease
E. Multiple sclerosis

Answer: C

The presentation of degenerative cervical myelopathy [DCM] is variable. Early symptoms are often subtle and can vary in severity day to day, making the disease difficult to detect initially. However as a progressive condition, worsening, deteriorating or new symptoms should be a warning sign.

DCM symptoms can include any combination of [1]:
- Pain (affecting the neck, upper or lower limbs)
- Loss of motor function (loss of digital dexterity, preventing simple tasks such as holding a fork or doing up their shirt buttons, arm or leg weakness/stiffness leading to impaired gait, imbalance and
- Loss of sensory function causing numbness
- Loss of autonomic function (urinary or faecal incontinence and/or impotence).

The most common symptoms at presentation of DCM are unknown, but in one series 50% of patients were initially incorrectly diagnosed and sometimes treated for carpal tunnel syndrome [2].

Other answers:
- Cauda equina syndrome results from compression of the cauda equina and classically includes leg weakness, saddle anaesthesia and sphincter disturbance. It is usually an acute syndrome with progressive signs. It does not cause leg stiffness.
- Subacute combined degeneration of the cord results from long-standing vitamin B12 deficiency, classically presenting as a posterior cord syndrome – with impaired proprioception. It can feature both upper and lower motor neuron signs. B12 deficiency can be associated with several neurological features. These include a myelopathy (classically the subacute combined degeneration of the cord), neuropathy and paraesthesias without neurological signs [3]. Subacute combined degeneration is extremely rare in developed countries, though in tropical countries it is frequently the commonest cause of non-traumatic myelopathy [4].

For peer review only

- Idiopathic Parkinson's disease is a tetrad of Tremor, Rigidity, Akinesia and Postural Instability (this can be remembered using the TRAP mneumonic). In the early stages pain is not a typical feature and it does not cause numbness.
- Multiple Sclerosis [MS] can have a variable presentation, with both sensory and motor symptoms and signs. Inflammatory changes are often present at multiple sites, which can cause symptoms at more than one site; a 'dissociated sensory loss', that is numbness at different and unlinked sites, is a hallmark of MS. Often patients will recall previous episodes of odd neurological deficits, which resolved. MS predominantly affects woman (3-4 times common) and usually presents before the age of 45.

References:

1. Baron EM, Young WF. Cervical spondylotic myelopathy: a brief review of its pathophysiology, clinical course, and diagnosis. Neurosurgery. 2007 Jan;60(1 Supp1 1):S35-41.
2. Behrbalk E, Salame K, Regev GJ, Keynan O, Boszczyk B, Lidar Z. Delayed diagnosis of cervical spondylotic myelopathy by primary care physicians. Neurosurg Focus. 2013 Jul;35(1):E1.
3. Kumar N1. Neurologic aspects of cobalamin (B12) deficiency. Handb Clin Neurol. 2014;120:915-26.
4. Pinto WB, de Souza PV, de Albuquerque MV, Dutra LA, Pedroso JL, Barsottini OG. Clinical and epidemiological profiles of non-traumatic myelopathies. Arq Neuropsiquiatr. 2016 Feb;74(2):161-5.

For peer review only

Select the most likely diagnosis for the following clinical scenarios. Each option can be chosen once or not at all.

A. Multiple sclerosis
B. Aortic aneurysm
C. Fractured clavicle
D. Peripheral neuropathy
E. Degenerative cervical myelopathy
F. Median nerve entrapment
G. Ulnar nerve entrapment
H. Lumbar canal stenosis
I. Saturday night palsy
J. Adhesive Capsulitis

1. A 70 year-old man has pain and weakness in both legs on walking. It settles with rest.
2. A 54 year-old female complains of right hand pain radiating into her thumb, index and middle finger. It often wakes her up from sleep.
3. A 54 year-old female presents with a loss of dexterity in both hands. She has been struggling to type at work and use her mobile phone. Her symptoms have been deteriorating gradually over the preceding months.

Answers

- 1H - lumbar spinal canal stenosis or vascular claudication from peripheral vascular disease are likely. Peripheral vascular disease is more common, but not an option here.
- 2F - Carpal tunnel syndrome results from median nerve compression at the wrist, within the carpal tunnel, and results in lower motor neuron signs, with thenar muscle wasting and weakness of the LOAF muscles (lateral lumbricals, opponens pollicis, abductor pollicis brevis and flexor policis brevis). Patients can have paraesthesias in the median nerve distribution, classically at night. Tinel's test and Phalen's test can be positive.
- 3E - Degenerative cervical myelopathy leads to loss of fine motor function in both upper limbs. There is a delay in diagnosis of degenerative cervical myelopathy, which is estimated to be >2 years in some studies [1]. It is most commonly misdiagnosed as carpal tunnel syndrome and in one study, 43% of patients who underwent surgery for degenerative cervical myelopathy, had been initially diagnosed with carpal tunnel syndrome [1].

References:

1. Behrbalk E, Salame K, Regev GJ, Keynan O, Boszczyk B, Lidar Z. Delayed diagnosis of cervical spondylotic myelopathy by primary care physicians. Neurosurg Focus. 2013 Jul;35(1):E1.

For peer review only

Select the most likely diagnosis for the following clinical scenarios. Each option can be chosen once or not at all.

A. Multiple sclerosis
B. Aortic aneurysm
C. Fractured clavicle
D. Peripheral neuropathy
E. Degenerative cervical myelopathy
F. Median nerve entrapment
G. Ulnar nerve entrapment
H. Lumbar canal stenosis
I. Saturday night palsy
J. Adhesive Capsulitis

1. A 60 year-old male presents with clumsy hands. He has been dropping cups around the house. His wife complains he doesn't answer his mobile as he struggles to use it. His symptoms have been gradually deteriorating over the preceding months.
2. A 32 year-old female presents with a 3 day history of altered sensation on her left foot and right forearm. On examination she has clonus in both legs and has hyperreflexia in all limbs.
3. A 45 year-old female presents with stiffness and pain in her left shoulder, which started around a month ago. She had a similar episode that resolved by itself. Examination reveals limited external rotation.

Answers

- 1E - Degenerative cervical myelopathy leads to loss of fine motor function in both upper limbs. There is a delay in diagnosis of degenerative cervical myelopathy, which is estimated to be >2 years in some studies [1]. It is most commonly misdiagnosed as carpal tunnel syndrome and in one study, 43% of patients who underwent surgery for degenerative cervical myelopathy, had been initially diagnosed with carpal tunnel syndrome [1].
- 2A - Multiple sclerosis (MS) can have a variable presentation, affecting both the sensory and/or motor systems. Inflammatory changes are often present at multiple sites, which can cause symptoms at more than one site; a 'dissociated sensory loss', that is numbness at different and unlinked sites, is a hallmark of MS. Often patients will recall previous episodes of odd neurological deficits, which resolved. MS predominantly affects woman (3-4 times common) and usually presents before the age of 45.
- 3J - Adhesive capsulitis or 'frozen shoulder' is most common in the fifth or sixth decade of life. Women are more likely to be affected than men. It is also more common in patients with diabetes mellitus.

References:

1. Behrbalk E, Salame K, Regev GJ, Keynan O, Boszczyk B, Lidar Z. Delayed diagnosis of cervical spondylotic myelopathy by primary care physicians. Neurosurg Focus. 2013 Jul;35(1):E1.

For peer review only

For peer review only

Theme 2: Assessment

A 58 year old gentleman presents with left sided paraesthesias affecting his thumb and first finger. He complains of grip weakness and dropping objects unintentionally. On examination, there is wasting over the thenar eminence. Which of the following signs would suggest a diagnosis other than carpal tunnel syndrome?

A. Positive Hoffman's sign
B. Thenar muscle wasting
C. Unilateral weakness of pincer grip
D. Positive Phalen's test
E. Positive Tinnel's test

Answer: A

A positive Hoffman's sign is a sign of upper motor neuron dysfunction and points to a disease of the central nervous system - in this case from the history degenerative cervical myelopathy [DCM] affecting the cervical spinal cord is most likely. To elicit it, the examiner should flick the patient's distal phalanx (usually of the middle finger) to cause momentary flexion. A positive sign is exaggerated flexion of the thumb.

DCM is often missed initially and there is a delay in the diagnosis of this condition by >2 years in some studies [1]. This is a problem as delayed treatment limits recovery. It is most commonly misdiagnosed as carpal tunnel syndrome and in one study, 43% of patients who underwent surgery for degenerative cervical myelopathy, had been initially diagnosed with carpal tunnel syndrome [1]. DCM is therefore an important differential in patients suspected to have Carpal Tunnel Syndrome [CTS].

CTS is a disease of the peripheral nervous system, resulting from median nerve compression at the wrist inside the carpal tunnel. It therefore affects only the aspects of the hand innervated by the median nerve:

- Sensation; Thumb / Index / Middle Finger. This typically manifests as intermittent pain or parasthesiae.
- Motor; 'LOAF Muscles'(lateral lumbricals, opponens pollicis, abductor pollicis brevis and flexor policis brevis). Motor signs are less commonly seen with presentations of CTS, but wasting of the thenar eminence may be present.

Tinel's test and Phalen's test can be positive, but not always. Both tests aim to increase the pressure within the carpal tunnel, to try to exacerbate symptoms; Tinel's test via tapping on it and Phalen's test by sustained full flexion of the wrist.

In focal central nervous system disorders, like DCM, examination features are known to have low sensitivity but high specificity [2]. As a disease of the cervical spinal cord, DCM can affect the sensory, motor and autonomic nervous systems from the neck downwards. Motor signs will be upper motor neuron signs such as increased toned, hyper-reflexia and pyramidal weakness. Note that the neurological signs of DCM are

For peer review only

often subtle initially and easily missed, but as a progressive condition they are likely to get worse [3]. Therefore detecting early DCM can be challenging. A high index of suspicion, alongside a comprehensive neurological examination and monitoring for progression is required.

References:

1. Behrbalk E, Salame K, Regev GJ, Keynan O, Boszczyk B, Lidar Z. Delayed diagnosis of cervical spondylotic myelopathy by primary care physicians. Neurosurg Focus. 2013 Jul;35(1):E1.
2. Nicholl DJ, Appleton JP. Clinical neurology: why this still matters in the 21st century. Journal of Neurology, Neurosurgery & Psychiatry 2015;86:229-33.
3. Baron EM, Young WF. Cervical spondylotic myelopathy: a brief review of its pathophysiology, clinical course, and diagnosis. Neurosurgery. 2007 Jan;60(1 Supp1 1):S35-41.

For peer review only

Which of the following statements is most accurate regarding the usefulness of cervical spine radiographs (X-rays) in the assessment of degenerative cervical myelopathy (DCM)?

A. Cervical spine radiographs should be obtained in all patients suspected of having DCM.
B. Where DCM is suspected, AP (anteroposterior), lateral *and* oblique cervical spine radiographs should be requested
C. Cervical spine radiographs are a useful first line investigation where a diagnosis of DCM is suspected
D. Cervical spine radiographs have a low sensitivity but high specificity for DCM
E. Cervical spine radiographs cannot diagnose DCM

Answer: E

Radiographs are of limited value where a diagnosis of degenerative cervical myelopathy is suspected [1] as they cannot visualise the soft tissue, such as the spinal cord.

Spine radiographs have a high sensitivity, but limited specificity to diagnose most spinal conditions. Oblique spine radiographs are usually requested in the lumbar spine region to pick up defects in the pars interarticularis. They have no value in setting of DCM.

The finding of spondylosis is common in spinal x-rays of adults over 40 [2]. Its absence does not exclude neural compression.

Degenerative Cervical Myelopathy [DCM] is spinal cord compression due to degenerative changes of the surrounding spinal structures; e.g. from disc herniation, ligament hypertrophy or calcification, or osteophytes. Therefore in order to visualise these structures, a MRI is gold standard and first line.

Again the presence of such degenerative changes is common on MRI; in one study, 57% of patients older than 64 years of age had disc bulging, though only 26% had spinal cord compression [3]. Therefore a diagnosis of DCM requires the finding of MRI compression in concert with appropriate signs and symptoms.

References
1. Nouri A, Tetreault L, Singh A, Karadimas SK, Fehlings MG. Degenerative Cervical Myelopathy: Epidemiology, Genetics, and Pathogenesis. Spine (Phila Pa 1976). 2015 Jun 15;40(12):E675-93.
2. Baron EM, Young WF. Cervical spondylotic myelopathy: a brief review of its pathophysiology, clinical course, and diagnosis. Neurosurgery. 2007 Jan;60(1 Supp1 1):S35-41.
3. Teresi LM, Lufkin RB, Reicher MA, Moffit BJ, Vinuela FV, Wilson GM, Bentson JR, Hanafee WN: Asymptomatic degenerative disk disease and spondylosis of the cervical spine: MR imaging. Radiology 164:83–88, 1987.

For peer review only

For peer review only

A 67-year-old male undergoes investigations for bilateral paraesthesia in the radial aspects of both hands, over the thumbs and first fingers. He also has paraesthesia in the lateral aspects of both forearms and lower limb spasticity. Blood tests reveal a HBA1c of 46. He undergoes nerve conduction studies and EMG with evidence of denervation. Which ONE of the following diagnoses is most likely?

A. Bilateral carpal tunnel syndrome
B. Degenerative cervical myelopathy
C. Multiple sclerosis
D. Syringomyelia
E. Diabetic neuropathy

Answer: B

This patient's twitches are probably fibrillations, a sign of lower motor neuron dysfunction. This is confirmed on the neurophysiology report, with evidence of denervation. His symptoms are predominantly in the C6 dermatome distribution bilaterally. Although median nerve compression at the elbow bilaterally could in theory produce his symptoms, it would be less likely to explain his symptoms given his age. He is likely to have degenerative cervical myelopathy. This condition is associated with a delay in diagnosis, estimated to be >2 years in some studies [1].

Patients with degenerative cervical myelopathy can present with a number of problems [2]:

- Pain/stiffness: affecting the neck, upper and/or lower limbs. L'hermitte's sign is a sharp pain radiating down the spine on flexion of the neck, which is classically associated with multiple sclerosis, though it can occur in cervical myelopathy.
- Loss of function: Clumsiness (e.g. can't do shirt buttons, hold cup), leg weakness leading to impaired gait, imbalance and falls.
- Sphincter disturbance: this can range from frequency and urgency to incontinence.

Neurological examination can reveal lower motor neuron signs at the level of the lesion and upper motor neuron signs below. Note that neurological signs can be subtle and a high degree of suspicion is needed [2].

The other answers in this question are unlikely for the following reasons:

- A: bilateral carpal tunnel syndrome would not cause forearm symptoms. Carpal tunnel syndrome results from median nerve compression at the wrist and results in a lower motor neuron picture, with thenar muscle wasting and weakness of the LOAF muscles (lateral lumbricals, opponens pollicis, abductor pollicis brevis and flexor policis brevis). Tinel's test and Phalen's test can be positive.
- C: multiple sclerosis (MS) is rare in this age group. MS predominantly affects woman (3-4 times common) and usually presents before the age of 45. It can have a variable presentation, affecting both the sensory and/or motor systems. Inflammatory changes are often present at multiple sites, which can cause symptoms at more than one site; a 'dissociated sensory loss', that is numbness at different and unlinked

For peer review only

sites, is a hallmark of MS. Often patients will recall previous episodes of odd neurological deficits, which resolved.

- D: Syringomyelia refers to the development of a syrinx in the spinal cord. It presents with a central cord syndrome, with predominantly upper limb signs. It is a relatively uncommon condition.
- E: His HBA1c is not within the diagnostic range of diabetes mellitus. Diabetes mellitus can cause a peripheral neuropathy presenting in a glove and stocking distribution, as well as neuropathy of peripheral nerves - mononeuritis multiplex.

References:

1. Behrbalk E, Salame K, Regev GJ, Keynan O, Boszczyk B, Lidar Z. Delayed diagnosis of cervical spondylotic myelopathy by primary care physicians. Neurosurg Focus. 2013 Jul;35(1):E1.
2. Baron EM, Young WF. Cervical spondylotic myelopathy: a brief review of its pathophysiology, clinical course, and diagnosis. Neurosurgery. 2007 Jan;60(1 Supp1 1):S35-41.

For peer review only

Which of the following investigations is the most important for diagnosing degenerative cervical myelopathy ?

A. Nerve conduction studies and EMG
B. MRI Cervical spine
C. CT myelogram
D. CT C-spine
E. AP and lateral C-spine radiographs

Answer: B

An MRI of the cervical spine is the gold standard test where cervical myelopathy is suspected. It may reveal disc degeneration and ligament hypertrophy, with accompanying cord signal change.

Other answers:

- CT imaging is reserved for patients with contraindications to magnetic resonance imaging. A CT myelogram is the first line investigation in this case.
- Radiographs are not clinically useful in the workup of these patients, though osteoarthritic changes (e.g. osteophytes) can be visible if they are performed.
- Other investigatons (e.g. nerve conduction studies, EMG) may be performed when the clinical picture is unclear. These can help to exclude mononeuropathies and other lower motor neuron disorders. However, where there is strong clinical suspicion and the diagnosis is suspected, an MRI of the cervical spine should be performed.

For peer review only

A 60-year-old gentleman with a background of lumbar spondylosis and chronic back pain presents with gradually worsening bilateral upper limb paraesthesias and leg stiffness. Which one of the investigations below is diagnostic for his likely condition?

A. Nerve conduction studies and EMG
B. MRI Cervical spine
C. MRI Lumbar Spine
D. CT C-spine
E. AP and lateral C-spine radiographs

Answer: B

The presence of upper limb neurological symptoms indicates that there is pathology either within his cervical spinal cord or brain. Brain disease is more likely to cause unilateral problems.

A MRI lumbar spine would therefore not provide a unifying diagnosis here.

In the context of known lumbar degenerative spine, degenerative cervical myelopathy is the number one differential for this presentation. An MRI of the cervical spine is the gold standard test where cervical myelopathy is suspected. It may reveal disc degeneration and ligament hypertrophy, with accompanying cord signal change. It is not uncommon for patients to suffer from tandem (cervical and lumbar) stenosis.

Other answers:

- CT imaging is reserved for patients with contraindications to magnetic resonance imaging. A CT myelogram is the first line investigation in this case
- Radiographs are not clinically useful in the workup of these patients, though osteoarthritic changes (e.g. osteophytes) can be visible if they are performed.
- Other investigatons (e.g. nerve conduction studies, EMG) may be performed when the clinical picture is unclear. These can help to exclude mononeuropathies and other lower motor neuron disorders. However, where there is strong clinical suspicion and the diagnosis is suspected, an MRI of the cervical spine should be performed.

For peer review only

Select the **most likely** positive examination finding for the following clinical scenarios. Each option can be chosen once, more than once or not at all.

A. Kernig's sign
B. Ankle Brachial Pressure Index
C. Tinel's test
D. Straight leg raise
E. Tongue fasciculations
F. Hoffman's sign
G. Limited external rotation of the shoulder
I. Hypothenar wasting
J. Limited internal rotation of the shoulder

1. A 70 year-old male with a background of diabetes and hypertension presents with pain and weakness in both legs on walking. It settles with rest.
2. A 54 year-old female complains of right hand pain radiating into her thumb, index and middle finger. It often wakes her up from sleep.
3. A 65 year-old female presents with a loss of dexterity in both hands. She has been struggling to type at work and use her mobile phone. Her symptoms have been deteriorating gradually over the preceding months.3

Answers

1 - B: this patient is likely to have peripheral vascular disease [PVD] given his background risk factors for this condition. The ankle brachial pressure index [ABPI] is a simple method of assessing the peripheral circulation. It is calculated by dividing systolic blood pressure in the ankle by the the systolic blood pressure in the arm. These are equal in health (ABPI = 1). The ABPI is reduced in PVD.

2 - C: this patient is likely to have carpal tunnel syndrome. This occurs due to median nerve entrapment beneath the flexor retinaculum. Clinical tests to raise carpal tunnel pressure can exacerbate symptoms and support a diagnosis. One such example is Tinel's test, includes tapping over the volar surface of the wrist joint i.e. over the carpal tunnel, may reproduce paraesthesias. A normal Tinel's test does not exclude carpal tunnel syndrome.

3 - F: this patient is likely to have degenerative cervical myelopathy [DCM], which is associated with upper motor neuron signs. Hoffman's sign is elicited by flicking the distal phalaynx of the middle finger to cause momentary flexion. A positive result is exaggerated flexion of the terminal phalanyx of the thumb. Patients with DCM often have subtle signs that are easily missed [1], but as a progressive condition, these are likely to get worse [2]. Whilst the sensitivity of signs is low (i.e. their absence does not rule out a problem), their specificity is high (i.e. there will be a problem). Therefore, in order to diagnose early DCM and improve patient outcomes, a high index of suspicion, alongside a 4comprehensive neurological examination and monitoring for progression is required.

Other signs mentioned:

For peer review only

- Kernig's sign refers to painful knee extension, from a position of hip flexion and knee flexion. It suggest meningeal irritation e.g. meningitis, subarachnoid haemorrhage.
- Straight leg raise: this is positively associated with radicular pathology such as disc herniation. The patient feels pain in the back when the leg is raised between 30-60 degrees.
- Limited external rotation is classically found in adhesive capsulitis. Patients have global restriction of shoulder movements, in at least two axes, though external rotation is usually the most affected and painful.

References:

1. Behrbalk E, Salame K, Regev GJ, Keynan O, Boszczyk B, Lidar Z. Delayed diagnosis of cervical spondylotic myelopathy by primary care physicians. Neurosurg Focus. 2013 Jul;35(1):E1.

2. Baron EM, Young WF. Cervical spondylotic myelopathy: a brief review of its pathophysiology, clinical course, and diagnosis. Neurosurgery. 2007 Jan;60(1 Supp1 1):S35-41.

For peer review only

Select the **most likely** positive examination finding for the following clinical scenarios. Each option can be chosen once, more than once or not at all.

A. Kernig's sign
B. Ankle Brachial Pressure Index
C. Tinel's test
D. Straight leg raise
E. Tongue fasciculations
F. Hoffman's sign
G. Limited external rotation of the shoulder
I. Hypothenar wasting
J. Limited internal rotation of the shoulder

1. A 60 year-old male presents with clumsy hands. He has been dropping cups around the house. His wife complains he doesn't answer his mobile as he struggles to use it. His symptoms have been gradually deteriorating over the preceding months.
2. A 32 year-old female presents with a 3 day history of altered sensation of her left foot and right forearm. She had an episode of visual loss a few months ago and says her friends have noted her eyes be flickery and jerky.
3. A 45 year-old female presents with stiffness and pain in her left shoulder, which started around a month ago. She had a similar episode that resolved by itself.

Answers

1 - F: this patient is likely to have degenerative cervical myelopathy [DCM], which is associated with upper motor neuron signs. Hoffman's sign is elicited by flicking the distal phalaynx of the middle finger to cause momentary flexion. A positive result is exaggerated flexion of the terminal phalanyx of the thumb. Patients with DCM often have subtle signs that are easily missed [1], but as a progressive condition, these are likely to get worse [2]. Whilst the sensitivity of signs is low (i.e. their absence does not rule out a problem), their specificity is high (i.e. there will be a problem). Therefore, in order to diagnose early DCM and improve patient outcomes, a high index of suspicion, alongside a comprehensive neurological examination and monitoring for progression is required.

2 - F: this patient is likely to have Multiple Sclerosis (MS). As a disease of the central nervous system, MS is usually associated with only upper motor neuron signs such as Hoffman's sign (see above). The patient's visual loss was probably secondary to optic neuritis, a common presentation of MS. Cerebellar signs are particularly common with MS and include nystagmus, which is likely to be the jerky eye movements noted by her friends.

3 - G: this patient is likely to have adhesive capsulitis. Patients have global restriction of shoulder movements, in at least two axes, though external rotation is classically described as the most affected and painful.

Other signs mentioned:

- Kernig's sign refers to painful knee extension, from a position of hip flexion and knee flexion. It suggest meningeal irritation e.g. meningitis, subarachnoid haemorrhage.

For peer review only

- Straight leg raise: this is positively associated with radicular pathology such as disc herniation. The patient feels pain in the back when the leg is raised between 30-60 degrees.
- The ankle brachial pressure index [ABPI] is a simple method of assessing the peripheral circulation. It is calculated by dividing systolic blood pressure in the ankle by the the systolic blood pressure in the arm. These are equal in health (ABPI = 1). The ABPI is reduced in peripheral vascular disease.
- Tinel's test includes tapping over the volar surface of the wrist joint i.e. over the carpal tunnel. This can reproduce paraesthesias in patients with carpal tunnel syndrome.

References:

1. Behrbalk E, Salame K, Regev GJ, Keynan O, Boszczyk B, Lidar Z. Delayed diagnosis of cervical spondylotic myelopathy by primary care physicians. Neurosurg Focus. 2013 Jul;35(1):E1.
2. Baron EM, Young WF. Cervical spondylotic myelopathy: a brief review of its pathophysiology, clinical course, and diagnosis. Neurosurgery. 2007 Jan;60(1 Supp1 1):S35-41.

For peer review only

Theme 3: Management and follow-up

A 75-year old gentleman presents with a short history of neck pain, paraesthesia in his finger tips and progressive leg weakness. Following a MRI scan of his spine, he is diagnosed with degenerative cervical myelopathy due to a C4/5 disc prolapse. Which of the following is the most appropriate management?

A. Cervical decompressive surgery
B. Cervical nerve root injection
C. Analgesia and referral to physiotherapy
D. Analgesia and review in 4 weeks time
E. Analgesia, a hard cervical collar and review in 4 weeks

Answer: A

All patients with degenerative cervical myelopathy should be urgently referred for assessment by specialist spinal services (neurosurgery or orthopaedic spinal surgery). This is due to the importance of early treatment. The timing of surgery is important, as any existing spinal cord damage can be permanent. Early treatment (within 6 months of diagnosis) offers the best chance of a full recovery but at present, most patients are presenting too late. In one study, patients averaged over 5 appointments before diagnosis, representing >2 years [1].

Currently, decompressive surgery is the only effective treatment. It has been shown to prevent disease progression. Close observation is an option for mild stable disease, but anything progressive or more severe requires surgery to prevent further deterioration. Physiotherapy should only be initiated by specialist services, as manipulation can cause more spinal cord damage.

Prompt diagnosis and onward referral are therefore key to ensuring good outcome for your patients. There are national initiatives to raise awareness of the condition to try and improve referral times (www.myelopathy.org). All of the other listed options in this question do not control the patient's primary pathology.

References:
1. Behrbalk E, Salame K, Regev GJ, et al. Delayed diagnosis of cervical spondylotic myelopathy by primary care physicians. Neurosurg Focus 2013;35:E1. doi:10.3171/2013.3.FOCUS1374

For peer review only

A 65-year old gentleman with a background of osteoarthritis and previous cervical laminectomy for degenerative cervical myelopathy presents with a 2-month history of worsening gait instability and urinary urgency. Which of the following is the most likely explanation for his symptoms?

A. Transverse myelitis
B. Recurrent degenerative cervical myelopathy
C. Multiple sclerosis
D. Cauda equina syndrome
E. Spinal metastases

Answer: B

- Postoperatively, patients with cervical myelopathy require ongoing follow-up as pathology can "recur" at adjacent spinal levels, which were not treated by the initial decompressive surgery. This is called adjacent segment disease. Furthermore, surgery can change spinal dynamics increasing the likelihood of other levels being affected. Patients sometimes develop mal-alignment of the spine, including kyphosis and spondylolisthesis, and this can also affect the spinal cord. All patients with recurrent symptoms should be evaluated urgently by specialist spinal services.
- Transverse myelitis usually presents more acutely than in this case, with a sensory level and upper motor neuron signs below the level affected. It can occur in patients with multiple sclerosis or Devic's disease (neuromyelitis optica). These patients tend to also have features such as optic neuritis.
- Cauda equina syndrome results from compression of the cauda equina and classically includes leg weakness, saddle anaesthesia and sphincter disturbance. This gentleman's history is much more likely to be in keeping with recurrent cervical myelopathy, given his background and given the subacute presentation
- Spinal metastases are uncommon, especially in a patient without a known primary. Given previous DCM, recurrence is more likely.

For peer review only

A 70 year old man has decompressive surgery for degenerative cervical myelopathy. Three years later he presents with neck pain and hand paraesthesias. Which one of the following management strategies is recommended?

A. Trial of neuropathic analgesia and cervical nerve root injections
B. Investigate with nerve conduction studies and EMG in the first instance
C. Urgent AP/lateral cervical spine radiographs as an MRI scan is contraindicated
D. Urgent referral to spinal surgery or neurosurgery
E. Refer to physiotherapy services

Answer: D

Postoperatively, patients with cervical myelopathy require ongoing follow-up as pathology can "recur" at adjacent spinal levels, which were not treated by the initial decompressive surgery.

Recurrent symptoms should be treated with a high degree of suspicion. Although peripheral neuropathy can occur in any patient, this should not be the diagnosis that is the most strongly suspected as delays in diagnosis and treatment of DCM affect outcomes. Therefore, B is false.

All patients with recurrent symptoms should be evaluated urgently by specialist spinal services (A and E, false). Axial spine imaging is necessary and a MRI scan is first line. In patients unable to to have a MRI, CT or CT myelogram may be considered. AP and lateral radiographs are of limited use when myelopathy is suspected (C, false).

References

1. Kong L, Cao J, Wang L, Shen Y. Prevalence of adjacent segment disease following cervical spine surgery: A PRISMA-compliant systematic review and meta-analysis. Medicine (Baltimore). 2016 Jul;95(27):e4171.

For peer review only

A 65-year-old gentleman is referred to neurology outpatients with arm pain, stiffness and imbalance. Following investigations he is diagnosed with degenerative cervical myelopathy. Unfortunately, he misses his next outpatient clinic due to admission with acute coronary syndrome. He attends his GP 2 months later and mentions his ongoing neurological symptoms. Which of the following is the most important next step in his care?

A. Refer to spinal surgery or neurosurgery
B. Refer for cervical nerve root injections
C. Commence neuropathic analgesia
D. Reassure the patient of his diagnosis
E. Refer for physiotherapy

Answer: A

Management of patients with cervical myelopathy should be by specialist spinal services (neurosurgery or orthopaedic spinal surgery). Decompressive surgery is the mainstay of treatment and has been shown to stop disease progression (B, false). Close observation is an option for mild stable disease, but anything progressive or more severe requires surgery to prevent further deterioration. Pre-operative physiotherapy should only be initiated by specialist services, as manipulation can cause more spinal cord damage.

The timing of surgery is important, as any existing spinal cord damage can be permanent. Treatment within 6 months offers the best chance of making a full recovery. At present most patients wait more than 2 years for a diagnosis [1].

Other incorrect options:

- Neuropathic analgesia is important for symptomatic relief but will not prevent further cord damage.
- Physiotherapy does not replace surgical opinion, it can in fact cause more spinal cord damage in patients yet to receive surgical treatment. It should therefore only be initiated by specialist services.

1. Behrbalk E, Salame K, Regev GJ, Keynan O, Boszczyk B, Lidar Z. Delayed diagnosis of cervical spondylotic myelopathy by primary care physicians. Neurosurg Focus. 2013 Jul;35(1):E1.

For peer review only

A 67-year old male recently attended A&E, with a 3 month history of bilateral paraesthesias and twitching affecting the thumb, first finger and lateral forearm. He denied any trauma. A MRI scan of his spine was performed and revealed cervical canal stenosis with mild cord compression. He was discharged and advised to see his GP for follow-up. Which of the following is the most appropriate initial step in management?

A. Refer to spinal surgery services
B. Refer for locally commissioned cervical root injections and review after 6 weeks
C. Enlist on the weekly minor ops clinic for carpal tunnel decompression
D. Commence neuropathic analgesia in the first instance and consider surgical evaluation if this does not work
E. Refer to physiology services and review in 6 weeks

Answer: A

Bilateral median nerve dysfunction is very suggestive of a diagnosis of degenerative cervical myelopathy (DCM) rather than bilateral carpal tunnel syndrome (option C). DCM should be suspected in elderly patients presenting with limb neurology. His twitches are probably fibrillations, a sign of lower motor neuron dysfunction.

Degenerative cervical myelopathy is associated with a delay in diagnosis, estimated to be >2 years in some studies [1]. It is most commonly misdiagnosed as carpal tunnel syndrome and in one study, 43% of patients who underwent surgery for degenerative cervical myelopathy, had been initially diagnosed with carpal tunnel syndrome [1]. Management of these patients should be by specialist spinal services (neurosurgery or orthopaedic spinal surgery). Decompressive surgery is the mainstay of treatment and has been shown to stop disease progression. Physiotherapy and analgesia does not replace surgical opinion, though they may be used alongside (options D and E). Nerve root injections do not have a role in management (option B).

1. Behrbalk E, Salame K, Regev GJ, Keynan O, Boszczyk B, Lidar Z. Delayed diagnosis of cervical spondylotic myelopathy by primary care physicians. Neurosurg Focus. 2013 Jul;35(1):E1.

5

For peer review only

Select the best management option for the following clinical scenarios. Each option can be chosen once, more than once or not at all.

A) Arrange X-rays of the cervical spine
B) Refer to pain clinic
C) Trial of analgesia and re-review
D) Refer to spinal surgery
E) Refer for physiotherapy
F) Lifestyle modification and co-morbidity optimisation
G) Trial of a cervical collar
H) Refer for a minor ops procedure, division of flexor retinaculum
I) Refer to neurology
J) Refer to elderly care medicine
K) Refer to vascular surgery

1. A 70 year-old male with a background of diabetes and hypertension presents with pain and weakness in both legs on walking. It settles with rest. His most recent HBA1c is 56 and an ankle-brachial pressure index is calculated as 0.7.
2. A 35 year-old female who is 30 weeks pregnant complains of right hand pain radiating into her thumb, index and middle finger. It often wakes her up from sleep.
3. A 65 year-old female presents with neck pain and loss of dexterity in both hands. She has been struggling to type at work and use her mobile phone. Her symptoms have been deteriorating gradually over the preceding months.

Answers

1 - F: this patient has peripheral vascular disease, as evident by his ankle-brachial pressure index (ABPI). NICE guidance suggests that first line management should include lifestyle modification such as smoking cessation, weight loss, lipid modification, optimisation of diabetes mellitus/hypertension and antiplatelet therapy. A supervised exercise programme can also be arranged. Local guidelines vary on when referral to specialist care is needed, but typically this would be where conservative treatment fails after 3 months or the ABPI is below a defined threshold (e.g. <0.6).

2 - E: this patient is likely to have carpal tunnel syndrome. This occurs due to median nerve entrapment beneath the flexor retinaculum. It is more common in pregnant women due to the increase in oedema. NICE clinical knowledge summaries (CKS) recommend lifestyle measures (e.g. wrist ergonomic devices at work), as well as wrist splints (usually prescribed by physiotherapists), corticosteroid injections or referral for surgical management. Wrist splints can be helpful for nighttime symptoms, as in her case. Corticosteroid injections require local expertise that may or may not be present.

3 - D: this patient is likely to have degenerative cervical myelopathy. DCM is often missed initially and there is a delay in the diagnosis of this condition by >2 years in some studies [1]. Patients have predominantly upper motor neuron signs such as increased toned, hyper-reflexia and pyramidal weakness. Neurological signs are often subtle initially and easily missed, but as a progressive condition they are likely to get worse [2]. Management of these patients should be by specialist spinal services

For peer review only

(neurosurgery or orthopaedic spinal surgery). An MRI scan is required for diagnosis. All patients should be assessed by a spinal surgeon.

References:

1. Behrbalk E, Salame K, Regev GJ, Keynan O, Boszczyk B, Lidar Z. Delayed diagnosis of cervical spondylotic myelopathy by primary care physicians. Neurosurg Focus. 2013 Jul;35(1):E1.
2. Baron EM, Young WF. Cervical spondylotic myelopathy: a brief review of its pathophysiology, clinical course, and diagnosis. Neurosurgery. 2007 Jan;60(1 Supp1 1):S35-41.

For peer review only

Select the best management option for the following clinical scenarios. Each option can be chosen once, more than once or not at all.

A) Arrange X-rays of the cervical spine
B) Refer to pain clinic
C) Trial of analgesia and re-review
D) Refer to spinal surgery
E) Refer for physiotherapy
F) Lifestyle modification and co-morbidity optimisation
G) Trial of a cervical collar
H) Refer for a minor ops procedure, division of flexor retinaculum
I) Refer to neurology
J) Refer to elderly care medicine
K) Refer to vascular surgery

1. A 60 year-old male presents with clumsy hands. He has been dropping cups around the house. His wife complains he doesn't answer his mobile as he struggles to use it. His symptoms have been gradually deteriorating over the preceding months.
2. A 32 year-old female presents with a 3 day history of altered sensation on her left foot and right forearm. She had an episode of visual blurring in her right eye a few months ago which resolved after a few days. Examination reveals brisk reflexes.
3. A 45 year-old female presents with stiffness and pain in her left shoulder, which started around a month ago. She had a similar episode that resolved by itself. Examination reveals global restriction of shoulder movement, particularly external rotation.

Answers

1 - D: this patient is likely to have degenerative cervical myelopathy. DCM is often missed initially and there is a delay in the diagnosis of this condition by >2 years in some studies [1]. Patients have predominantly upper motor neuron signs such as increased toned, hyper-reflexia and pyramidal weakness. Neurological signs are often subtle initially and easily missed, but as a progressive condition they are likely to get worse [2]. Management of these patients should be by specialist spinal services (neurosurgery or orthopaedic spinal surgery). An MRI scan is required for diagnosis. All patients should be assessed by a spinal surgeon.

2 - I: this patient is likely to have Multiple Sclerosis. Her visual loss was probably secondary to optic neuritis, a common ophthalmological association with multiple sclerosis. She should be referred to neurology.

3 - C: this patient is likely to have adhesive capsulitis (frozen shoulder). Patients have global restriction of shoulder movements, in at least two axes, though external rotation is classically described as the most affected and painful. Management of frozen shoulder is controversial and there is not much evidence to inform practice. In general, alternative diagnoses should be excluded and pain relief optimised. Gentle shoulder movement is encouraged and there is limited evidence for physiotherapy.

References:

1. Behrbalk E, Salame K, Regev GJ, Keynan O, Boszczyk B, Lidar Z. Delayed diagnosis of cervical spondylotic myelopathy by primary care physicians. Neurosurg Focus. 2013 Jul;35(1):E1.

2. Baron EM, Young WF. Cervical spondylotic myelopathy: a brief review of its pathophysiology, clinical course, and diagnosis. Neurosurgery. 2007 Jan;60(1 Supp1 1):S35-41.

For peer review only
